# Semisynthetic Ecdysteroid Cinnamate Esters and *tert*-Butyl Oxime Ether Derivatives with Trypanocidal Activity

**DOI:** 10.1021/acs.jnatprod.4c00811

**Published:** 2024-10-17

**Authors:** Márton B. Háznagy, Gábor Girst, Máté Vágvölgyi, Kaushavi Cholke, Sandhya Radha Krishnan, Jürg Gertsch, Attila Hunyadi

**Affiliations:** † Institute of Pharmacognosy, University of Szeged, Eötvös u. 6, H-6720 Szeged, Hungary; ‡ Institute of Biochemistry and Molecular Medicine, University of Bern, 3012 Bern, Switzerland; § Interdisciplinary Centre of Natural Products, University of Szeged, Eötvös u. 6, H-6720 Szeged, Hungary; ∥ HUN-REN-SZTE Biologically Active Natural Products Research Group, Eötvös u. 6, H-6720 Szeged, Hungary; ⊥ Graduate Institute of Natural Products, Kaohsiung Medical University, Shih-Chuan 1st Rd. 100, Kaohsiung 807, Taiwan

## Abstract

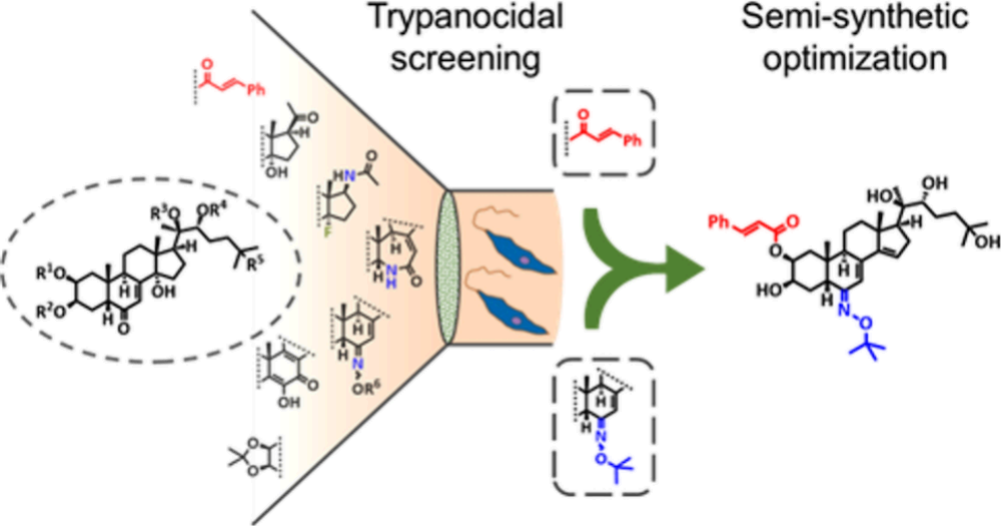

The parasite *Trypanosoma cruzi* is the causative agent of Chagas disease, a neglected tropical disease that affects the lives of millions of indigenous people in Latin America. As medications to treat Chagas disease are limited to the application of benznidazole and nifurtimox, which are not ideal treatments for the chronic stage of the disease, the search for new antichagasic drug candidates is an important need. Ecdysone has previously been shown to interfere with the life cycle of *T. cruzi*. Here, we report the biological profiling and subsequent semisynthetic structure optimization of 47 ecdysteroids against *T. cruzi* with the aim of identifying selective trypanocidal ecdysteroids. Two moderately trypanocidal pharmacophores were identified: ecdysteroids containing a 6-*tert*-butyl oxime ether and a cinnamic ester moiety. These functional groups were combined into the structures of four new semisynthetic ecdysteroids (**44**–**47**), among which **44** exerted potent and selective trypanocidal activity (IC_50_ < 2 μM). Cellular infection assays showed that ecdysteroid **44** potently and efficiently inhibited amastigote replication as determined by trypomastigote release after cellular infection with an IC_50_ of 2.7 ± 0.1 μM. The compound was similarly potent to benznidazole (IC_50_ = 3.8 ± 0.7 μM) and more than 5-fold more cytotoxic toward *T. cruzi* over RAW264.7 host macrophages. Overall, the ecdysteroid cinnamate ester **44** is a novel trypanocidal lead structure that needs to be further characterized in follow-up studies.

Chagas disease (CD) is a neglected tropical disease that is endemic in countries of Middle and South America, affecting more than 6 million patients worldwide, according to WHO estimations.^1,2^ It is caused by the hemoflagellate protozoan parasite *Trypanosoma cruzi*. The infection is primarily vector-borne, but it can be transmitted from mother to child via blood transfusion, and via the consumption of contaminated food. Its vectorial hosts are blood-sucking insects of the Triatominae family. The disease starts with an acute phase, which is frequently asymptomatic or associated with mild, moderate, or aspecific symptoms (fever, headache). The chronic phase is characterized by severe cardiac, neurological, and gastrointestinal symptoms.^3^ Benznidazole and nifurtimox are drugs available for the elimination of the parasite, but have unfavorable adverse effects and frequently observed resistance.^3^ Therefore, the introduction of novel and safe medicinal products would be essential. Natural products have served as interesting starting points to explore novel trypanocidal chemical scaffolds.^4−9^

The steroid derivative dehydroepiandrosterone was previously reported to exert moderate anti-infective activity against *T. cruzi*.^11^ Semisynthetic modifications of epiandrosterone enhanced the selective antiparasitic effects of this steroid, such as halogen incorporation to the C-16 and replacement of the C-3 hydroxyl group with an ester, sulfonate, or ether moiety.^10^ However, the possible androgenic side-effect of these compounds may obstruct their further development.^11^ Starting from additional natural steroids, novel, effective semisynthetic products have been synthesized, containing phenylpropane, thiosemicarbazide, or amide substituents.^12^ Dendrimers containing α-ethynyl-estradiol coupled to PAMAM-type dendrons displayed more potent antitrypanosomal activity than benznidazole.^13^ Physalins B and F secosteroids obtained from *Physalis angulata* L. have been reported to be effective against *T. cruzi*.^14^

Ecdysteroids are multihydroxylated steroidal compounds with an α,β-unsaturated ketone function on their B-ring. While they can exhibit anabolic, adaptogenic, neuroprotective, and other effects in mammals, the application of ecdysteroids does not exert any hormonal side-effects in vertebrates.^15^ In arthropods, however, they play a key role as moulting hormones,^16^ which makes them important regulators of the reproduction of the Chagas’ disease vector *Rhodnius prolixus*.^17^ Furthermore, it is of interest that ecdysone seems to interfere with *T. cruzi* development and aid the differentiation of the parasite in the midgut of *R. prolixus*.^18^ Based on the large chemical diversity of ecdysteroids, in this study we aimed to perform an antitrypanosomal screening on ecdysteroids followed by a semisynthetic structure optimization with the aim of improving efficiency of the initial hits.

## Results and Discussion

### 2.1 Synthesis and Chemical Characterization

First, the antiparasitic activity of 41 ecdysteroids (**1**–**41**) was screened against *T. cruzi* epimastigotes, and based on the hits, six additional compounds (**42**–**47**) were synthesized. These compounds represented a wide structural diversity of natural and semisynthetic ecdysteroids. Compounds **1**–**37** are of natural origin obtained during our previous phytochemical studies or semisynthetic derivatives synthesized in this work. Natural ecdysteroids included 20-hydroxyecdysone (20E, **1**), ajugasterone C (**2**), shidasterone (**3**), dacryhainansterone (**4**), and calonysterone (**5**). Semisynthetic ecdysteroids included side-chain-cleaved derivatives (**6**–**8**), dioxolane derivatives (**9**–**16**), nitrogen-containing ecdysteroids (**17**–**35**), fluorinated ecdysteroids (**36**, **37**), ecdysteroid cinnamic esters (**38**–**41**), oxime ethers (**42**, **43**), and oxime ethers with cinnamic ester moieties (**44**–**47**).

**Chart 1:**
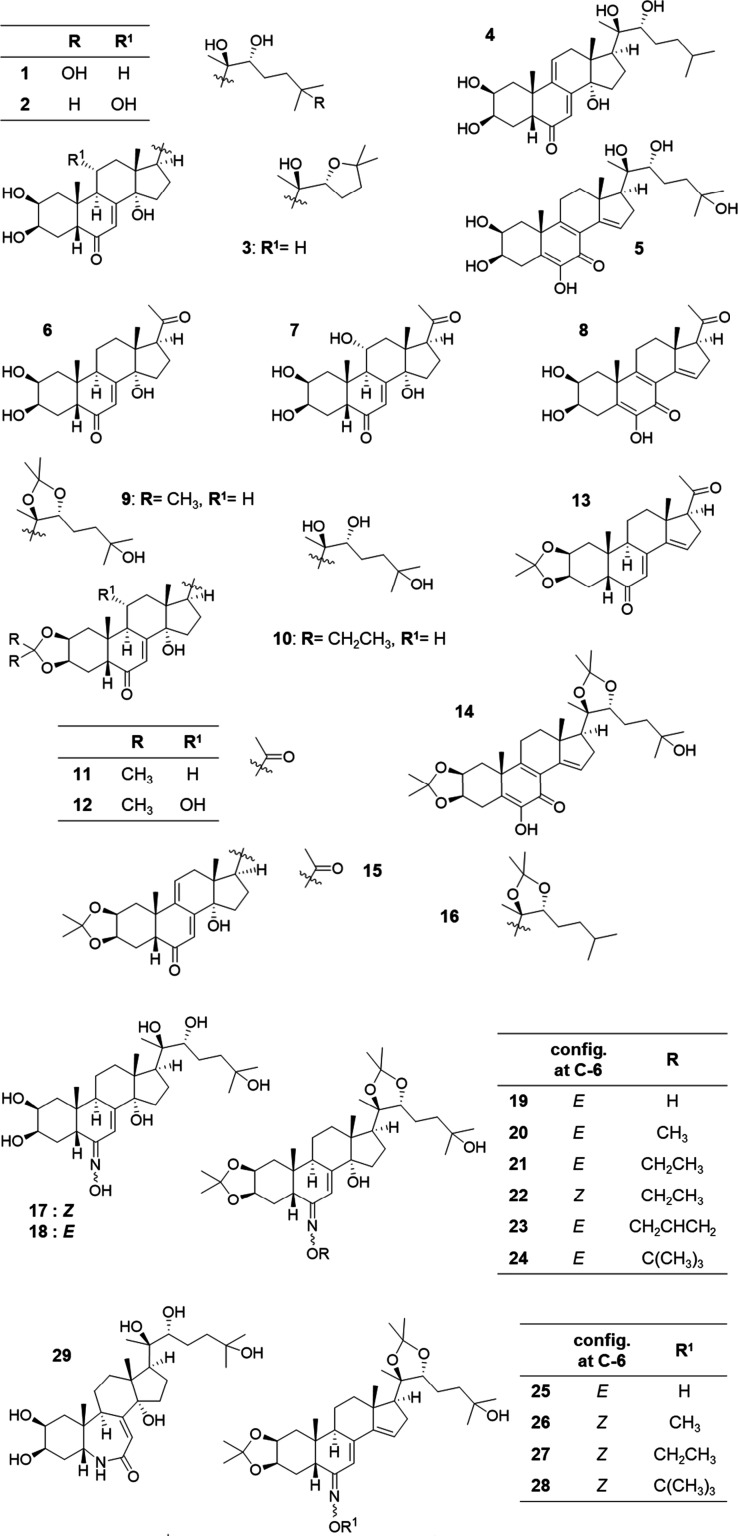

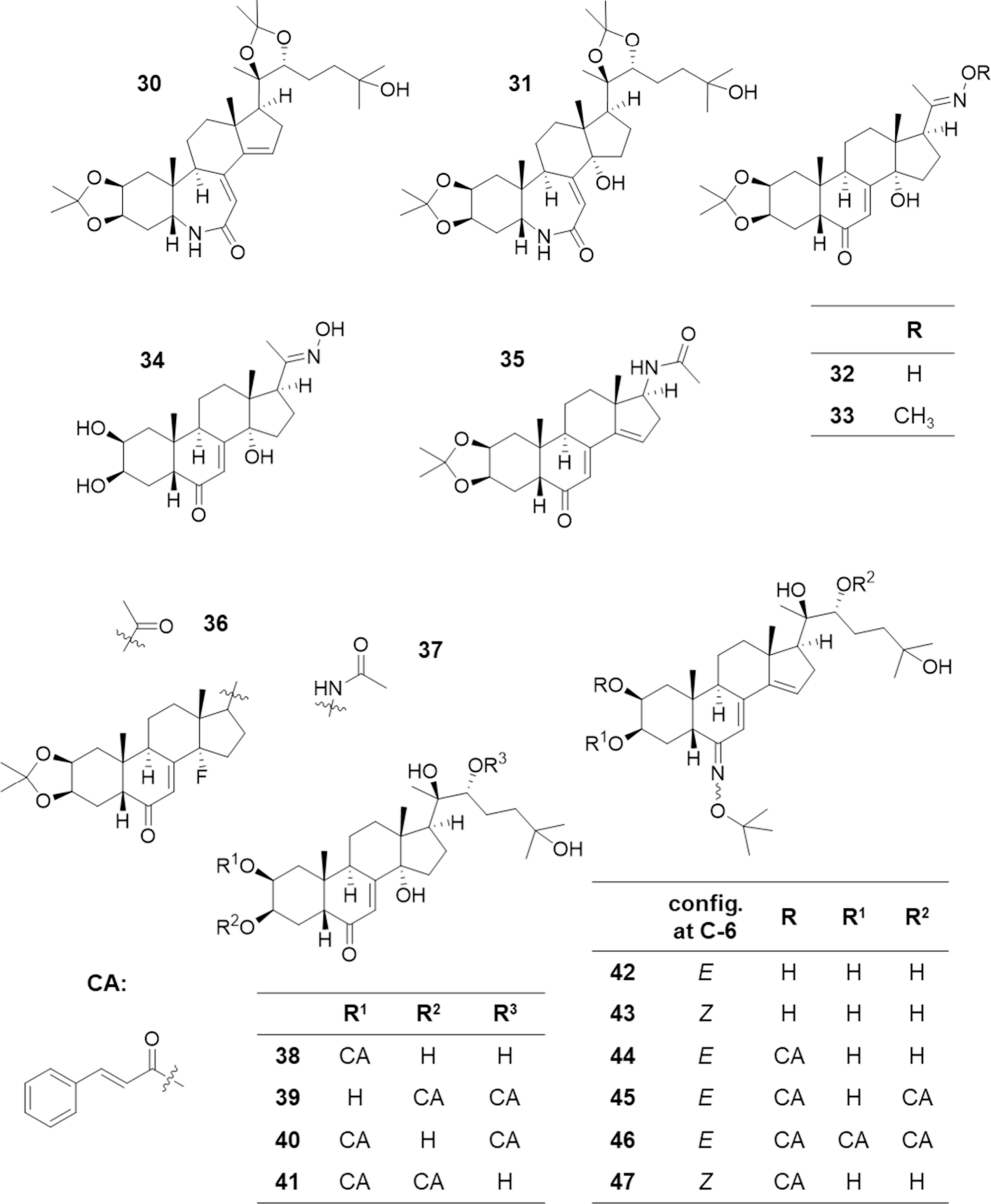


**Scheme 1:**
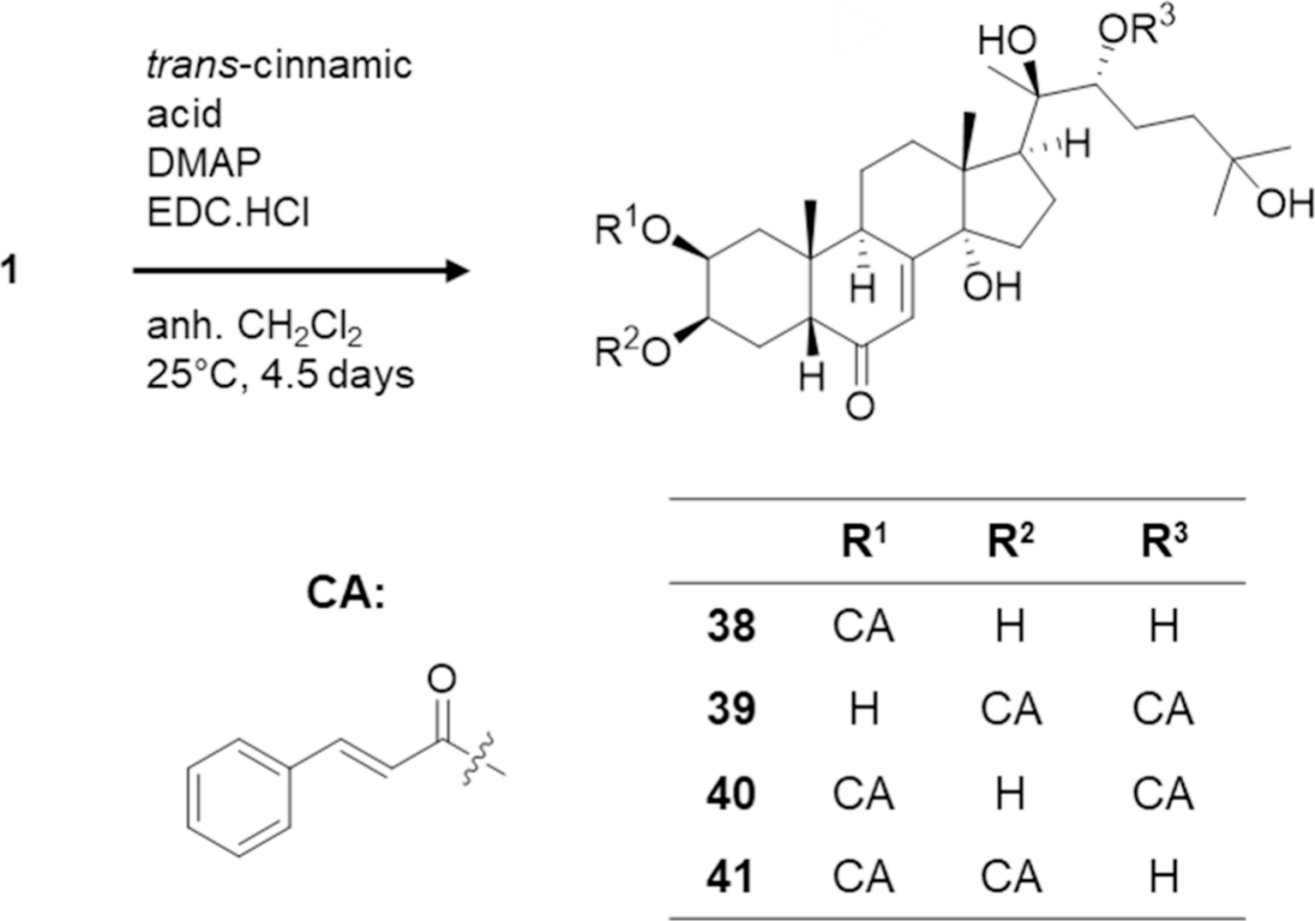
Preparation of Ecdysteroid Esters Starting from 20E to Obtain Compounds **38**–**41**

Compounds **39** and **41**–**47** are new ecdysteroids. The semisynthesis of these cinnamic esters was based on previous studies with some modifications.^19−21^ In the case of **38**–**41**, 20-hydroxyecdysone (**1**) was esterified by *trans*-cinnamic acid in the presence of EDC hydrochloride and 4-dimethylaminopyridine in anhydrous CH_2_Cl_2_. A two-step purification was performed by using normal-phase flash chromatography and reverse-phase HPLC to afford compounds **38**–**41** (Scheme 1). By using HR-MS and one- and two-dimensional NMR spectroscopy, it was found that the 20-hydroxyecdysone core remained intact, and the number and position of cinnamate groups were identified as follows: 2-monoester (**38**), 3,22-diester (**39**), 2,22-diester (**40**), and 2,3-diester (**41**).

**Scheme 2:**
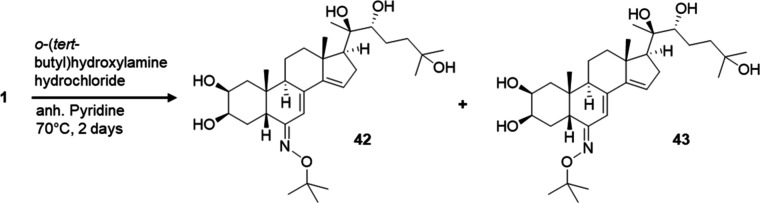
Preparation of Ecdysteroid 6-Oxime Ethers: *E* Isomer **42** (45.1% Yield), and *Z* Isomer **43** (13.6% Yield)

Inspired by the screening results for compounds **24**, **28**, **39**, and **40** (see below, Section 2.2), *tert*-butyl oxime ethers (**42** and **43**) and their cinnamic esters were produced. First, compound **1** was reacted with *O*-*tert*-butylhydroxylamine hydrochloride in anhydrous pyridine to form the appropriate oxime ether isomers. The oxime ether diastereomers were separated by RP-HPLC to afford compounds **42** and **43** (Scheme 2). In this reaction, the 14-OH groups got eliminated, leading to the formation of stachysterone B derivatives. The 14-OH group of ecdysteroids is sensitive to both acidic and alkaline pH changes and can be easily removed in the form of a water loss. This was observed in our previous work as well, where we set out to synthesize 6-oxime and oxime ether derivatives from 20-hydroxyecdysone 2,3;20,22-diacetonide (**9**).^24^ Neutralizing the reaction mixture by an alcoholic alkali solution allowed the preparation of products with an intact 14-OH group. However, when no subsequent neutralization step was applied, our transformations resulted exclusively in 14,15-anhydro products, suggesting the relevance of proper pH control in the chemoselectivity of the reactions. Another way to prevent 14-OH elimination is to start the reaction by using an alcoholic KOH solution to liberate the free base hydroxylamine from its hydrochloride salt and then add compound **1** under neutral conditions.^25^ In the current study, we used an aqueous KOH solution for reaction quenching, which is likely the reason for obtaining the 14,15-anhydro products. Moreover, the geometry of the oxime ether function was determined by comparing the NMR chemical shifts of H-5 and H-8, as well as C-5, C-6, and C-7, with our previously published data for 6-oxime ether derivatives of 20-hydroxyecdysone 2,3;20,22 diacetonide.^24^ Possibly due to steric reasons, *E* isomer **42** was formed at higher yields.

**Scheme 3:**
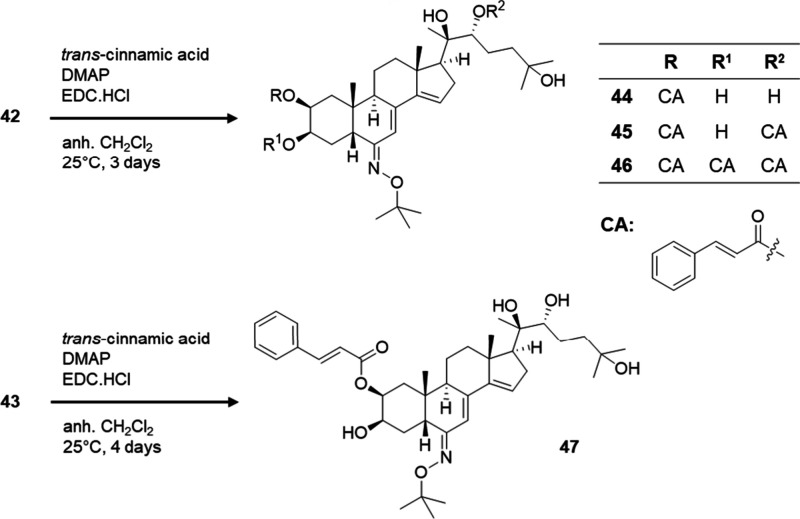
Esterification of Compounds **42** and **43** to Obtain **44**–**47**

Compound **42** was subsequently esterified by cinnamic acid and purified by preparative RP-HPLC to afford the 2-monoester (**44**), 2,22-diester (**45**), and 2,3,22-triester (**46**) derivatives. A similar reaction was carried out starting from **43** to obtain the 2-monoester **47** (Scheme 3). In this work, we followed a diversity-oriented approach; therefore, it was not our aim to improve the low yields (4.5–22.5%). While low isolated yields of the individual compounds were otherwise expected due to the similar reactivity of the several OH groups present in the starting material 20E, this allowed us to obtain several products from a single reaction and therefore have a larger variety of chemical scaffolds to evaluate structure–activity relationships. Nevertheless, it is worth mentioning that all monoesters contained this function at the C-2 position, which therefore seems a preferred position for esterification.

### 2.2 Biological Profiling on *T. cruzi* Epimastigotes and Trypomastigotes

The initial screening of ecdysteroids **1**–**41** was performed against *T. cruzi* insect-stage epimastigotes at a concentration of 5 μM, using benznidazole as a positive control (Figure S76, Supporting Information). Although the relevant parasite stages are amastigotes and trypomastigotes, antitrypanosomal compounds typically show selective toxicity toward epimastigotes as an initial readout.^26^ From this ecdysteroid library, the *tert*-butyl oxime ethers of 20E diacetonide (**24**) and stachysterone B (**28**), together with two cinnamic esters of 20E (**39** and **40**) demonstrated moderate selective toxicity, inhibiting epimastigote proliferation by ≥30% and exerting no (**24**, **28**, **39**) or very weak (**40**) cytotoxicity on CHO cells, which were used as proxy for mammalian host cells. Based on these results, two structural elements were identified as promising for antiparasitic activity, i.e., a *tert*-butyl oxime ether group in the C-6 and one or more cinnamic acid ester function(s) in the C-2 or C-3 and C-22 positions.

Ester and amide derivatives of cinnamic acid have previously been shown to possess antiparasitic activity related to leishmania and toxoplasma.^27−29^ Certain cinnamic acid derivatives were reported to exert antitrypanosomal activity, but the free acid forms were rather weak; for example, 4-methoxycinnamic acid (*p*-coumaric acid), 3,4,5-trimethoxycinnamic acid, and *trans*-2,4-dimethoxycinnamic acid showed IC_50_ values higher than 600 μM.^30^ Thus, ecdysteroid esters are unlikely to merely act as prodrugs of cinnamic acid, which is poorly active on *T. cruzi*. On the other hand, in a previous study on a series of aliphatic and aromatic esters of *p*-coumaric acid, pentyl-*p*-coumarate was found to exert trypanocidal activity with an IC_50_ value in the range of 5 μM.^31^

**Figure 1:**
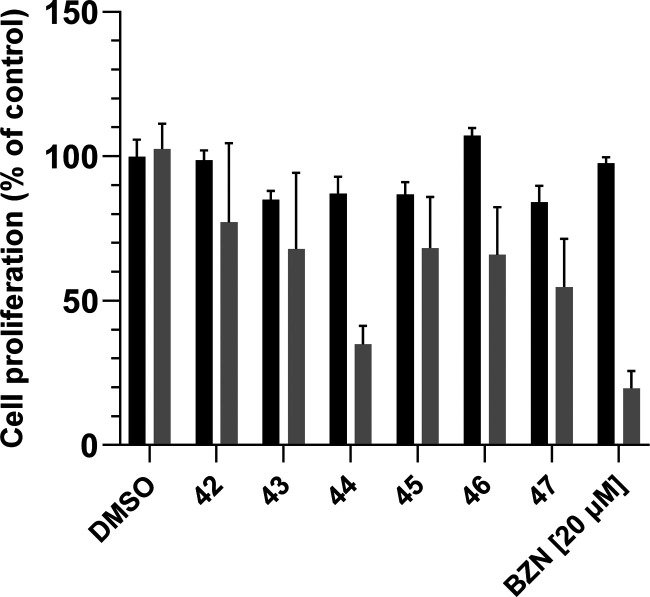
Antitrypanosomal and cytotoxic activity of compounds **42**–**47** (5 μM) against *T. cruzi* epimastigotes and Chinese hamster ovarian (CHO) cells measured by XTT and MTT, respectively, upon incubation of compounds for 72 h in the logarithmic proliferative phase. Benznidazole (20 μM) was used as a positive control. Data show mean values ± SD of three independent experiments each performed in triplicate. *, **, ***p < 0.005 (Student’s *t*-test).

Based on our first hits containing either a 6-*tert*-butyl oxime ether or at least one cinnamic ester moiety, we combined these tentatively identified pharmacophores into the structure of 20E and synthesized compounds **44**–**47** and the acetonide-free 6-*tert*-butyl oxime ethers **42** and **43** (see above, Section 2.1). The newly semisynthesized compounds were also tested against the epimastigote stage of *T. cruzi* at a 5 μM concentration (Figure 1).

Except for compounds **42** and **43**, i.e., those lacking a cinnamic ester moiety, all new ecdysteroids exerted at least 30% inhibition of epimastigote proliferation. Considering the whole library tested in this study (**1**–**47**), C-2 monoesters with a *tert*-butyl oxime ether at C-6 (**44** and **47**) were the most effective, and **44** was a potent and selective antitrypanosomal agent. This confirms that combining these two structural elements is a valid strategy to improve the antitrypanosomal potency of ecdysteroids.

**Figure 2:**
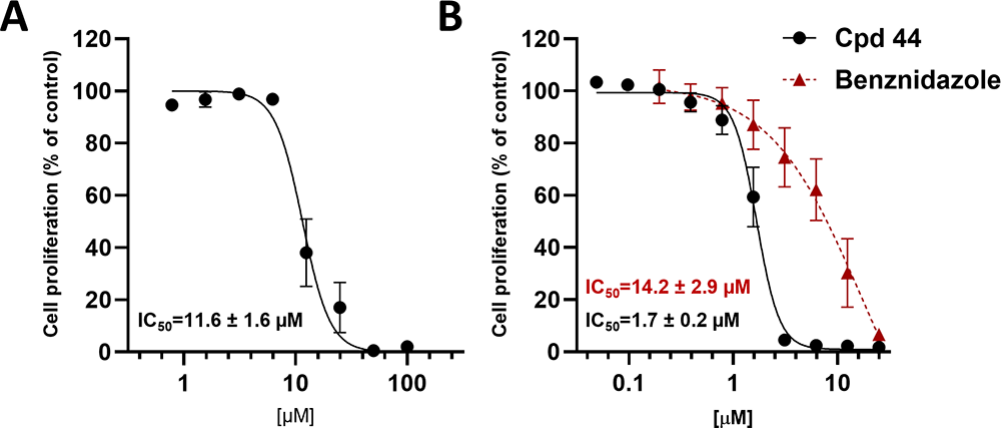
Selective trypanocidal effects of compound **44**. (A) Effects on cell proliferation (MTT assay) were assessed in RAW264.7 cells after 72 h of incubation showing an IC_50_ value of 11.6 ± 1.6 μM. (B) XTT assays on epimastigote proliferation yielded an IC_50_ value of 1.7 ± 0.2 μM for **44** and 14.2 ±2.9 μM for Benznidazole (BZN), which was used as a positive control. Data show mean values ± SD of at least 3 independent experiments each performed in triplicates.

**Figure 3:**
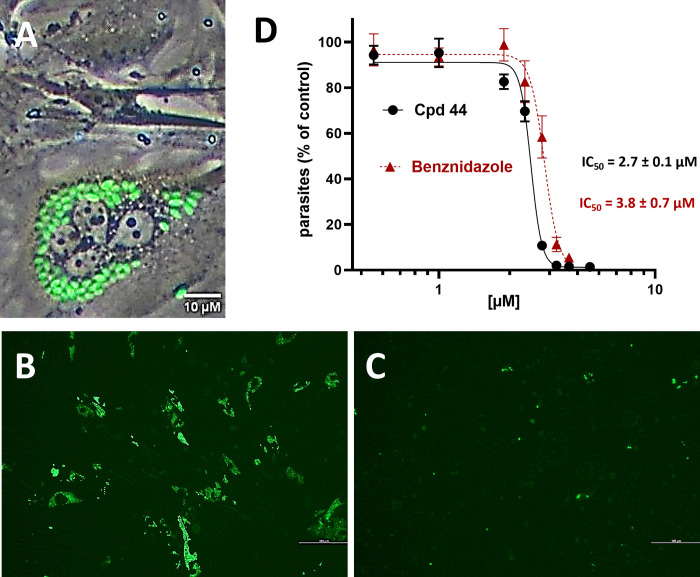
Compound **44** efficiently inhibits amastigote replication in host cells, leading to a reduction of parasite release from infected cells like the positive control benznidazole. (A) Representative brightfield/fluorescence microscopic image of CCL39 fibroblasts infected with *T. cruzi* showing amastigotes (green). (B) Representative fluorescence microscopic images of infected cells showing the GFPY fluorescence signal of the *T. cruzi* amastigotes (vehicle control). (C) Inhibition of amastigote replication (green) by compound **44** (5 μM) after 6 days of infection. (D) Inhibition curves of compound **44** and the positive control benznidazole on GFPY parasite release in trypomastigote-infected RAW264.7 cells measured by FACS after 6 days yielded an IC_50_ value of 2.7 ± 0.1 μM. Data show mean values ± SD of three independent experiments each performed in triplicates.

The trypanocidal effects of **44** are summarized in Figure 2. Compound **44** showed moderate cytotoxicity on the host cells, with an apparent selectivity of >5. Unlike the nitroheterocyclic benznidazole, which is a prodrug that needs activation by trypanosomal type I nitroreductase (NTRI) and is therefore more active in the amastigote stage,^32^ compound **44** efficiently inhibited the proliferation of *T. cruzi* epimastigotes with an IC_50_ value of 1.7 ± 0.2 μM (Figure 3) with more than 95% inhibition of trypomastigote release, showing an IC_50_ value of 2.7 ± 0.1 μM, thus having a comparable efficacy and potency to benznidazole (IC_50_ value of 3.8 ± 0.7 μM) in this assay. Based on these findings, we may point out some preliminary structure–activity relationships. It seems that the presence of an apolar moiety on the A-ring is necessary in addition to a bulky nitrogen-containing substituent on the B-ring. It is noteworthy that an unaltered ecdysteroid side chain (as in compounds **44**, **47**) is preferential over the corresponding 22-cinnamates (compounds **45**, **46**). It is also important to note that the oxime ether moiety with an *E* configuration (compound **44**) showed significantly higher activity compared to that with a *Z* configuration (compound **47**). On the other hand, a retained 14α-OH group (compound **24**) vs a Δ^14,15^ olefin (compound **28**) does not seem to make a significant difference concerning trypanocidal activity. Altogether, ecdysteroid 6-*tert*-butyl oxime ethers with a C-2-cinnamate moiety, as in compound **44**, are good starting points toward the development of potentially effective antitrypanosomal agents.

## Experimental Section

### General Experimental Procedures

Melting points were measured using an RK Tech melting point apparatus with a microscope. Optical rotations were measured applying a JASCO P-2000 polarimeter (JASCO International Co. Ltd., Hachioji, Tokyo, Japan) in MeOH or CHCl_3_. NMR spectra were recorded at 25 °C on a Bruker Avance DRX-500 NMR spectrometer (Bruker, Billerica, MA, USA) at 500 MHz (^1^H) and 125 MHz (^13^C). In general, 5–6 mg of the corresponding ecdysteroid was dissolved in acetone-*d*_6_ (**38**–**41** and **44**–**47**) or DMSO-*d*_6_ (**42**, **43**) and transferred to NMR tubes for recording spectra. Chemical structures of the derivatives were determined by employing comprehensive one- and two-dimensional NMR methods. Mass spectra were recorded on an Agilent 1100 LC-MS instrument (Agilent Technologies, Santa Clara, CA, USA) coupled with a Thermo Q-Exactive Plus orbitrap analyzer (Thermo Fisher Scientific, Waltham, MA, USA) used in positive ionization mode. Chromatographic purification of the compounds was carried out in one or two steps. For flash chromatography, a CombiFlash Rf+ Lumen instrument (TELEDYNE Isco, Lincoln, NE, USA) was used, which was equipped with diode array and ELS detection. The chromatographic purifications were carried out using prefilled Redisep NP-silica flash columns (TELEDYNE Isco) purchased from a commercial source. Analytical-scale reversed-phase HPLC methods were carried out on a Jasco HPLC instrument (Jasco International Co., Ltd., Hachioji, Tokyo, Japan) equipped with an MD-2010 Plus PDA detector to collect data in a range of 210–400 nm. The measurements were performed on a Gemini NX-C18 250 mm 4.6 mm column (Phenomenex Inc., Torrance, CA, USA) and on a Kinetex Biphenyl 250 mm 4.6 mm column (Phenomenex Inc.). Preparative HPLC separations were performed on an Armen Spot Prep II integrated HPLC purification system (Gilson, Middleton, WI, USA) with dual-wavelength detection applied. The separations were performed on a Gemini NX-C18 250 mm × 21.2 mm column (Phenomenex Inc.) and on a Kinetex Biphenyl 250 mm × 21.2 mm column (Phenomenex Inc.) applying a 15 mL/min flow and using different ratios of aqueous MeCN, MeOH, or THF. All reagents and solvents were purchased from Sigma-Aldrich (St. Louis, Mo, USA) and were used without any further purification. The chemical reactions were monitored by thin layer chromatography on Kieselgel 60F254 silica plates purchased from Merck (Merck KGaA, Darmstadt, Germany), and characteristic spots of the compounds were examined under UV light at 254 and 366 nm.

### Previously Isolated or Semisynthetised Ecdysteroids

Ecdysteroids **1**–**37** were isolated or semisynthetically synthesized in our previous studies. Compound **1** (20E) was purchased from Shaanxi KingsSci Biotechnology Co., Ltd. (Shanghai, People’s Republic of China), and recrystallized from EtOAc/MeOH, 2:1. Compounds **2** and **3** were obtained from a *C. arachnoidea* extract containing food supplement.^33^ Compounds **4** and **5** were previously isolated from a commercially available extract of *C. arachnoidea*.^34^ Compounds **6**–**37** were prepared by semisynthesis as reported before.^24,25,35−40^ Compounds **38**(22) and **40**(23) are known ecdysteroid derivatives; their spectroscopic data were in agreement with the literature.

### General Procedure for the Preparation of Cinnamic Acid Esters of **1**

To a suspension of **1** (1.00 g, 2.081 mmol) in dry CH_2_Cl_2_ (over molecular sieves, 250 mL) were added 4-dimethylamino pyridine (0.64 g, 5.239 mmol), *trans*-cinnamic acid (1.30 g 8.774 mmol), and *N*-ethyl-*N*′-(3-(dimethylamino)propyl)carbodiimide hydrochloride (EDC·HCl; 1.20 g, 6.26 mmol). The reaction mixture was stirred for 4.5 days at room temperature, and the initial suspension turned into a solution during the reaction process. When the reaction was completed, an aqueous saturated NaHCO_3_ solution and brine were poured into the solution and stirred for 5 min. The reaction mixture was extracted with CH_2_Cl_2_ (3 × 50 mL). The combined organic layer was washed with brine, dried (Na_2_SO_4_), filtered, and evaporated under reduced pressure. The crude product was purified via NP flash chromatography on RediSep silica 24 g gold in a CH_2_Cl_2_/MeOH system from 0 to 5% in 10 min and then in 5% MeOH for 40 min applying a 35 mL/min flow. The products needed to be purified further; thus we applied preparative RP HPLC methods to get the pure products. The mixture was separated into roughly pure fractions applying the MeCN/H_2_O (52/48) system on a biphenyl column. These steps provided compound **40** in its pure form. In the case of compound **38** MeCN/H_2_O (30/70), in the case of compound **39** MeCN/H_2_O (52/48), and in the case of compound **41** MeCN/H_2_O (50/50) was applied to get the pure compounds.

#### Compound **38**:

21.3 mg (1.7%) white solid; mp 154.5–156.4 °C; [α]^25^_D_ +14.4 (*c* 0.22, MeOH); HR-MS *m*/*z* 611.35634 [M + H]^+^ (calcd for C_36_H_51_O_8_^+^: 611.35785), *m*/*z* 633.33825 [M + Na]^+^ (calcd for C_36_H_50_O_8_Na^+^: 633.33979); HPLC purity 98.1%.

#### Compound **39**:

52.4 mg (3.4%) white solid; mp 155.2–157.4 °C; [α]^25^_D_ +55.5 (*c* 0.20, CHCl_3_); HR-MS *m*/*z* 741.39765 [M + H]^+^ (calcd for C_45_H_57_O_9_^+^: 741.39971), 763.37944 [M + Na]^+^ (calcd for C_45_H_56_O_9_Na^+^: 763.38165); HPLC purity 98.2%.

#### Compound **40**:

0.1603 g (10.4%) white solid; mp 159.0–161.0 °C [α]^25^_D_ +23.3 (*c* 0.21, CHCl_3_); HR-MS *m*/*z* 763.37927 [M + Na]^+^ (calcd for C_45_H_56_O_9_Na^+^: 763.38165); HPLC purity 96.5%.

#### Compound **41**:

0.0446 g (2.9%) white solid; mp 141.1–143.0 °C; [α]^25^_D_ −46.5 (*c* 0.21, CHCl_3_); HR-MS *m*/*z* 763.37900 [M + Na]^+^ (calcd for C_45_H_56_O_9_Na^+^: 763.38165); HPLC purity 99.4%.

### General Procedure for the Synthesis of Ecdysteroid-6-*O-tert*-butyl Oxime Ethers

To a solution of **1** (0.500 g, 1.040 mmol) in anhydrous pyridine (25 mL) was added *O*-*tert*-butylhydroxylamine hydrochloride (0.500 g, 3.981 mmol). The reaction mixture was stirred at 70 °C for 2 days. When the TLC indicated, the reaction was cooled to room temperature, quenched by the addition of an aqueous KOH solution (10 mL, 0.40 mM), and evaporated under reduced pressure. Water (20 mL) was poured into the residue and extracted with EtOAc (3 × 30 mL). The combined organic phase was washed with brine, dried (Na_2_SO_4_), filtered, and then concentrated to dryness. The crude product was purified via preparative RP-HPLC (Gemini NX-C_18_) in 35% MeCN/MeOH (9:1) in water. Two isomers (*E*/*Z*) were separated.

#### Compound **42** (*E* isomer):

250.31 mg (45.1%) white solid; mp 153.8–155.5 °C; [α]^25^_D_ −244.4 (*c* 0.21, MeOH); HR-MS *m*/*z* 534.3792 [M + H]^+^ (calcd for C_31_H_52_NO_6_^+^: 534.3789); HPLC purity 98.6%.

#### Compound **43** (*Z* isomer):

75.28 mg (13.6%) white solid; mp 138.2–140.1 °C; [α]^25^_D_ −268.3 (*c* 0.21, MeOH); HR-MS *m*/*z* 534.3792 [M + H]^+^ (calcd for C_31_H_52_NO_6_^+^: 534.3789); HPLC purity 98.4%.

### General Procedure for Preparing Cinnamic Esters of Compounds **42**

To a suspension of **42** (0.150 g, 0.281 mmol) in anhydrous CH_2_Cl_2_ (20 mL) were added 4-dimethylamino pyridine (0.089 g, 0.728 mmol), *trans*-cinnamic acid (0.167 g 1.124 mmol), and EDC·HCl (0.162 g, 0.843 mmol). The reaction mixture was stirred for 3 days at room temperature, and the initial suspension turned into a solution during the reaction process. When the reaction was completed, as indicated by TLC, water (10 mL) was poured into the solution and stirred for 5 min. Saturated NH_4_Cl was added to the reaction mixture; then it was extracted with EtOAc (3 × 20 mL). The combined organic layer was washed with brine, dried (Na_2_SO_4_), filtered, and evaporated under vacuum. The crude product was purified via RP-HPLC (biphenyl column) using 74% aqueous MeCN/THF (3:1). This separation step led to three fractions, which needed further purifications. In the case of compound **44**, 48% aqueous MeCN/THF (3:1), in the case of compound **45**, 56% aqueous MeCN/THF (3:1), and in the case of compound **46**, 70% aqueous MeCN/THF (3:1) were applied to obtain the pure products.

#### Compound **44**:

10.22 mg (5.5%) white solid; mp 145.3–147.3 °C; [α]^25^_D_ −166.4 (*c* 0.23, CHCl_3_); HR-MS *m*/*z* 664.4202 [M + H]^+^ (calcd for C_40_H_58_NO_7_^+^ 664.4208); HPLC purity 98.0%.

#### Compound **45**:

26.86 mg (12.1%) white solid; mp 167.0–169.0 °C; [α]^25^_D_ −162.7 (*c* 0.21, CHCl_3_); HR-MS *m*/*z* 794.46301 [M + H]^+^ (calcd for C_49_H_64_NO_8_^+^ 794.46264); HPLC purity 96.6%.

#### Compound **46**:

11.78 mg (4.5%) white solid; mp 145.0–147.0 °C; [α]^25^_D_ −174.2 (*c* 0.21, CHCl_3_); HR-MS *m*/*z* 924.50609 [M + H]^+^ (calcd for C_58_H_70_NO_9_^+^ 924.50451); HPLC purity 98.0%.

### General Procedure for Preparing Cinnamic Ester Starting from Compound **43**

To a suspension of **43** (59.33 mg, 0.111 mmol) in anhydrous CH_2_Cl_2_ (10 mL) were added 4-dimethylamino pyridine (35 mg, 0.286 mmol), *trans*-cinnamic acid (65.8 mg 0.444 mmol), and EDC·HCl (63.9 mg, 0.333 mmol). The reaction mixture was stirred for 4 days at room temperature, and the initial suspension turned into a solution during the reaction process. After 4 days, water (5 mL) was poured into the solution and stirred for 5 min. Saturated NH_4_Cl was added to the reaction mixture and was extracted with EtOAc (3 × 15 mL). The combined organic phase was washed with brine, dried (Na_2_SO_4_), filtered, and evaporated under reduced pressure. The crude product was purified by RP-HPLC (Kinetex biphenyl) in 45% aqueous MeCN/THF (3:1).

#### Compound **47**:

16.6 mg (8.9%) white solid; mp 146.1–147.7 °C; [α]^25^_D_ −242.3 (*c* 0.21, CHCl_3_); HR-MS *m*/*z* 664.41981 [M + H]^+^ (calcd for C_40_H_58_NO_7_^+^ 664.42078); HPLC purity 98.0%.

### Host Cell Cytotoxicity Assessment

Cytotoxicity or antiproliferative effects of ecdysteroid against mammalian cells was assessed using MTT (3-(4,5-dimethylthiazol-2-yl)-2,5-diphenyltetrazolium bromide). CHO or RAW264.7 cells were used for screening the compounds. Briefly, the cells were seeded at a density of 2000/well in 96-well plates. After 24 h of incubation at 37 °C and 5% CO_2_, ecdysteroid (C44) was added to the cells at various concentrations. In all the experiments, podophyllotoxin was used as the positive control. In all experiments, vehicle controls were included, and the final DMSO concentration was below 0.5%. After 72 h, the plates were examined under an inverted microscope for sterility and growth of controls after the incubation period. The spent medium was removed, and 100 μL of fresh medium containing 0.5 mg/mL of the final concentration of MTT was added to the cells. The plates were incubated for 4 h at 37 °C in 5% CO_2_. The formazan crystals were solubilized by adding 200 μL of DMSO and mixed thoroughly. The absorbance was determined at 550 nm by using a Tecan plate reader. The values were corrected to the blank medium and vehicle control (DMSO only). Results were expressed as percentage cell viability relative to the vehicle control. Each assay was performed in technical triplicate in three independent experiments.

### Trypanocidal Activity

Trypanocidal activity against epimastigotes was assessed by XTT (2,3-bis(2-methoxy-4-nitro-5-sulfophenyl)-2*H*-tetrazolium-5-carboxanilide). Briefly, 1.5 × 10^6^ epimastigotes were seeded per well in 96-well plates. The ecdysteroid was added at 8 concentrations ranging from 25 to 0.2 μM for 72 h at 28 °C. In all of the experiments, benznidazole was used as the positive control. Following the incubation period, the plates were examined under the microscope for sterility and growth of controls. To the plates, 50 μL of XTT and PMS (phenazine methosulfate, Sigma-Aldrich, MO, USA) solution (XTT and PMS at 0.5 and 0.025 mg/mL, respectively) was added, and the plates were then incubated at 28 °C for 3 h. The parasites were fixed by the addition of 50 μL of MeOH for 15 min before the absorbance was measured at 490 nm on a Tecan plate reader. Results were expressed as percentage cell viability relative to the vehicle control or IC_50_ values calculated by GraphPad Prism version 8.0. All assessments were performed in triplicates in three independent experiments.

### FACS-Based Quantitation of Released Parasites from Infected Cells

The host RAW264.7 cells were seeded in 24-well plates at a density of 20,000 cells/mL. The cells were allowed to adhere for 24 h and infected with trypomastigotes at a multiplicity of 10. On the next day, noninternalized trypomastigotes were washed away, and fresh RPMI with 2% hiFBS was added to the wells along with the C44 at various concentrations. All experiments included noninfected control, DMSO control, and benznidazole control at 20 μM. Three independent experiments in triplicates were performed to determine the activity of the ecdysteroid.

### Microscopy

The host CCL39 cells were seeded in 24-well plates at a density of 20,000 cells/mL. The cells were allowed to adhere for 24 h and infected with trypomastigotes at a multiplicity of 10. On the next day, non-internalized trypomastigotes were washed away, and fresh RPMI with 2% hiFBS was added to the wells along with the test substance. On the fourth day after infection, the cells were fixed with 4% paraformaldehyde for 1 h and scanned on a epi-fluorescence Nikon eclipse TS2 microscope. The images were analyzed on ImageJ.

## Data Availability

The raw NMR spectra for compounds 38-47 are freely available on Zenodo with DOI: 10.5281/zenodo.12721383

## Supporting Information Available

Characteristic 1D and 2D NMR spectra, HR-MS spectra, and HPLC purity data for compounds **38**–**47** (Figures S1–S75); antitrypanosomal screening results (Figure S76) (PDF)
