# Supplementary material for: Semisynthetic Ecdysteroid Cinnamate Esters and tert-Butyl Oxime Ether Derivatives with Trypanocidal Activity
Source: J Nat Prod. 2024 Oct 17;87(10):2478–86. doi: 10.1021/acs.jnatprod.4c00811 (PMC11519910; doi:10.1021/acs.jnatprod.4c00811)
Supplement: Supplementary file 1 — np4c00811_si_001.pdf [file np4c00811_si_001.pdf]

SUPPORTING INFORMATION FOR

Semisynthetic Ecdysteroid Cinnamate Esters and *tert*-Butyl Oxime Ether Derivatives with Trypanocidal Activity

Márton B. Háznagy[a], Gábor Girst[a], Máté Vágvölgyi[a], Kaushavi Cholke[b] Sandhya Radha Krishnan[b], Jürg Gertsch[b], Attila Hunyadi[a, c,d,e,*]

[a] Institute of Pharmacognosy, University of Szeged, Eötvös u. 6, H-6720 Szeged, Hungary

[b] Institute of Biochemistry and Molecular Medicine, University of Bern, 3012 Bern, Switzerland

[c] Interdisciplinary Centre of Natural Products, University of Szeged, Eötvös u. 6, H-6720 Szeged, Hungary

[d] HUN-REN-SZTE Biologically Active Natural Products Research Group, Eötvös u. 6, H-6720 Szeged, Hungary

[e] Graduate Institute of Natural Products, Kaohsiung Medical University, Shih-Chuan 1st Rd. 100, Kaohsiung 807, Taiwan

Table of Contents

Figure S1. ^{1}H NMR (500 MHz, acetone-d_6) spectrum of compound **38** 5
Figure S2. JMOD NMR (126 MHz, acetone-d_6) spectrum of compound **38** 6
Figure S3. HSQC NMR (acetone-d_6) spectrum of compound **38**........ 7
Figure S4. ^{1}H-^{1}H COSY NMR (acetone-d_6) spectrum of compound **38**........ 8
Figure S5. HMBC NMR (acetone-d_6) spectrum of compound **38** 9
Figure S6. HR-MS spectra of compound **38** 10
Figure S7. HPLC chromatogram of compound **38**........ 11
Figure S8. ^{1}H NMR (500 MHz, acetone-d_6) of compound **39** 12
Figure S9. JMOD NMR (126 MHz, acetone-d_6) spectrum of compound **39** 13
Figure S10. HSQC NMR (acetone-d_6) spectrum of compound **39**........ 14
Figure S11. ^{1}H-^{1}H COSY NMR (acetone-d_6) spectrum of compound **39** 15
Figure S12. HMBC NMR (acetone-d_6) spectrum of compound **39**........ 16
Figure S13. HR-MS spectra of compound **39** 17
Figure S14. HPLC chromatogram of compound **39**........ 18
Figure S15. ^{1}H NMR (500 MHz, acetone-d_6) spectrum of compound **40**........ 19
Figure S16. JMOD NMR (126 MHz, acetone-d_6) spectrum of compound **40** 20
Figure S17: HSQC NMR (acetone-d_6) spectrum of compound **40** 21
Figure S18. ^{1}H-^{1}H COSY NMR (acetone-d_6) spectrum of compound **40**........ 22
Figure S19. HMBC NMR (acetone-d_6) spectrum of compound **40** 23
Figure S20. HR-MS spectrum of compound **40** 24
Figure S21. HPLC chromatogram of Compound **40**........ 24
Figure S22. ^{1}H NMR (500 MHz, acetone-d_6) spectrum of compound **41**........ 25
Figure S23. JMOD NMR (126 MHz, acetone-d_6) spectrum of compound **41** 26
Figure S24. HSQC NMR (acetone-d_6) spectrum of compound **41**........ 27
Figure S25. ^{1}H-^{1}H COSY NMR (acetone-d_6) spectrum of compound **41**........ 28
Figure S26. HMBC NMR (acetone-d_6) spectrum of compound **41** 29
Figure S27. HR-MS spectrum of compound **41** 30
Figure S28. ^{1}H NMR (500 MHz, DMSO-d_6) of compound **42** 31
Figure S29. JMOD NMR (126 MHz, DMSO-d_6) of compound **42** 32
Figure S30. HSQC NMR (DMSO-d_6) spectrum of compound **42** 33
Figure S31. ^{1}H-^{1}H COSY NMR (DMSO-d_6) spectrum of compound **42** 34
Figure S32. HMBC NMR (DMSO-d_6) spectrum of compound **42**........ 35
Figure S33. ROESY (DMSO-d_6) NMR spectrum of compound **42**........ 36
Figure S34. HR-MS spectrum of compound **42** 37

Figure S35. HPLC chromatogram of compound **42** 37
Figure S36. ^{1}H NMR (500 MHz, DMSO-d_6) spectrum of compound **43** 38
Figure S37. JMOD NMR (126 MHz, DMSO-d_6) spectrum of compound **43** 39
Figure S38. HSQC NMR (DMSO-d_6) spectrum of compound **43** 40
Figure S39. ^{1}H-^{1}H COSY NMR (DMSO-d_6) spectrum of compound **43** 41
Figure S40. HMBC NMR (DMSO-d_6) spectrum of compound **43** 42
Figure S41. ROESY NMR (DMSO-d_6) spectrum of compound **43** 43
Figure S42. HR-MS spectrum of compound **43** 44
Figure S43. HPLC chromatogram of compound **43** 44
Figure S44. ^{1}H NMR (500 MHz, acetone-d_6) of compound **44** 45
Figure S45. JMOD NMR (126 MHz, acetone-d_6) spectrum of compound **44** 46
Figure S46. HSQC NMR (acetone-d_6) spectrum of compound **44** 47
Figure S47. ^{1}H-^{1}H COSY NMR (acetone-d_6) spectrum of compound **44** 48
Figure S48. HMBC NMR (acetone-d_6) spectrum of compound **44** 49
Figure S49. ROESY NMR (acetone-d_6) spectrum of compound **44** 50
Figure S50. HR-MS spectrum of compound **44** 51
Figure S51. HPLC chromatogram of compound **44** 51
Figure S52. ^{1}H NMR (500 MHz, acetone-d_6) spectrum of compound **45** 52
Figure S53. JMOD NMR (126 MHz, acetone-d_6) spectrum of compound **45** 53
Figure S54. HSQC NMR (acetone-d_6) spectrum of compound **45** 54
Figure S55. ^{1}H-^{1}H COSY NMR (acetone-d_6) spectrum of compound **45** 55
Figure S56. HMBC NMR (acetone-d_6) spectrum of compound **45** 56
Figure S57. ROESY NMR (acetone-d_6) spectrum of compound **45** 57
Figure S58. HR-MS spectrum of compound **45** 58
Figure S59. HPLC chromatogram of compound **45** 58
Figure S60. ^{1}H NMR (500 MHz, acetone-d_6) of compound **46** 59
Figure S61. JMOD NMR (126 MHz, acetone-d_6) spectrum of compound **46** 60
Figure S62. HSQC NMR (acetone-d_6) spectrum of compound **46** 61
Figure S63. ^{1}H-^{1}H COSY NMR (acetone-d_6) spectrum of compound **46** 62
Figure S64. HMBC NMR (acetone-d_6) spectrum of compound **46** 63
Figure S65. ROESY NMR (acetone-d_6) spectrum of compound **46** 64
Figure S66. HR-MS spectrum of compound **46** 65
Figure S67. HPLC chromatogram of compound **46** 65
Figure S68. ^{1}H NMR (500 MHz, acetone-d_6) spectrum of compound **47** 66
Figure S69. JMOD NMR (126 MHz, acetone-d_6) spectrum of compound **47** 67

Figure S70. HSQC NMR (acetone-d_6) spectrum of compound **47**......68

Figure S71. HMBC NMR (acetone-d_6) spectrum of compound **47** 69

Figure S72. ^{1}H-^{1}H COSY NMR (acetone-d_6) spectrum of compound **47**......70

Figure S73. ROESY NMR (acetone-d_6) spectrum of compound **47** 71

Figure S74. HR-MS spectrum of compound **47** 72

Figure S75. HPLC chromatogram of compound **47** 72

Figure S76. Screening of ecdysteroids for selective trypanocidal effects in CHO cells versus *T. cruzi* epimastigotes at 5 μM. 73

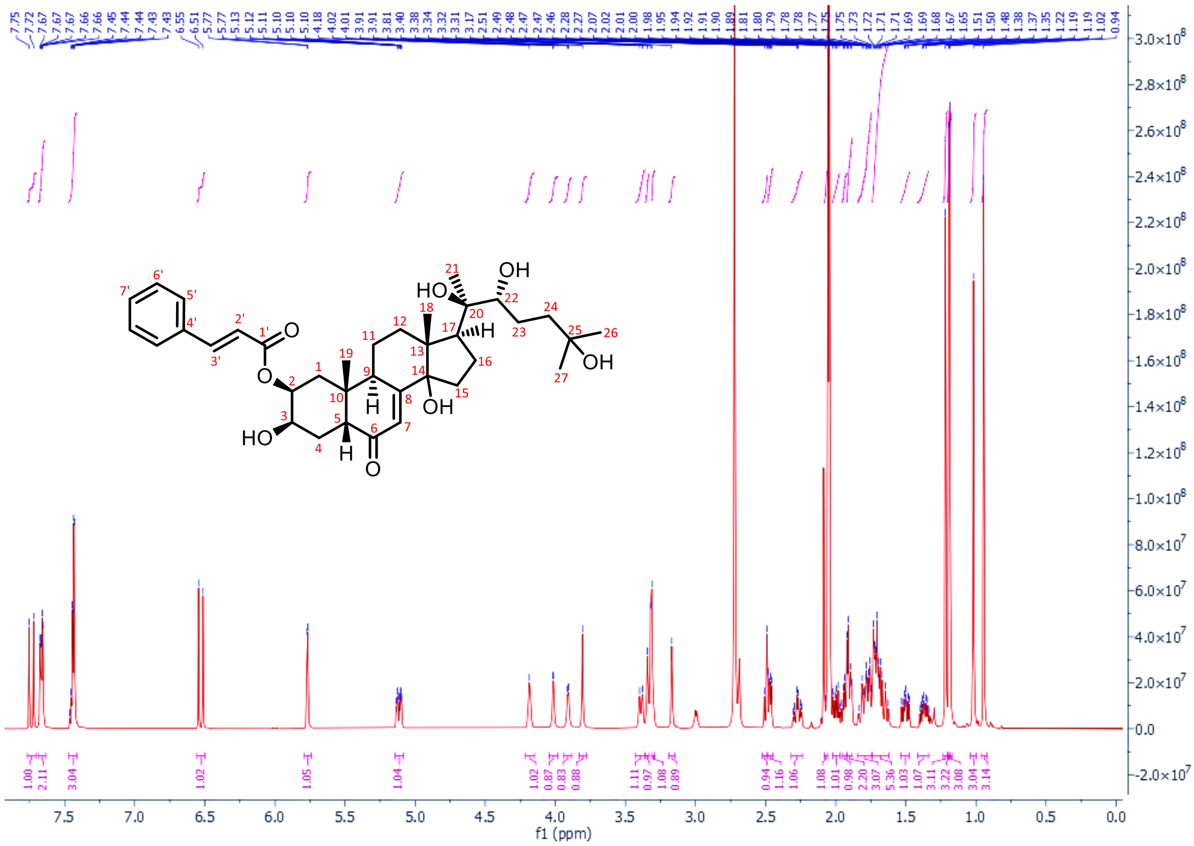

Figure S1. ^{1}H NMR (500 MHz, acetone-d_6) spectrum of compound **38**
20-hydroxyecdysone-2-cinnamate

^{1}H NMR (500 MHz, acetone-d_6) δ 7.74 (d, J = 16.1 Hz, 1H, H-3′), 7.69 – 7.64 (m, 2H, H-5′), 7.47 – 7.41 (m, 3H, H-6′ and H-7′), 6.53 (d, J = 16.1 Hz, 1H, H-2′), 5.77 (d, J = 2.4 Hz, 1H, H-7), 5.14 – 5.08 (m, 1H, H-2), 4.18 (br s, 1H, H-3), 4.02 (br d, 1H, 3-OH), 3.91 (br d, J = 3.5 Hz, 1H, 22-OH), 3.81 (s, 1H, 14-OH), 3.39 (br d, J = 10.3 Hz, 1H, H-22), 3.34 (s, 1H, 25-OH), 3.31 – 3.29 (m, 1H, 9-H), 3.17 (s, 1H, 20-OH), 2.49 (dd, J = 9.4, 8.7 Hz, 1H, H-17), 2.49 – 2.45 (m, 1H, H-5), 2.27 (td, J = 12.9, 4.8 Hz, 1H, H-12), 2.08 – 2.06 (m, 1H, H-16), 2.02 – 1.97 (m, 1H, H-15), 1.96 – 1.92 (m, 1H, H-1), 1.92 – 1.88 (m, 2H, H-11 and H-12), 1.85 – 1.74 (m, 3H, H-4, H-11 and H-24), 1.74 – 1.62 (m, 5H, H-1, H-4, H-15, H-16 and H-23), 1.54 – 1.47 (m, 1H, H-24), 1.42 – 1.34 (m, 1H, H-23), 1.22 (s, 3H, H-21), 1.19 (s, 3H, H-26), 1.19 (s, 3H, H-27), 1.02 (s, 3H, H-19), 0.94 (s, 3H, H-18).

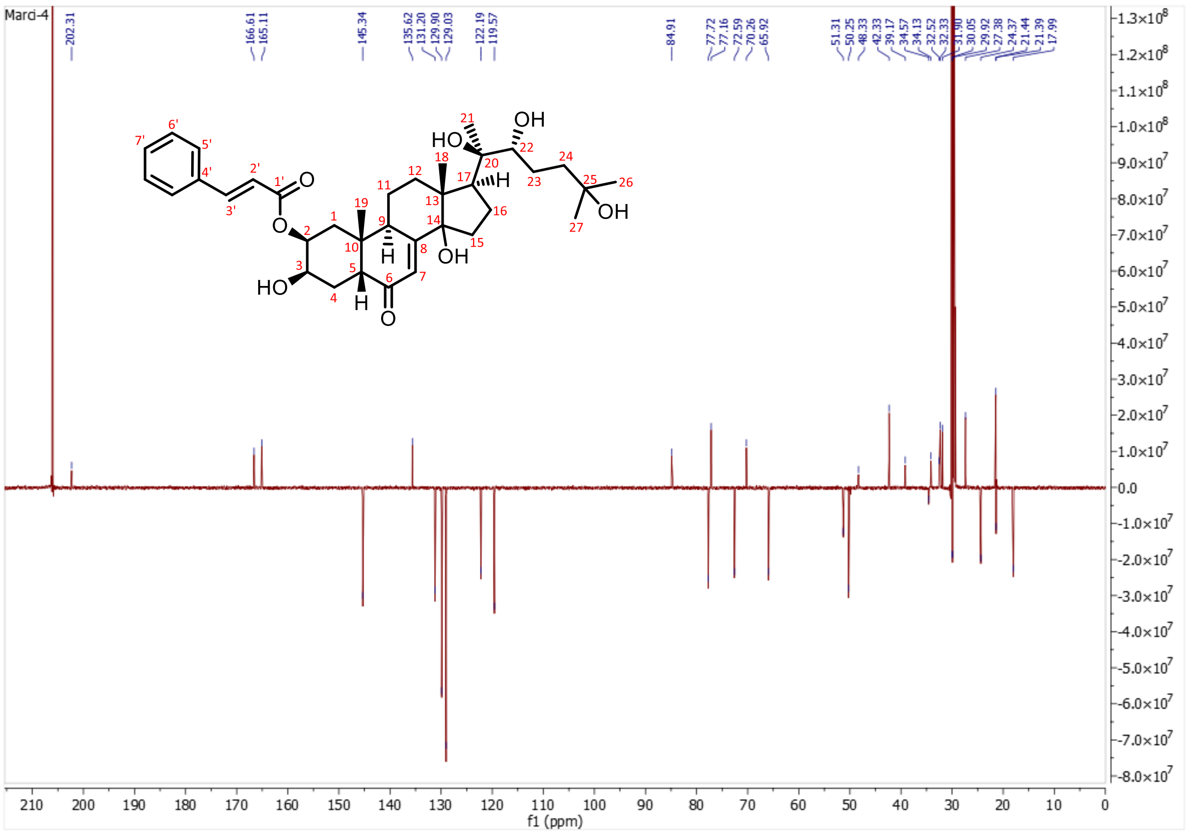

Figure S2. JMOD NMR (126 MHz, acetone-d_6) spectrum of compound **38**
20-hydroxyecdysone-2-cinnamate

^{13}C NMR (126 MHz, acetone-d_6) δ 202.3 (C-6), 166.6 (C-1′), 165.1 (C-8), 145.3 (C-3′), 135.6 (C-4′), 131.2 (C-7′), 129.9 (C-6′), 129.0 (C-5′), 122.2 (C-7), 119.6 (C-2′), 84.9 (C-14), 77.7 (C-22), 77.2 (C-20), 72.6 (C-2), 70.3 (C-25), 65.9 (C-3), 51.3 (C-5), 50.3 (C-17), 48.3 (C-13), 42.3 (C-24), 39.2 (C-10), 34.6 (C-9), 34.1 (C-1), 32.5 (C-4), 32.3 (C-12), 31.9 (C-15), 30.1 (C-27), 29.9 (C-26), 27.4 (C-23), 24.4 (C-19), 21.44 (C-11 and C-16), 21.39 (C-21), 18.0 (C-18).

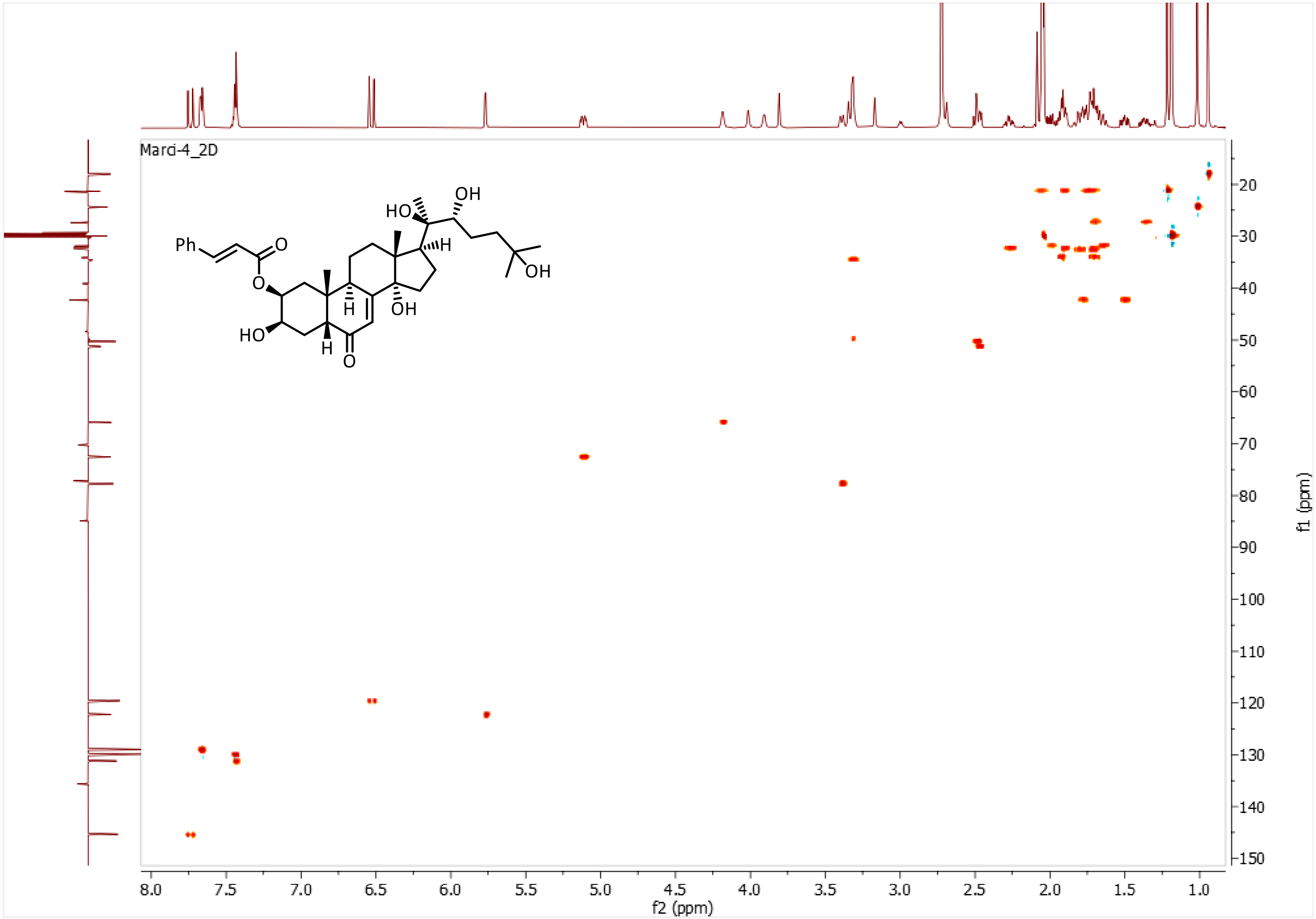

Figure S3. HSQC NMR (acetone-d_6) spectrum of compound **38** (20-hydroxyecdysone 2-cinnamate)

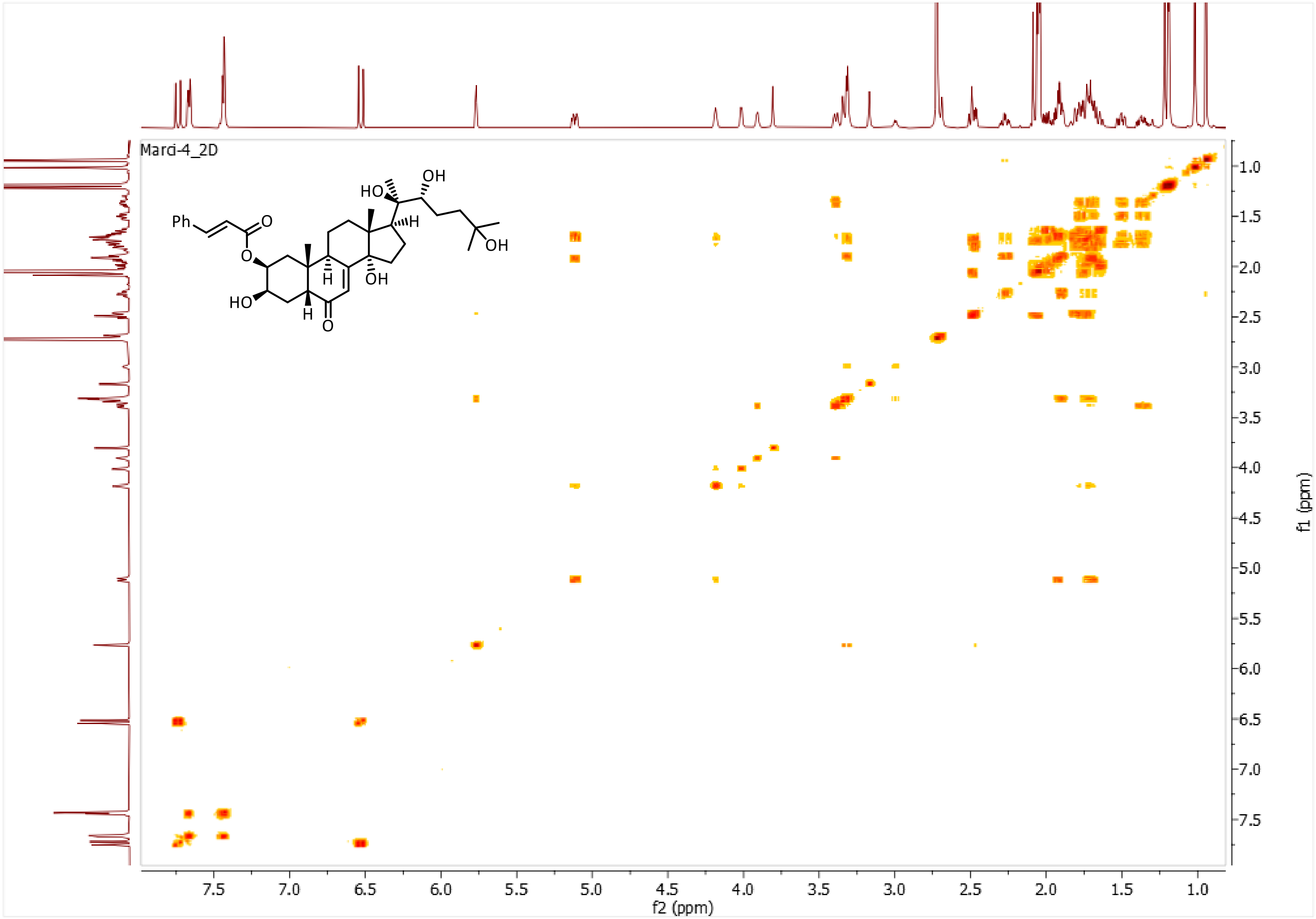

Figure S4. ^{1}H-^{1}H COSY NMR (acetone-d_6) spectrum of compound **38** (20-hydroxyecdysone 2-cinnamate)

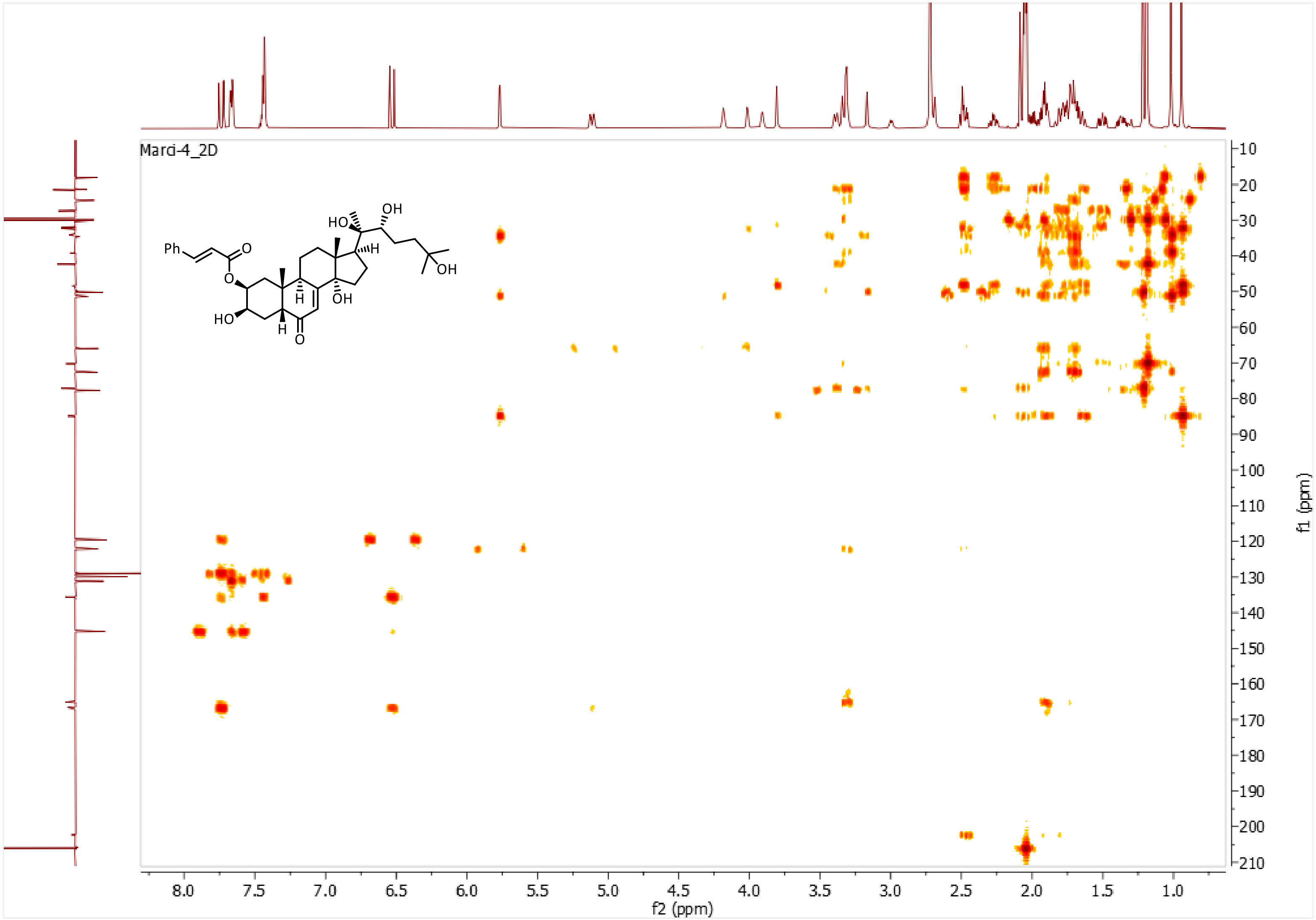

Figure S5. HMBC NMR (acetone-d_6) spectrum of compound **38** (20-hydroxyecdysone 2-cinnamate)

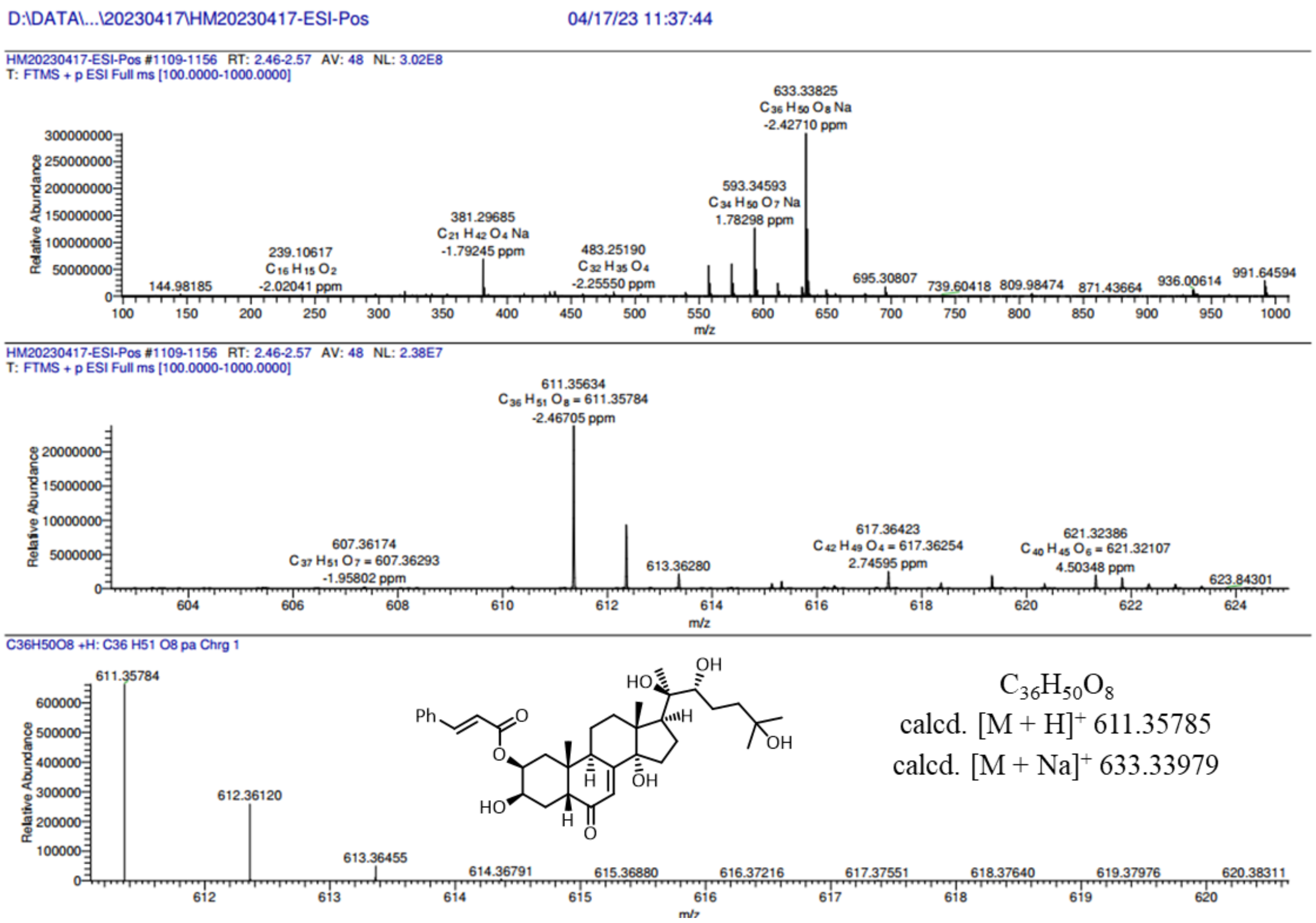

Figure S6. HR-MS spectra of compound **38**

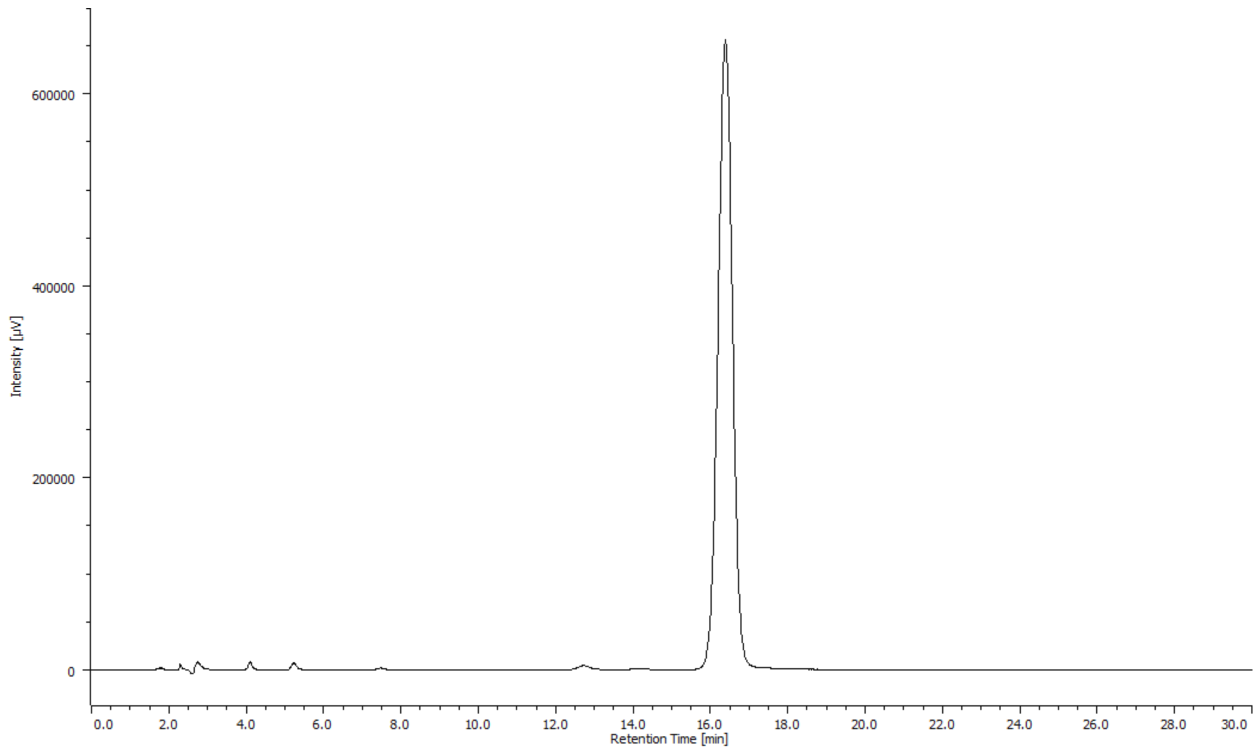

Figure S7. HPLC chromatogram of compound **38** at its UV absorbance maximum (λ=279.3 nm). Purity 98.1 %. Column: Kinetex®, 5 µm, Biphenyl 100 Å, 250 × 4.6 mm (Phenomenex Inc.); Elution H_2O:CH_3CN (A:B), 35 % B

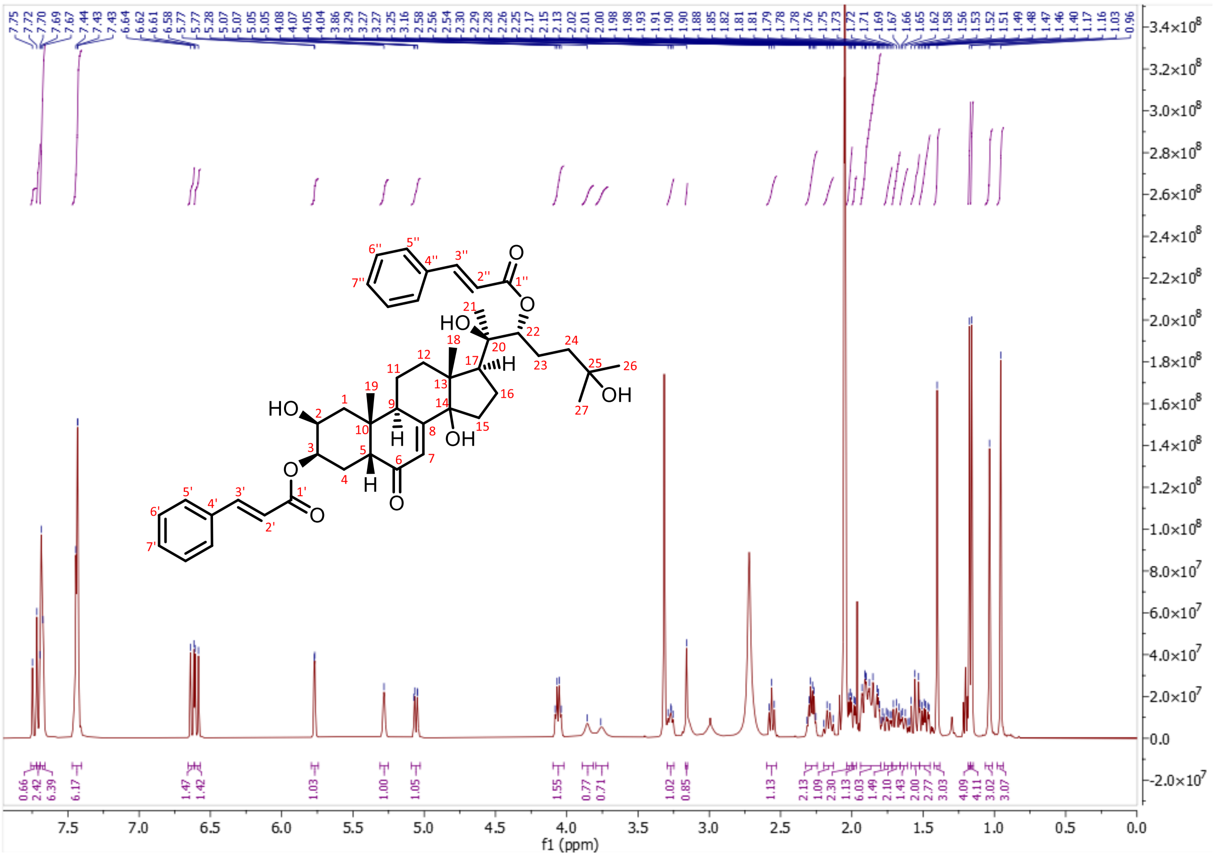

Figure S8. ^{1}H NMR (500 MHz, acetone-d_6) of compound **39**
20-hydroxyecdysone 3,22-dicinnamate

^{1}H NMR (500 MHz, acetone-d_6) δ 7.73 (d, J = 16.0 Hz, 1H, H-3′), 7.71 (d, J = 16.0 Hz, 1H, H-3″), 7.69 – 7.66 (m, 4H, H-5′ and 5″), 7.47 – 7.40 (m, 6H, H-6′, H-7′, H-6″ and H-7″), 6.62 (d, J = 16.0 Hz, 1H, H-2′), 6.60 (d, J = 16.0 Hz, 1H, H-2″), 5.77 (br d, J = 1.7 Hz, 1H, H-7), 5.28 (br s, 1H, H-3), 5.06 (dd, J = 10.7, 1.8 Hz, 1H, H-22), 4.10 – 4.02 (m, 1H, H-2), 3.86 (br s, 1H, 14-OH), 3.76 (br s, 1H, 2-OH), 3.30 – 3.25 (m, 1H, H-9), 3.16 (br s, 1H, 20-OH), 2.56 (t, J = 9.2 Hz, 1H, H-17), 2.32 – 2.24 (m, 2H, H-5 and H-12), 2.20 – 2.13 (m, 1H, H-16), 2.04 – 2.00 (m, 1H, H-15), 2.00 – 1.97 (m, 1H, H-1), 1.94 – 1.80 (m, 6H, H-4, H-11, H-12, H-16 and H-23), 1.77 – 1.72 (m, 1H, H-11), 1.72 – 1.66 (m, 1H, H-15), 1.66 – 1.61 (m, 1H, H-23), 1.58 – 1.53 (m, 1H, H-1), 1.53 – 1.45 (m, 2H, H-24), 1.40 (s, 3H, H-21), 1.17 (s, 3H, H-27), 1.16 (s, 3H, H-26), 1.03 (s, 3H, H-19), 0.96 (s, 3H, H-18).

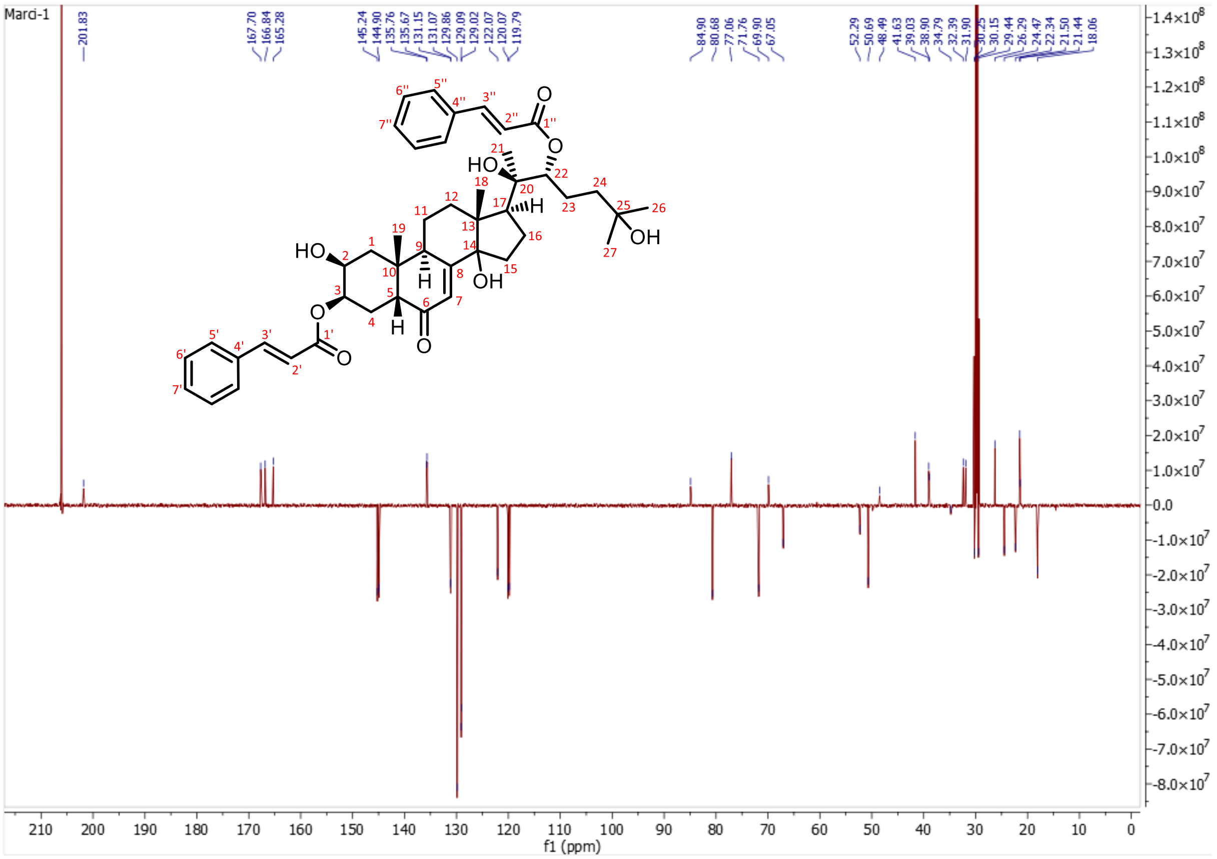

Figure S9. JMOD NMR (126 MHz, acetone-d_6) spectrum of compound **39**
20-hydroxyecdysone 3,22-dicinnamate

^{13}C NMR (126 MHz, acetone-d_6) δ 201.8 (C-6), 167.7 (C-1″), 166.8 (C-1′), 165.3 (C-8), 145.2 (C-3′), 144.9 (C-3″), 135.8 (C-4″), 135.7 (C-4′), 131.1 (C-7′), 131.1(C-7″), 129.9 (C-6′, C-6″), 129.1 (C-5″), 129.0 (C-5′), 122.1 (C-7), 120.1(C-2″), 119.8 (C-2′), 84.9 (C-14), 80.7 (C-22), 77.1 (C-20), 71.8 (C-3), 69.9 (C-25), 67.1 (C-2), 52.3 (C-5), 50.7 (C-17), 48.5 (C-13), 41.6 (C-24), 39.0 (C-1), 38.9 (C-10), 34.8 (C-9), 32.4 (C-12), 31.9 (C-15), 30.3 (C-27), 30.1 (C-4), 29.4 (C-26), 26.3 (C-23), 24.5 (C-19), 22.3 (C-21), 21.5 (C-16), 21.4 (C-11), 18.1 (C-18).

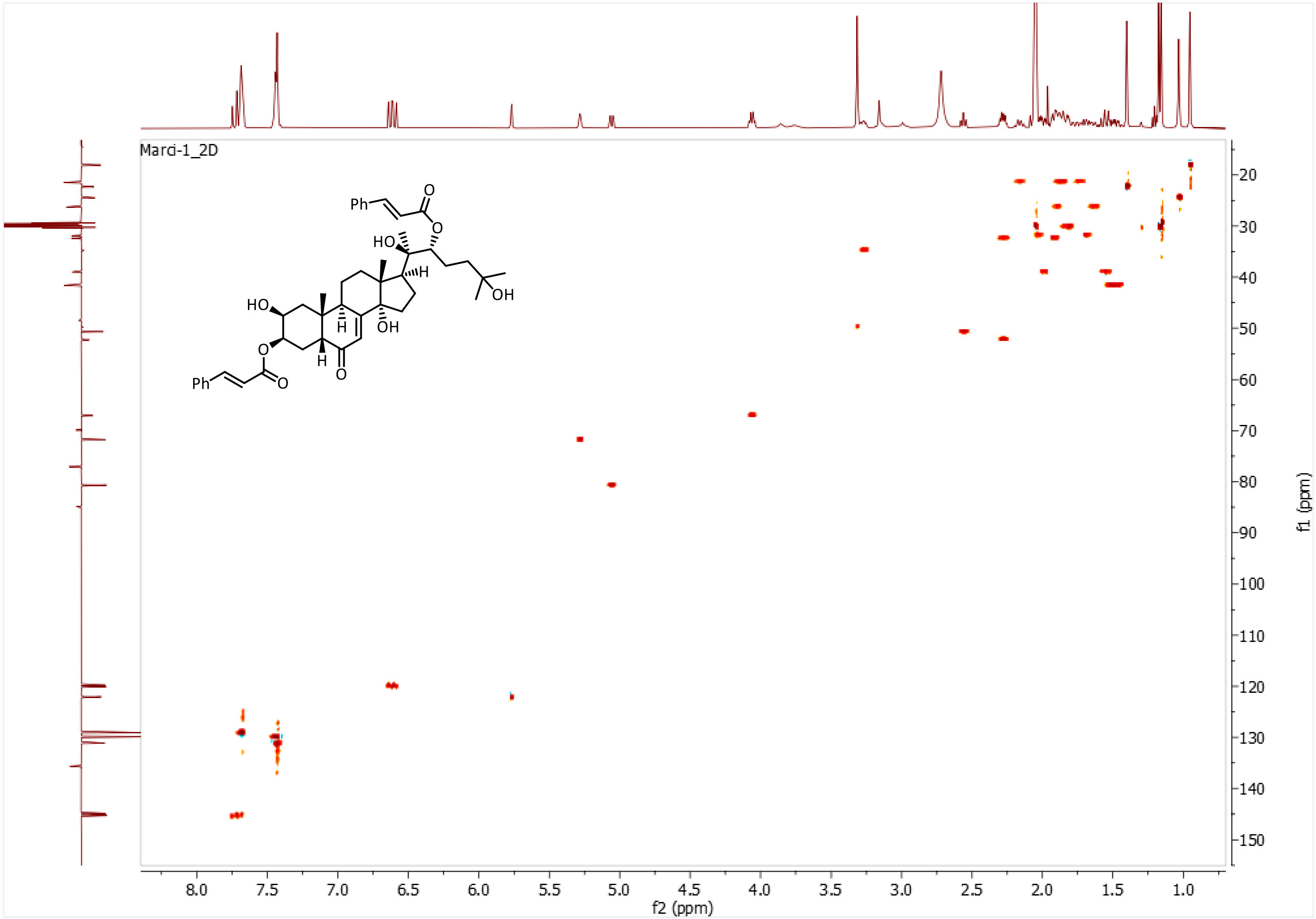

Figure S10. HSQC NMR (acetone-d_6) spectrum of compound **39**
20-hydroxyecdysone 3,22-dicinnamate

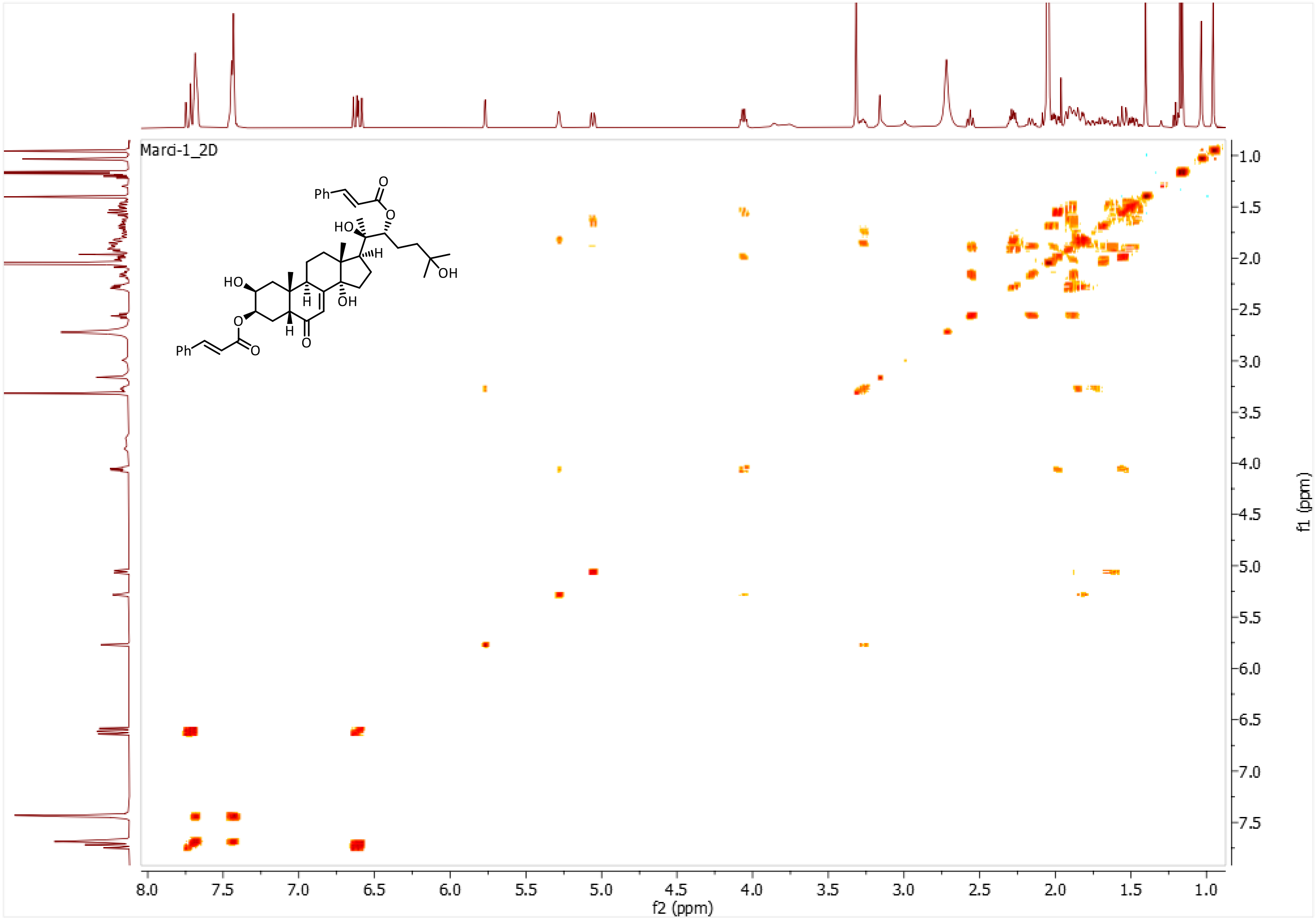

Figure S11. ^{1}H-^{1}H COSY NMR (acetone-d_6) spectrum of compound **39**
20-hydroxyecdysone 3,22-dicinnamate

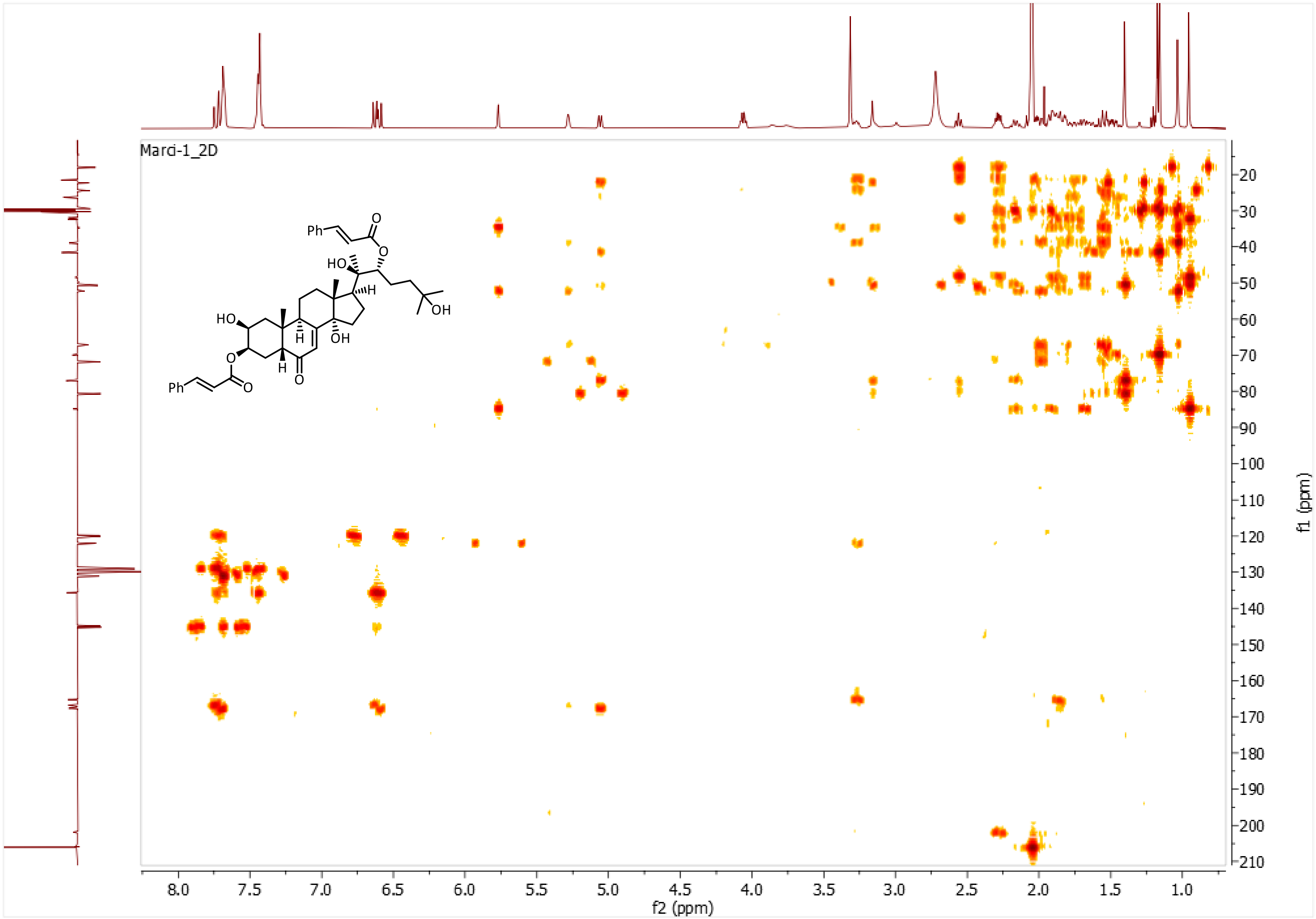

Figure S12. HMBC NMR (acetone-d_6) spectrum of compound **39**
20-hydroxyecdysone 3,22-dicinnamate

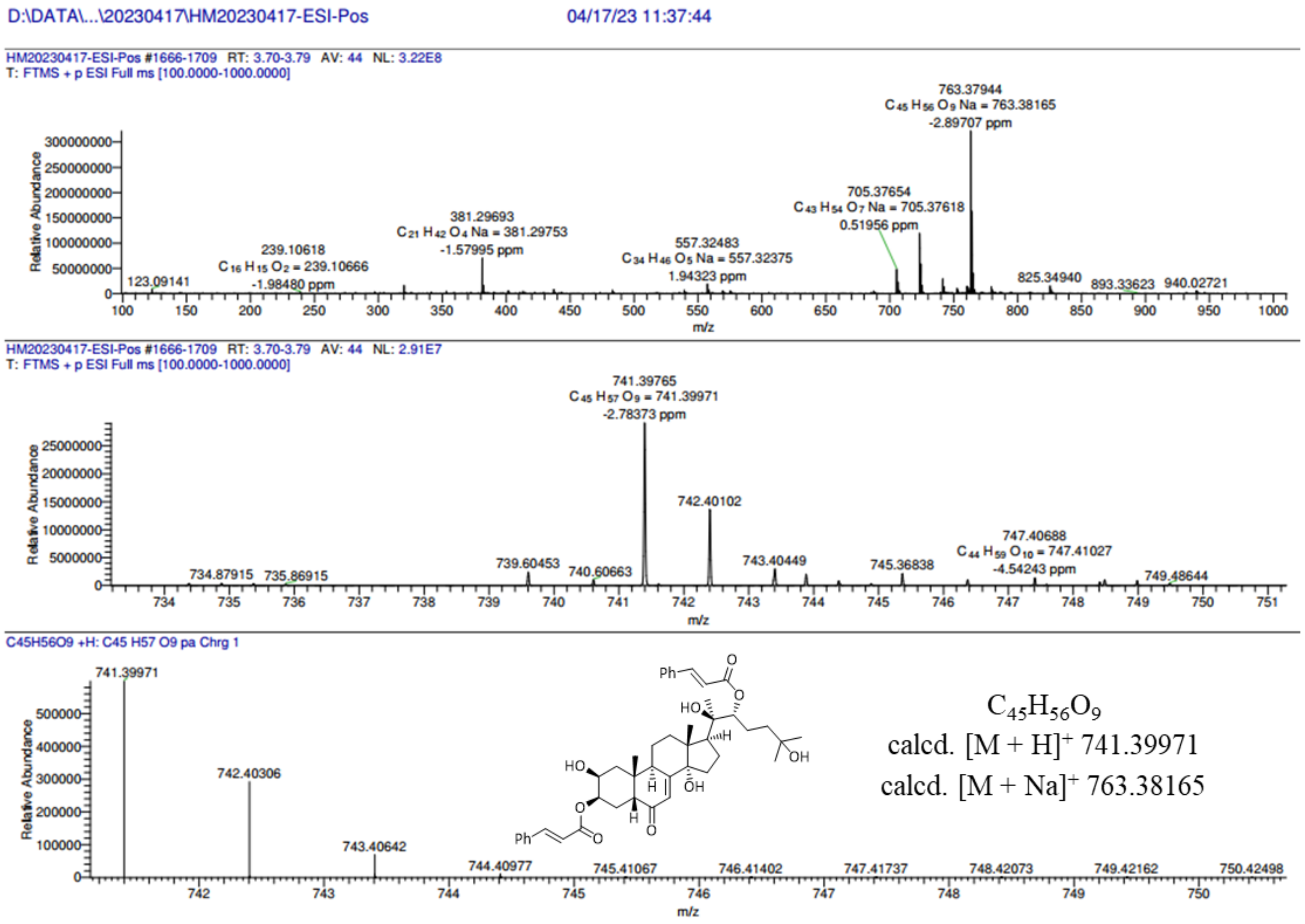

Figure S13. HR-MS spectra of compound **39**

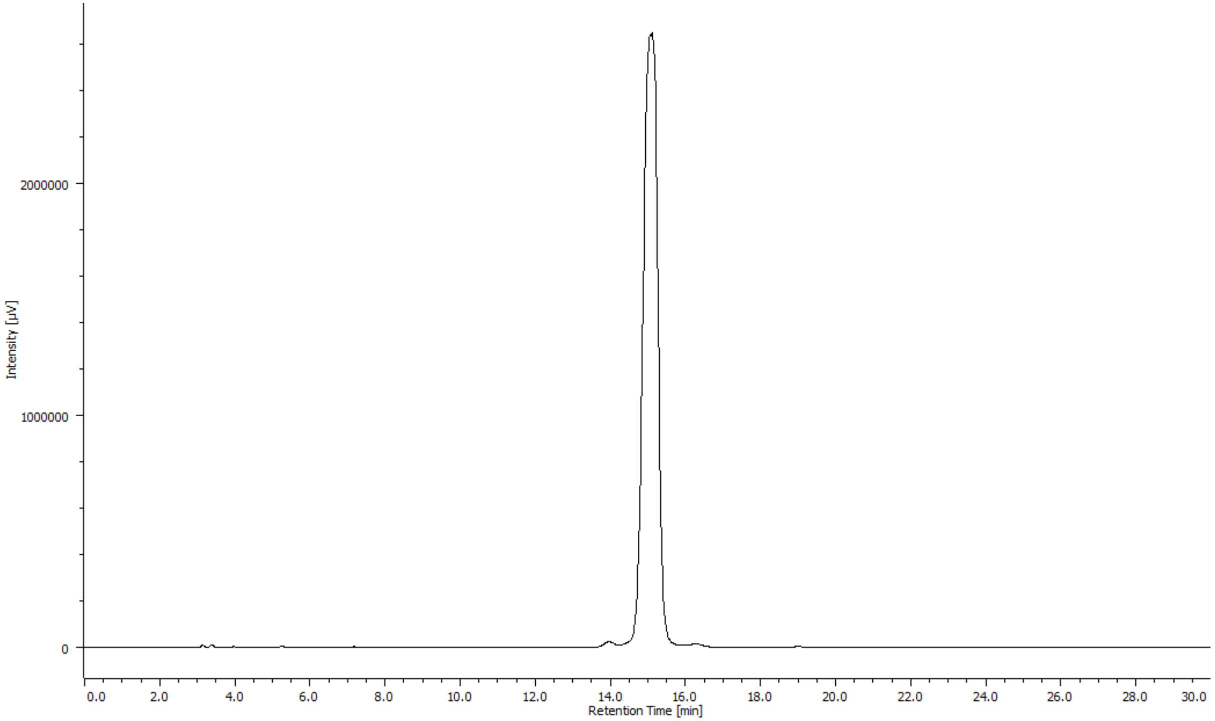

Figure S14. HPLC chromatogram of compound **39** at its UV absorbance maximum(λ=271.0 nm). Purity 98.2 %. Column: Kinetex®, 5 µm, Biphenyl 100 Å, 250 × 4.6mm, (Phenomenex Inc.); Elution: H_2O:CH_3CN (A:B), 52 % B

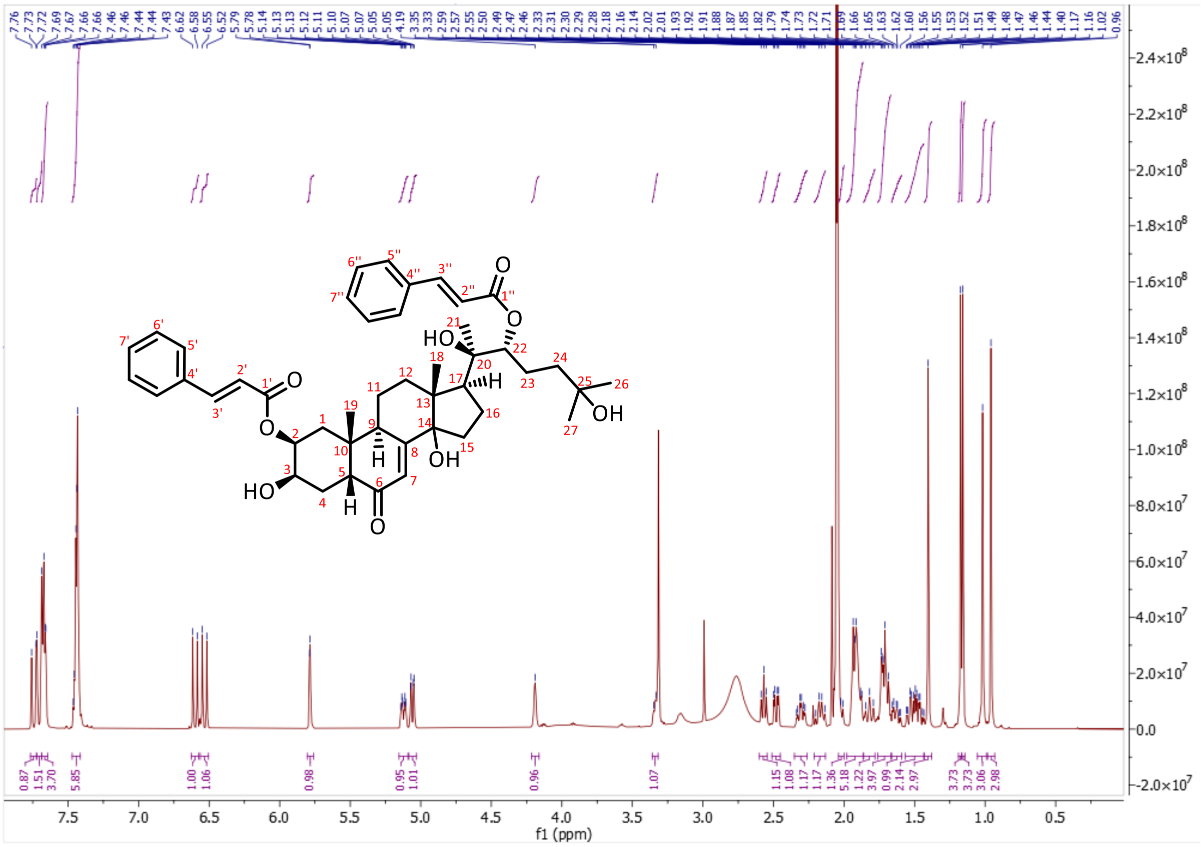

Figure S15. ^{1}H NMR (500 MHz, acetone-d_6) spectrum of compound **40**
20-hydroxyecdysone 2,22-dicinnamate

^{1}H NMR (500 MHz, acetone-d_6) δ 7.74 (d, J = 16.1 Hz, 1H, H-3′), 7.70 (d, J = 16.3 Hz, 1H, H-3″), 7.69 – 7.64 (m, 4H, H-5′ and H-5″), 7.47 – 7.41 (m, 6H, H-6′, H-7′, H-6″ and H-7″), 6.60 (d, J = 16.0 Hz, 1H, H-2″), 6.53 (d, J = 16.1 Hz, 1H, H-2′), 5.79 (d, J = 2.1 Hz, 1H, H-7), 5.12 (m, 1H, H-2), 5.06 (dd, J = 10.5, 2.0 Hz, 1H, H-22), 4.19 (br s, 1H, H-3), 3.36 – 3.32 (m, 1H, H-9), 2.57 (t, J = 9.2 Hz, 1H, H-17), 2.48 (dd, J = 13.1, 4.0 Hz, 1H, H-5), 2.31 (td, J = 12.6, 4.3 Hz, 1H, H-12), 2.21 – 2.13 (m, 1H, H-16), 2.04 – 2.00 (m, 1H, H-15), 1.98 – 1.87 (m, 5H, H-1, H-11, H-12, H-16 and H-23), 1.86 – 1.78 (m, 1H, H-4), 1.76 – 1.67 (m, 4H, H-1, H-4, H-11 and H-15), 1.66 – 1.59 (m, 1H, H-23), 1.57 – 1.43 (m, 2H, H-24), 1.40 (s, 3H, H-21), 1.17 (s, 3H, H-27), 1.16 (s, 3H, H-26), 1.02 (s, 3H, H-19), 0.96 (s, 3H, H-18).

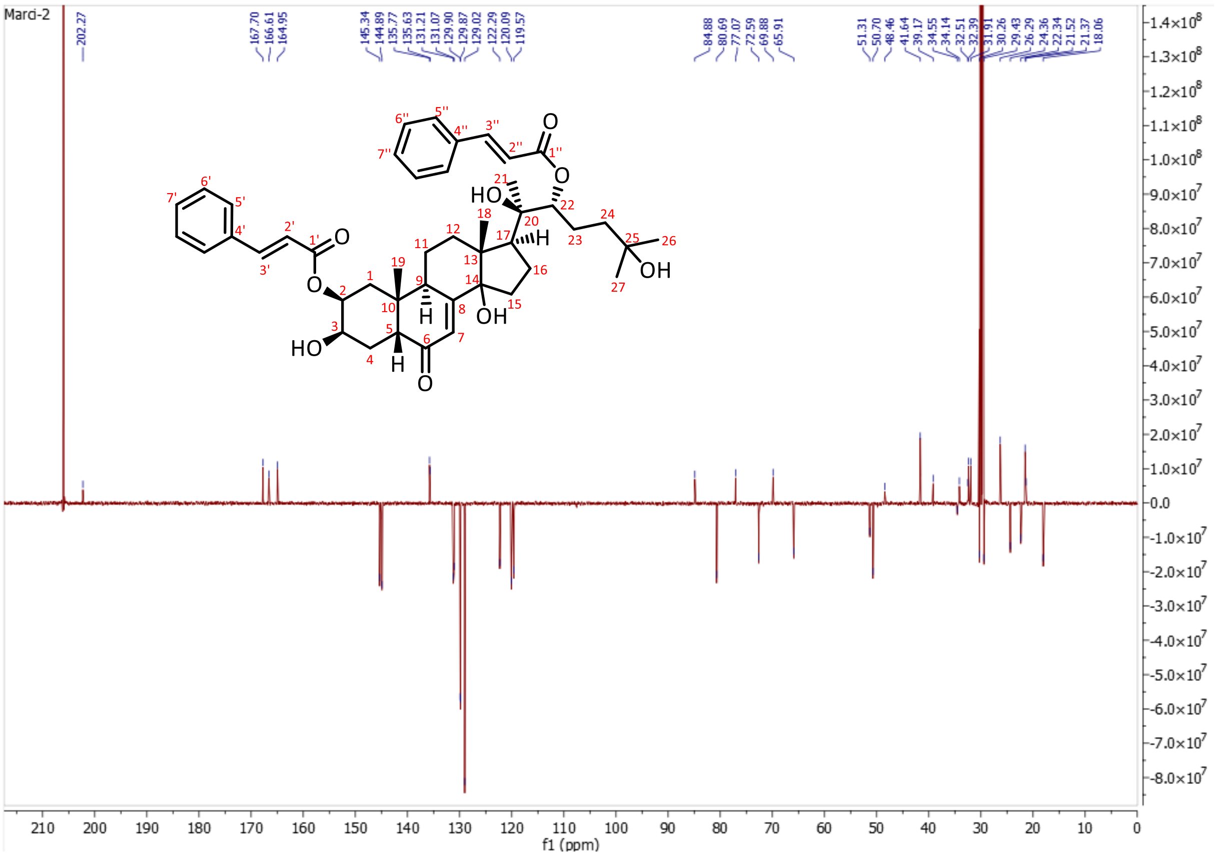

Figure S16. JMOD NMR (126 MHz, acetone-d_6) spectrum of compound **40**
20-hydroxyecdysone 2,22-dicinnamate

^{13}C NMR (126 MHz, acetone-d_6) δ 202.3 (C-6), 167.7 (C-1″), 166.6 (C-1′), 164.9 (C-8), 145.3 (C-3′), 144.9 (C-3″), 135.8 (C-4′), 135.6 (C-4″), 131.2 (C-7′), 131.1 (C-7″), 129.9 (C-6″), 129.9 (C-6′), 129.0 (C-5′), 122.3 (C-7), 120.1 (C-2″), 119.6 (C-2′), 84.9 (C-14), 80.7 (C-22), 77.1 (C-20), 72.6 (C-2), 69.9 (C-25), 65.9 (C-3), 51.3 (C-5), 50.7 (C-17), 48.5 (C-13), 41.6 (C-24), 39.2 (C-10), 34.5 (C-9), 34.1 (C-1), 32.5 (C-4), 32.4 (C-12), 31.9 (C-15), 30.3 (C-27), 29.4 (C-26), 26.3 (C-23), 24.4 (C-19), 22.3 (C-21), 21.5 (C-16), 21.4 (C-11), 18.1 (C-18).

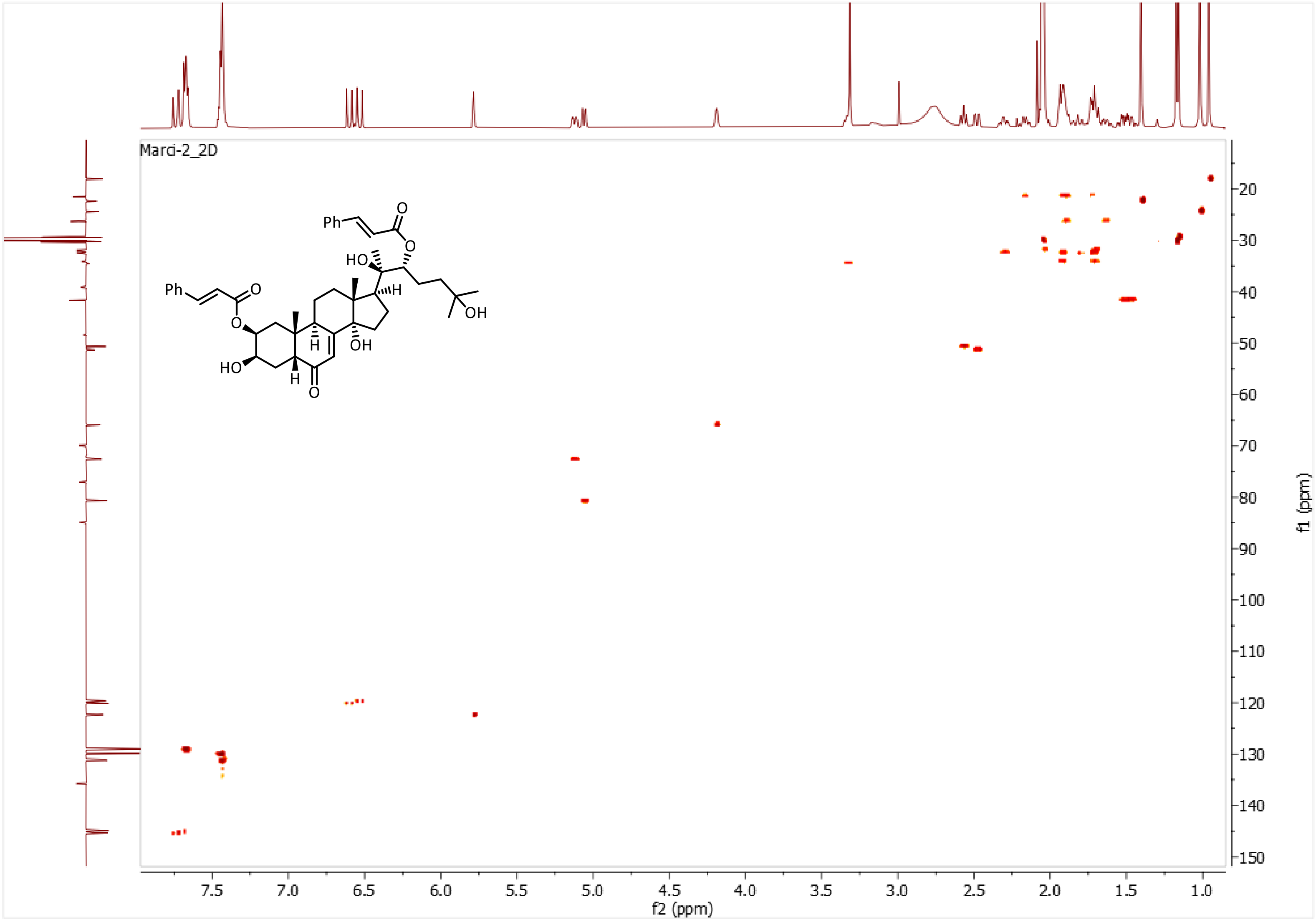

Figure S17: HSQC NMR (acetone-d_6) spectrum of compound **40** (20-hydroxyecdysone 2,22-dicinnamate)

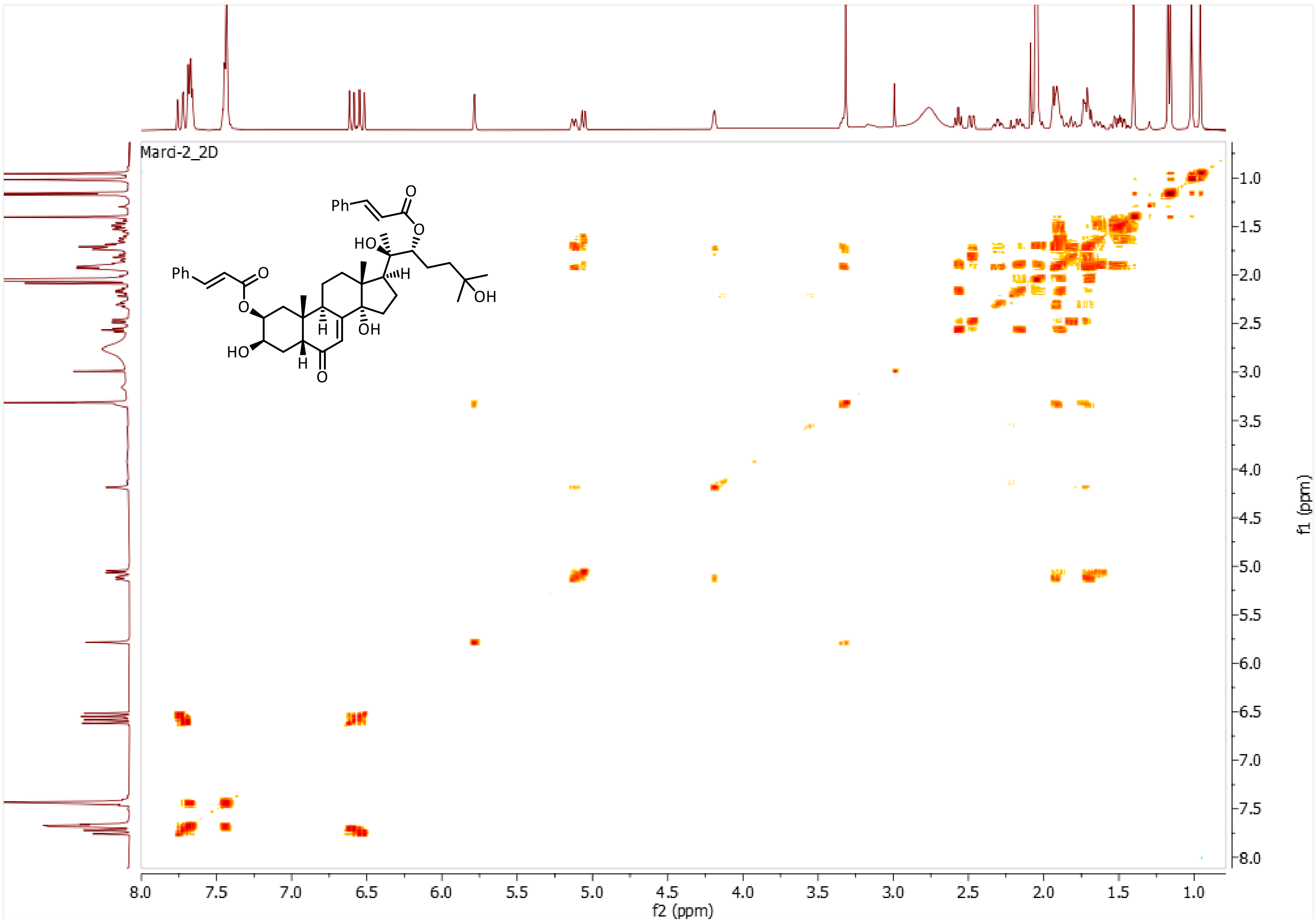

Figure S18. ^{1}H-^{1}H COSY NMR (acetone-d_6) spectrum of compound **40** (20-hydroxyecdysone 2,22-dicinnamate)

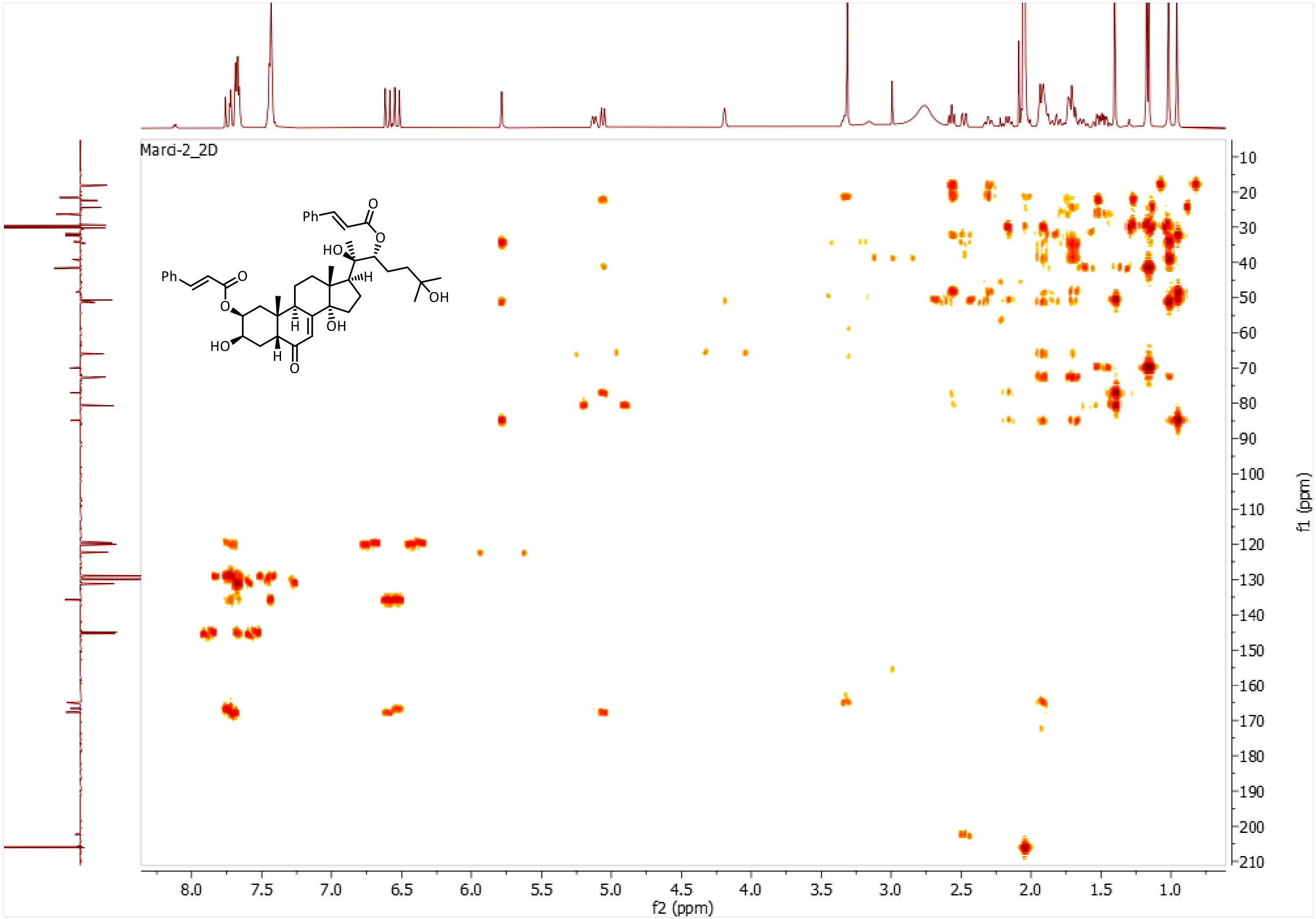

Figure S19. HMBC NMR (acetone-d_6) spectrum of compound **40** (20-hydroxyecdysone 2,22-dicinnamate)

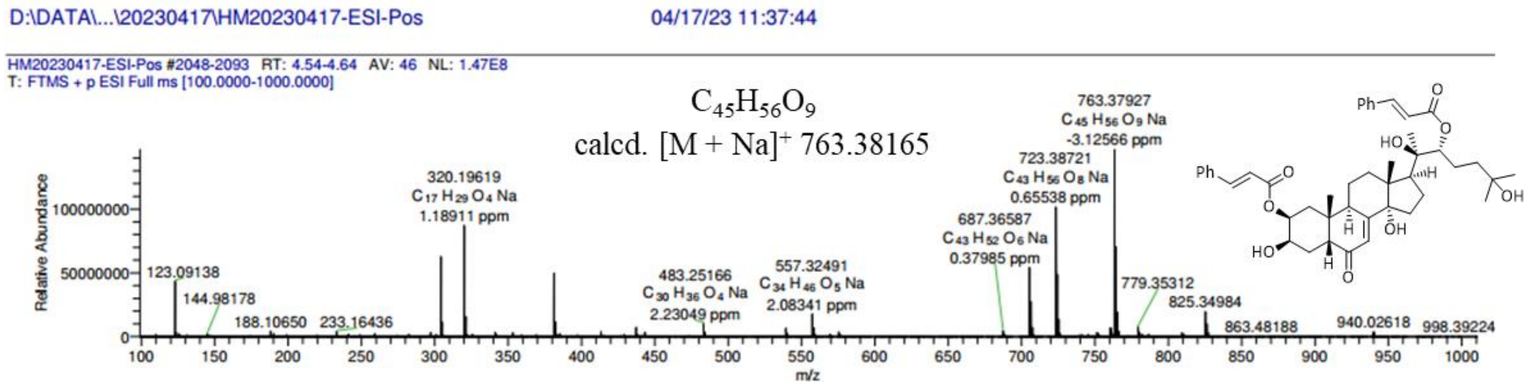

Figure S20. HR-MS spectrum of compound **40**

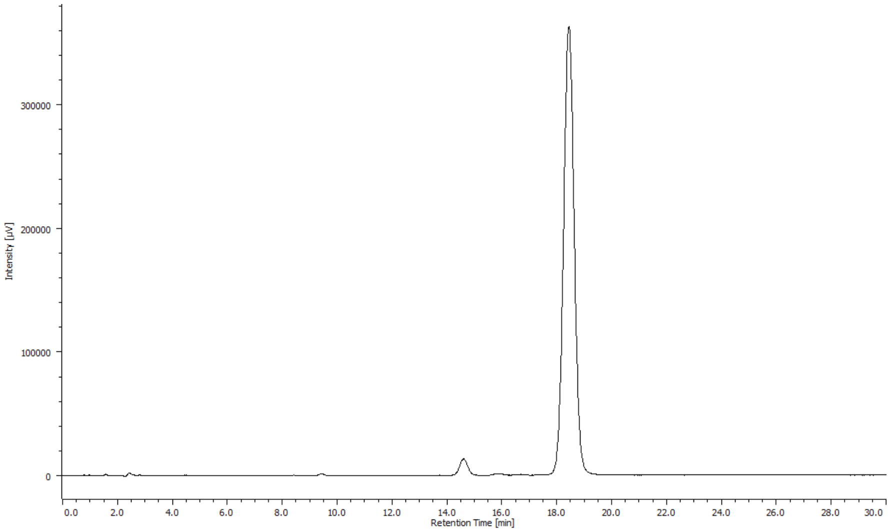

Figure S21. HPLC chromatogram of Compound **40** at its UV absorbance maximum(λ=276.0 nm). Purity 96.5 %. Column: Kinetex®, 5 µm, Biphenyl 100 Å, 250 × 4.6mm, (Phenomenex Inc.); Elution: H_2O:CH_3CN (A:B) 52 % B

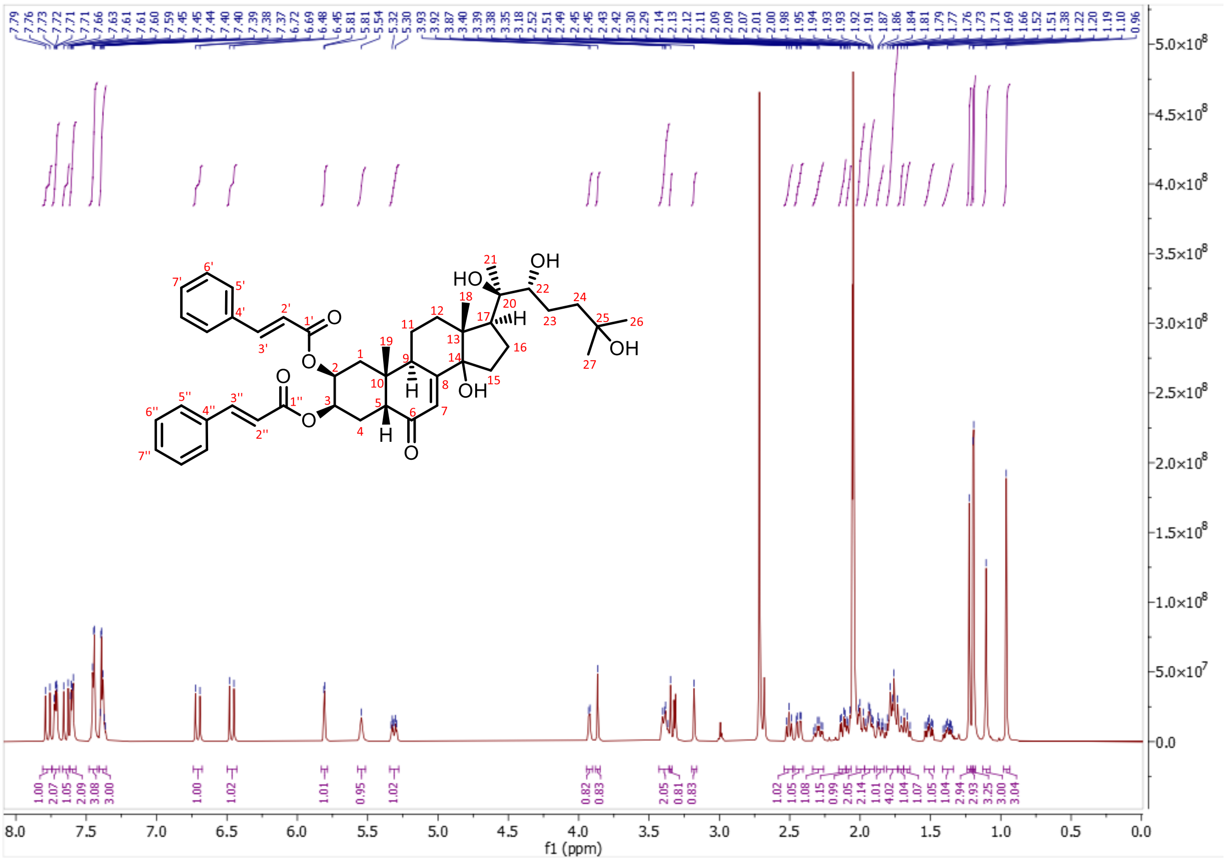

Figure S22. ^{1}H NMR (500 MHz, acetone-d_6) spectrum of compound **41** 20-hydroxyecdysone 2,3-dicinnamate

^{1}H NMR (500 MHz, acetone-d_6) δ 7.77 (d, J = 16.0 Hz, 1H, H-3′), 7.74 – 7.69 (m, 2H, H-5′), 7.64 (d, J = 16.0 Hz, 1H, H-3″), 7.62 – 7.57 (m, 2H, H-5″), 7.48 – 7.42 (m, 3H, H-6′ and H-7′), 7.41 – 7.36 (m, 3H, H-6″ and H-7″), 6.71 (d, J = 16.0 Hz, 1H, H-2′), 6.47 (d, J = 16.0 Hz, 1H, H-2″), 5.81 (d, J = 2.3 Hz, 1H, H-7), 5.54 (br s, 1H, H-3), 5.31 (dt, J = 12.3, 4.0 Hz, 1H, H-2), 3.92 (br d, J = 4.4 Hz, 1H, 22-OH), 3.87 (s, 1H, 14-OH), 3.43 – 3.36 (m, 2H, H-9 and H-22), 3.35 (s, 1H, 25-OH), 3.18 (s, 1H, 20-OH), 2.51 (dd, J = 9.2, 8.9 Hz, 1H, H-17), 2.44 (dd, J = 13.3, 4.1 Hz, 1H, H-5), 2.30 (td, J = 13.1, 4.9 Hz, 1H, H-12), 2.12 (dd, J = 13.3, 4.3 Hz, 1H, H-1), 2.10 – 2.07 (m, 1H, H-16), 2.02 – 1.97 (m, 2H, H-4 and H-15), 1.97 – 1.90 (m, 2H, H-11 and H-12), 1.86 (dt, J = 14.6, 3.6 Hz, 1H, H-4), 1.81 – 1.73 (m, 4H, H-1, H-11, H-16 and H-24), 1.71 (m, 1H, H-23), 1.69 – 1.64 (m, 1H, H-15), 1.54 – 1.47 (m, 1H, H-24), 1.41 – 1.34 (m, 1H, H-23), 1.22 (s, 3H, H-21), 1.20 (s, 3H, H-27), 1.19 (s, 3H, H-26), 1.10 (s, 3H, H-19), 0.96 (s, 3H, H-18).

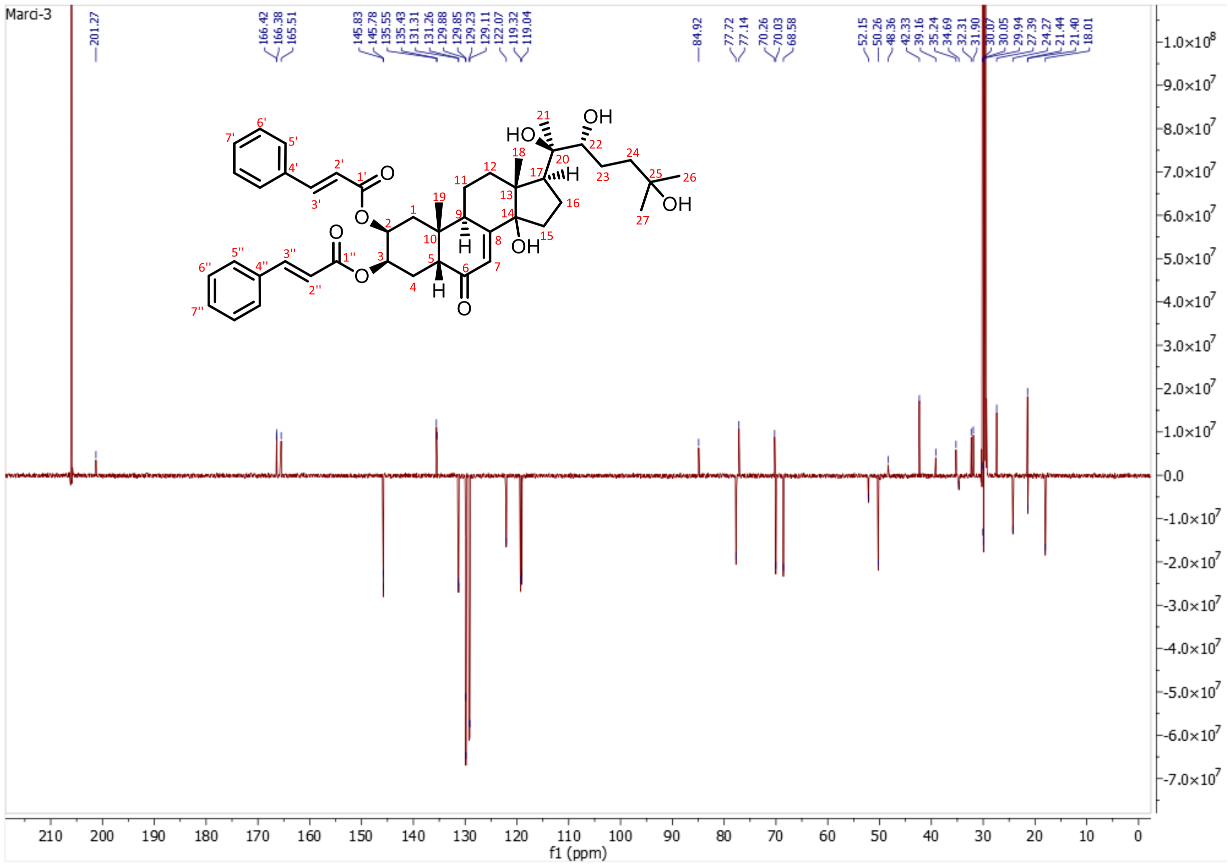

Figure S23. JMOD NMR (126 MHz, acetone-*d$_6$*) spectrum of compound **41** 20-hydroxyecdysone 2,3-dicinnamate

^{13}C NMR (126 MHz, acetone-*d$_6$*) δ 201.3 (C-6), 166.4 (C-1′), 166.4 (C-1″), 165.5 (C-8), 145.8 (C-3′), 145.8 (C-3″), 135.5 (C-4′), 135.4 (C-4″), 131.3 (C-7′), 131.3 (C-7″), 129.9 (C-6″), 129.9 (C-6′), 129.2 (C-5′), 129.1 (C-5″), 122.1 (C-7), 119.3 (C-2′), 119.0 (C-2″), 84.9 (C-14), 77.7 (C-22), 77.1 (C-20), 70.3 (C-25), 70.0 (C-2), 68.6 (C-3), 52.1 (C-5), 50.3 (C-17), 48.4 (C-13), 42.3 (C-24), 39.2 (C-10), 35.2 (C-1), 34.7 (C-9), 32.3 (C-12), 31.9 (C-15), 30.1 (C-4), 30.1 (C-27), 29.9 (C-26), 27.4 (C-23), 24.3 (C-19), 21.44 (C-11 and C-16), 21.40 (C-21), 18.0 (C-18).

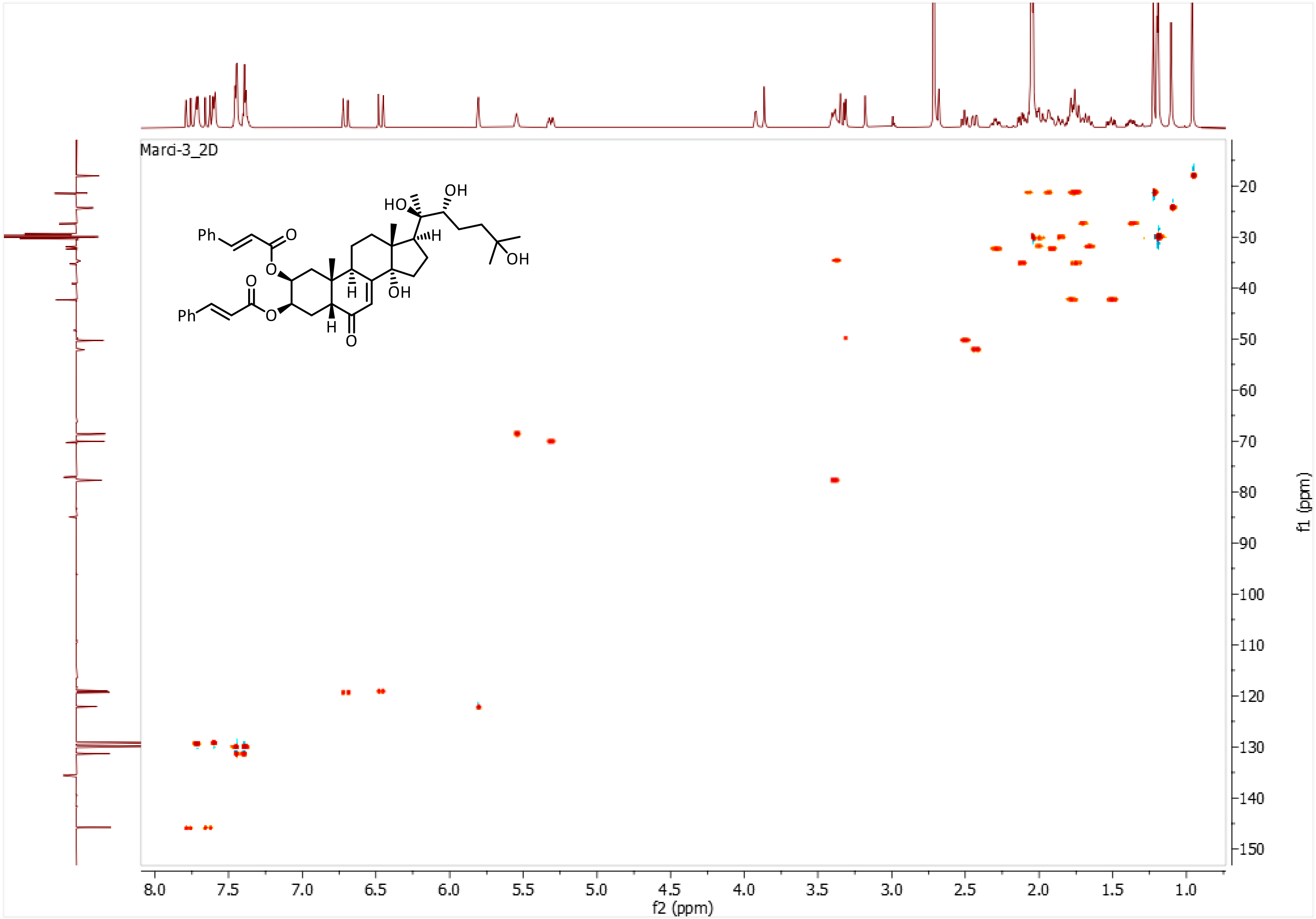

Figure S24. HSQC NMR (acetone-d_6) spectrum of compound **41**
20-hydroxyecdysone 2,3-dicinnamate

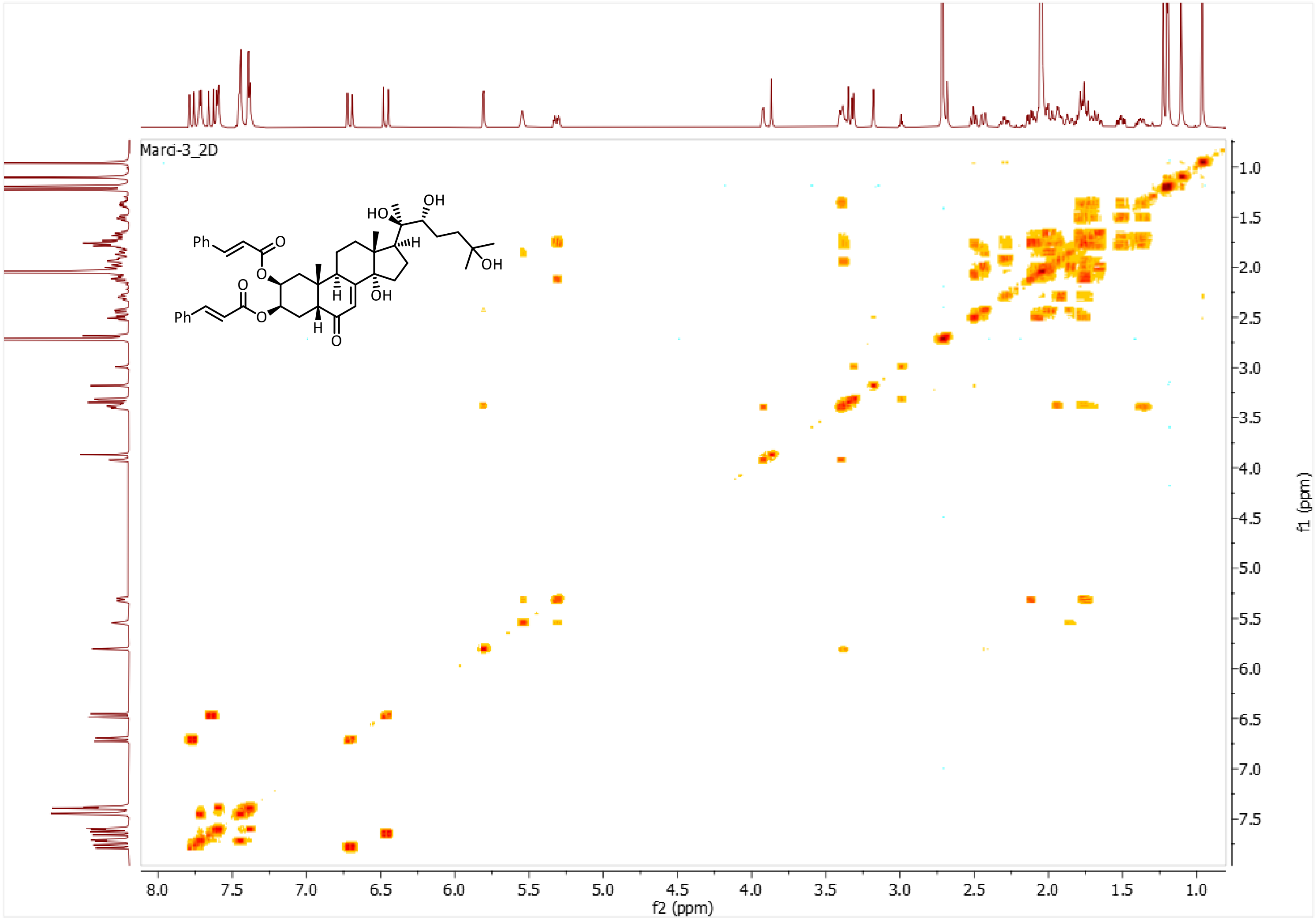

Figure S25. ^{1}H-^{1}H COSY NMR (acetone-d_6) spectrum of compound **41**
20-hydroxyecdysone 2,3-dicinnamate

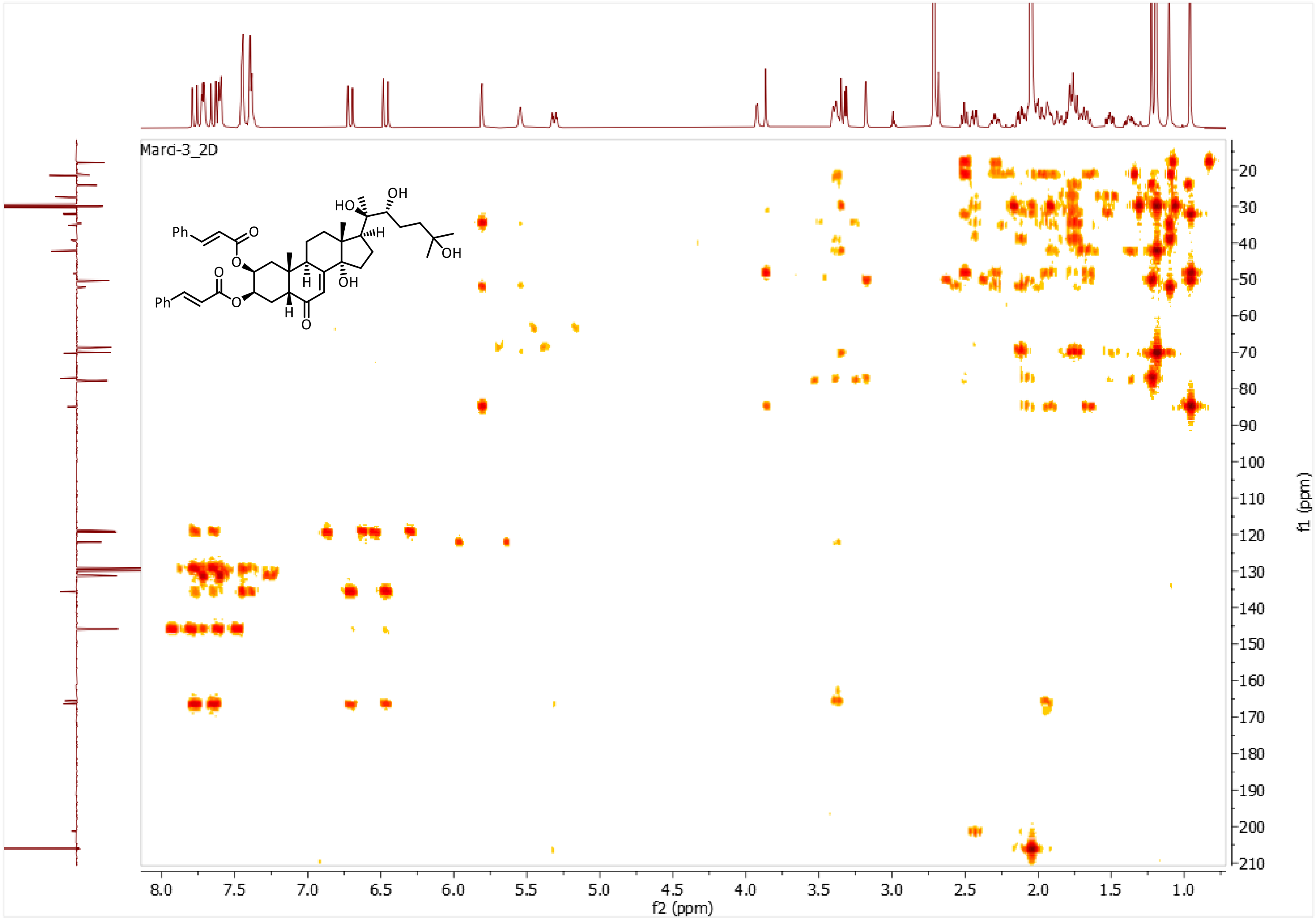

Figure S26. HMBC NMR (acetone-d_6) spectrum of compound **41**
20-hydroxyecdysone 2,3-dicinnamate

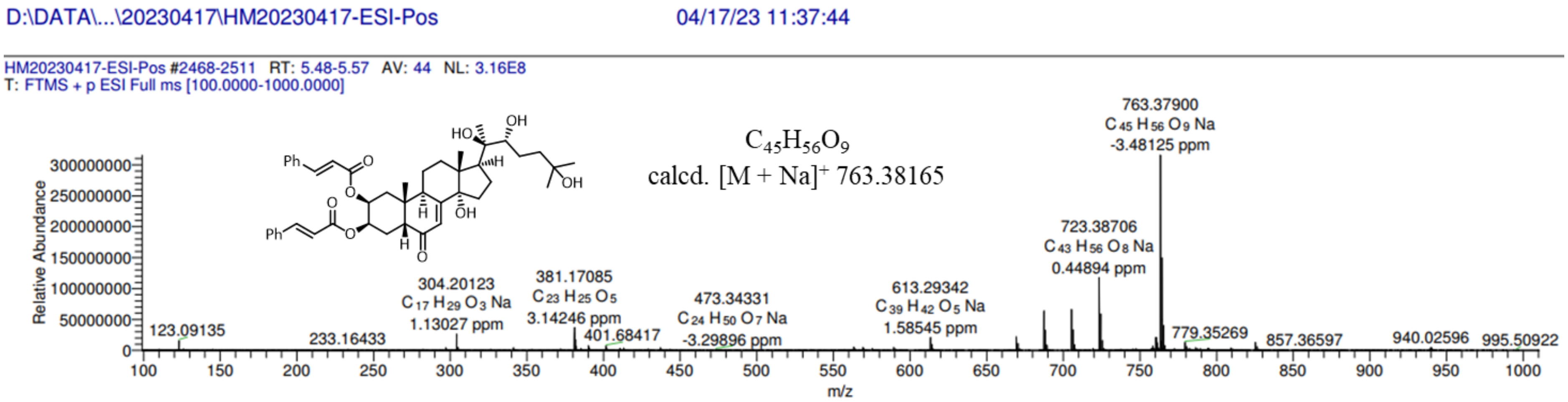

Figure S27. HR-MS spectrum of compound **41**

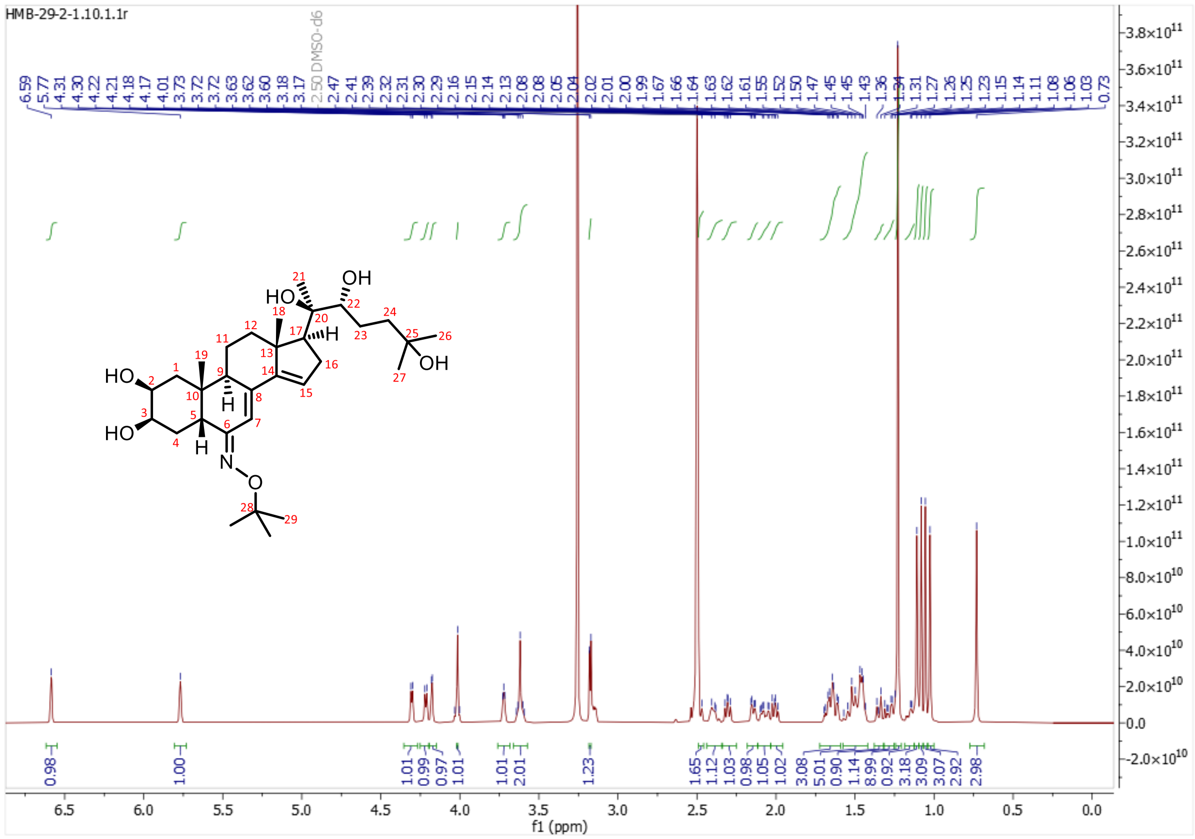

Figure S28. ^{1}H NMR (500 MHz, DMSO-d_6) of compound **42**
Stachysterone B 6-*O*-*tert*-butyl oxime ether (*E* isomer)

^{1}H NMR (500 MHz, DMSO-d_6) δ 6.59 (s, 1H, H-7), 5.77 (s, 1H, H-15), 4.30 (d, J = 5.2 Hz, 1H, 22-OH), 4.22 (d, J = 6.1 Hz, 1H, 2-OH), 4.19 – 4.15 (m, 1H, 3-OH), 4.01 (s, 1H, 25-OH), 3.76 – 3.68 (m, 1H, H-3), 3.66 – 3.57 (m, 2H, H-2 and 20-OH), 3.18 (d, J = 5.2 Hz, 1H, H-22), 2.47 (s, 1H, H-16), 2.44 – 2.34 (m, 1H, H-9), 2.34 – 2.25 (m, 1H, H-5), 2.18 – 2.12 (m, 1H, H-12), 2.12 – 2.03 (m, 1H, H-16), 2.03 – 1.96 (m, 1H, H-17), 1.72 – 1.59 (m, 3H, H-1, H-11 and H-24), 1.58 – 1.42 (m, 5H, H-4, H-11, H-12, H-23), 1.38 – 1.32 (m, 1H, H-1), 1.32 – 1.25 (m, 1H, H-24), 1.23 (s, 9H, H-29), 1.18 – 1.13 (m, 1H, H-23), 1.11 (s, 3H, H-21), 1.08 (s, 3H, H-26), 1.06 (s, 3H, H-27), 1.03 (s, 3H, H-18), 0.73 (s, 3H, H-19).

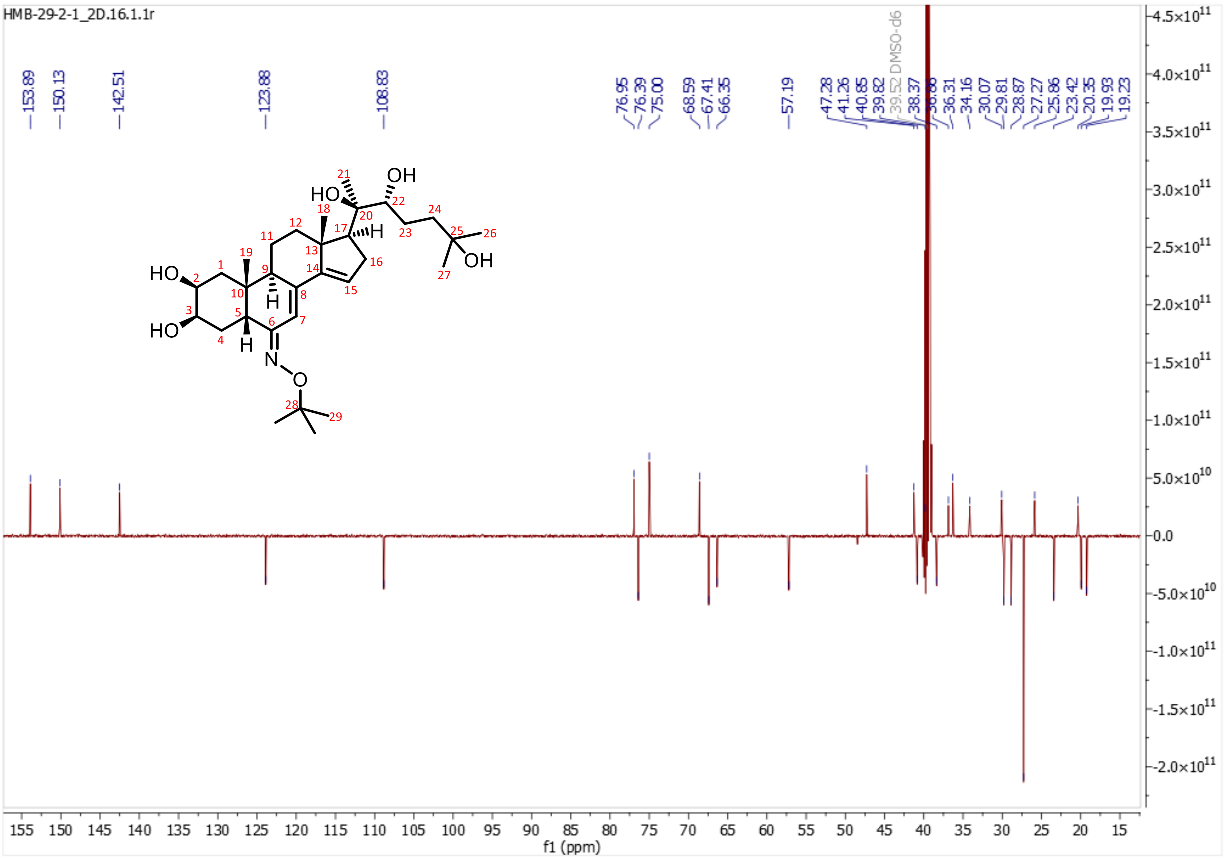

Figure S29. JMOD NMR (126 MHz, DMSO-d_6) of compound **42**
Stachysterone B 6-*O*-*tert*-butyl oxime ether (*E* isomer)

^{13}C NMR (126 MHz, DMSO-d6) δ 153.9 (C-6), 150.1 (C-14), 142.5 (C-8), 123.9 (C-15), 108.8 (C-7), 76.9 (C-28), 76.4 (C-22), 75.0 (C-20), 68.6 (C-25), 67.4 (C-3), 66.4 (C-2), 57.2 (C-17), 47.3 (C-13), 41.3 (C-24), 40.8 (C-5), 39.8 (C-12), 38.4 (C-9), 36.9 (C-1), 36.3 (C-10), 34.2 (C-4), 30.1 (C-16), 29.8 (C-26), 28.9 (C-27), 27.3 (C-29), 25.9 (C-23), 23.4 (C-19), 20.3 (C-11), 19.9 (C-21), 19.2 (C-18).

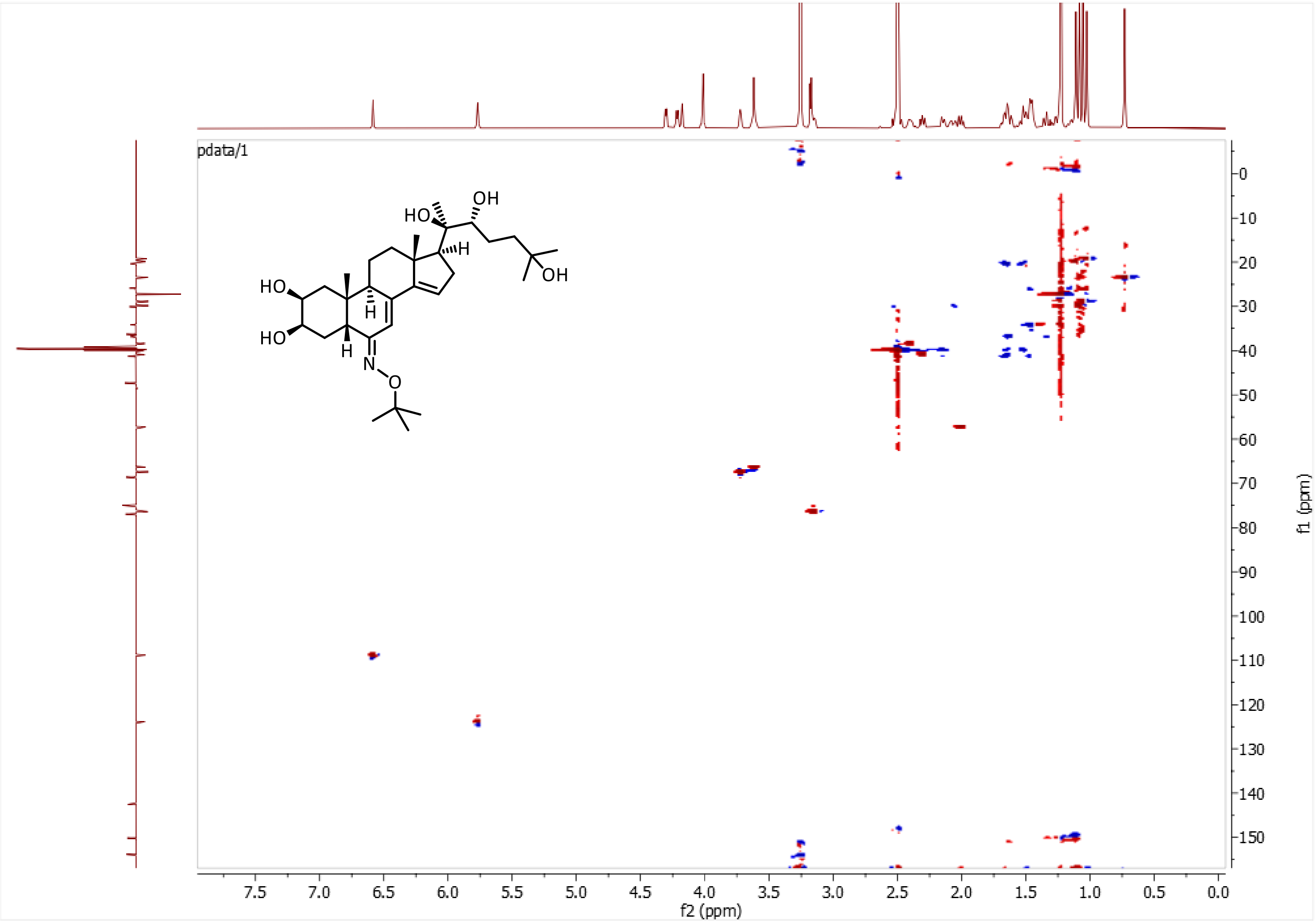

Figure S30. HSQC NMR (DMSO-d_6) spectrum of compound **42**
Stachysterone B 6-*O*-*tert*-butyl oxime ether (*E* isomer)

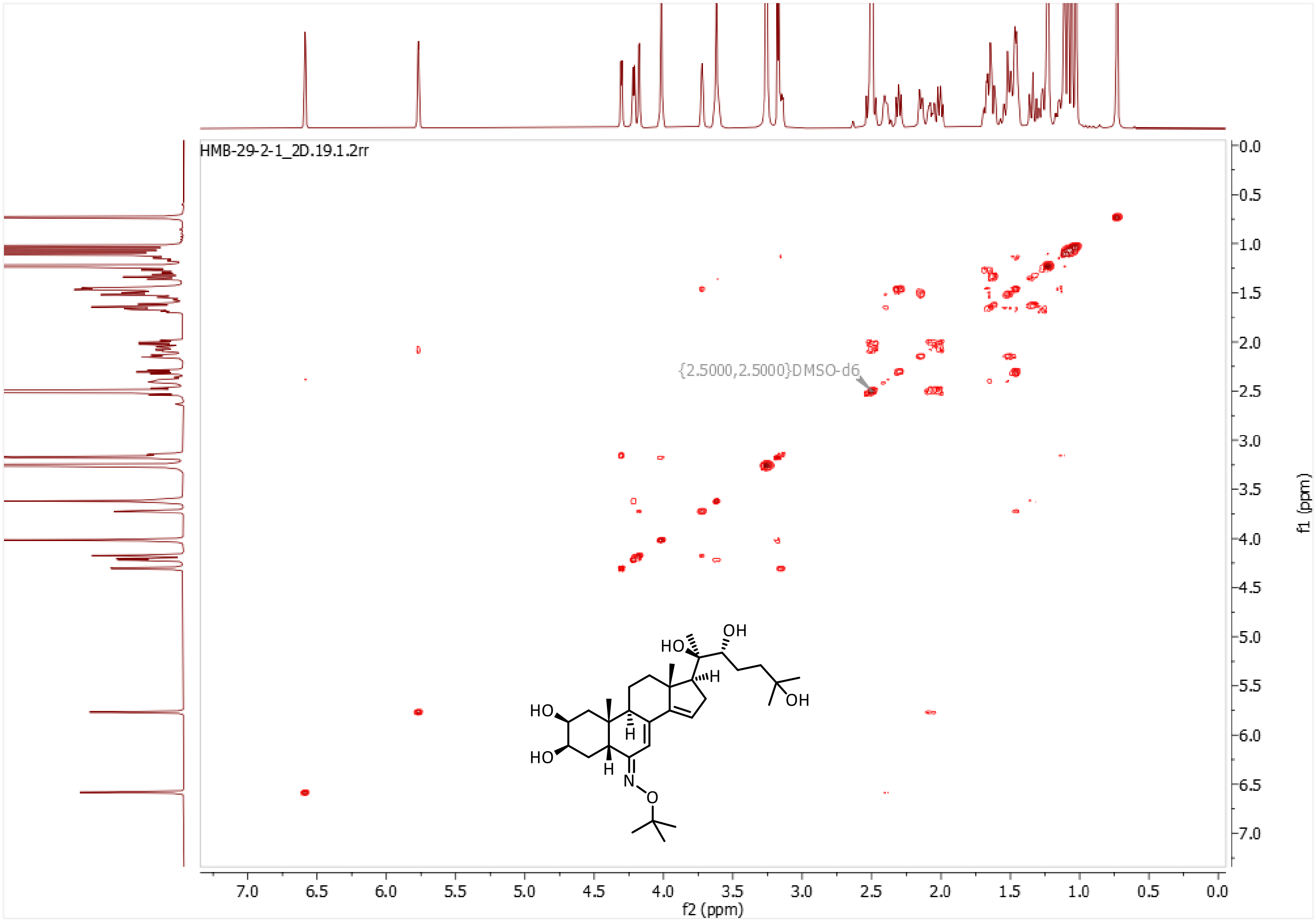

Figure S31. ^{1}H-^{1}H COSY NMR (DMSO-d_6) spectrum of compound **42**
Stachysterone B 6-*O*-*tert*-butyl oxime ether (*E* isomer)

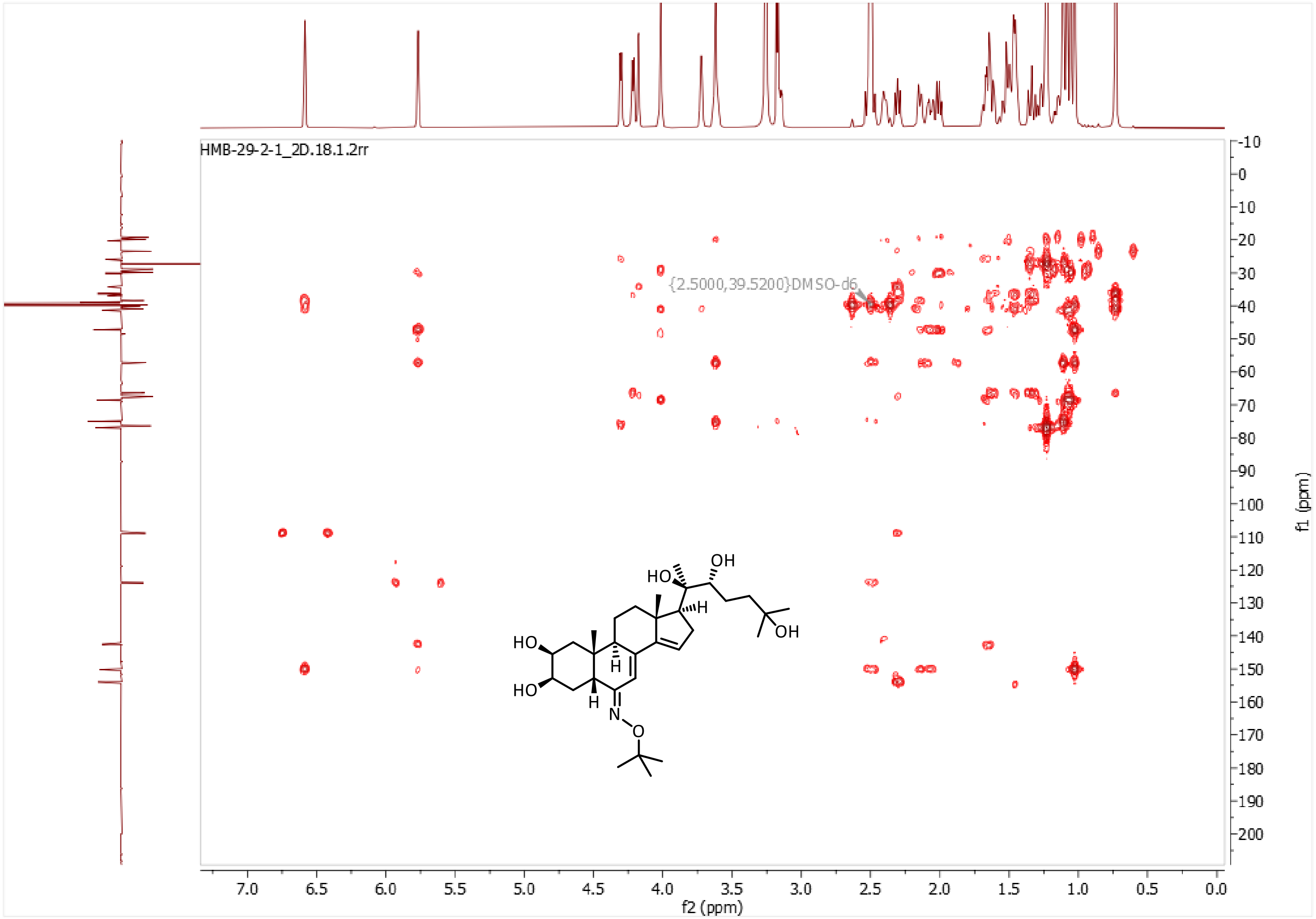

Figure S32. HMBC NMR (DMSO-d_6) spectrum of compound **42**
Stachysterone B-6-*O*-*tert*-butyl oxime ether (*E* isomer)

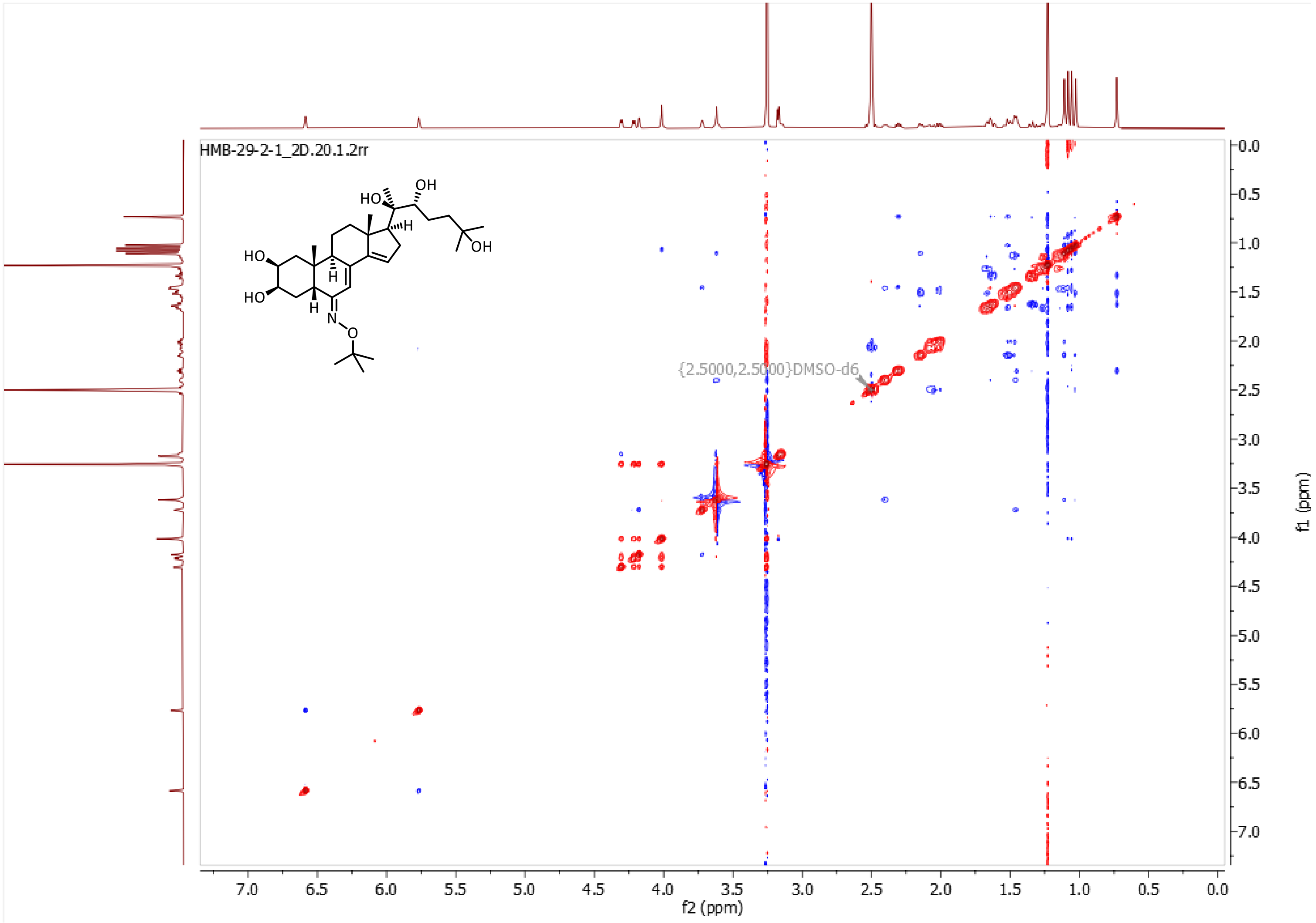

Figure S33. ROESY (DMSO-d_6) NMR spectrum of compound **42**
Stachysterone B 6-*O*-*tert*-butyl oxime ether (*E* isomer)

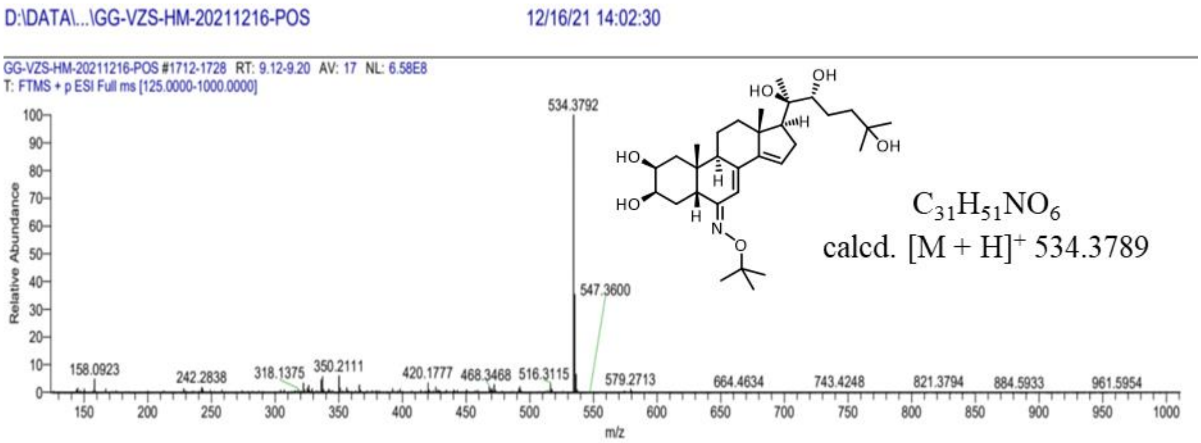

Figure S34. HR-MS spectrum of compound **42**

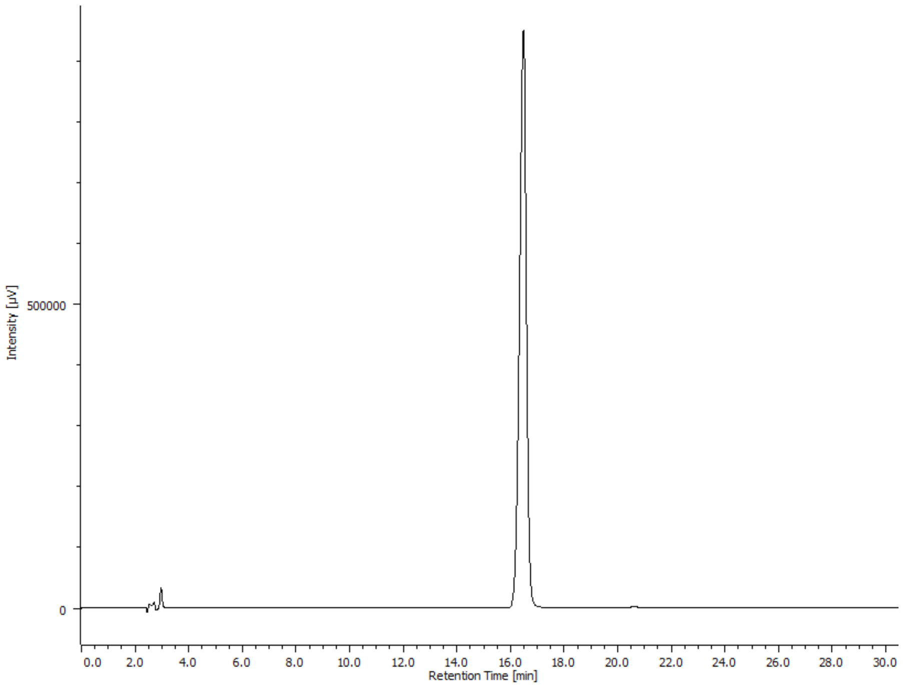

Figure S35. HPLC chromatogram of compound **42** at its UV absorbance maximum (λ=294.5 nm). Purity 98.6 %.
Column: Kinetex®, 5µm, XB-C18, 100 Å, 250 × 4.6 mm (Phenomenex Inc.); Elution: H_2O:CH_3CN (A:B) 35 % B.

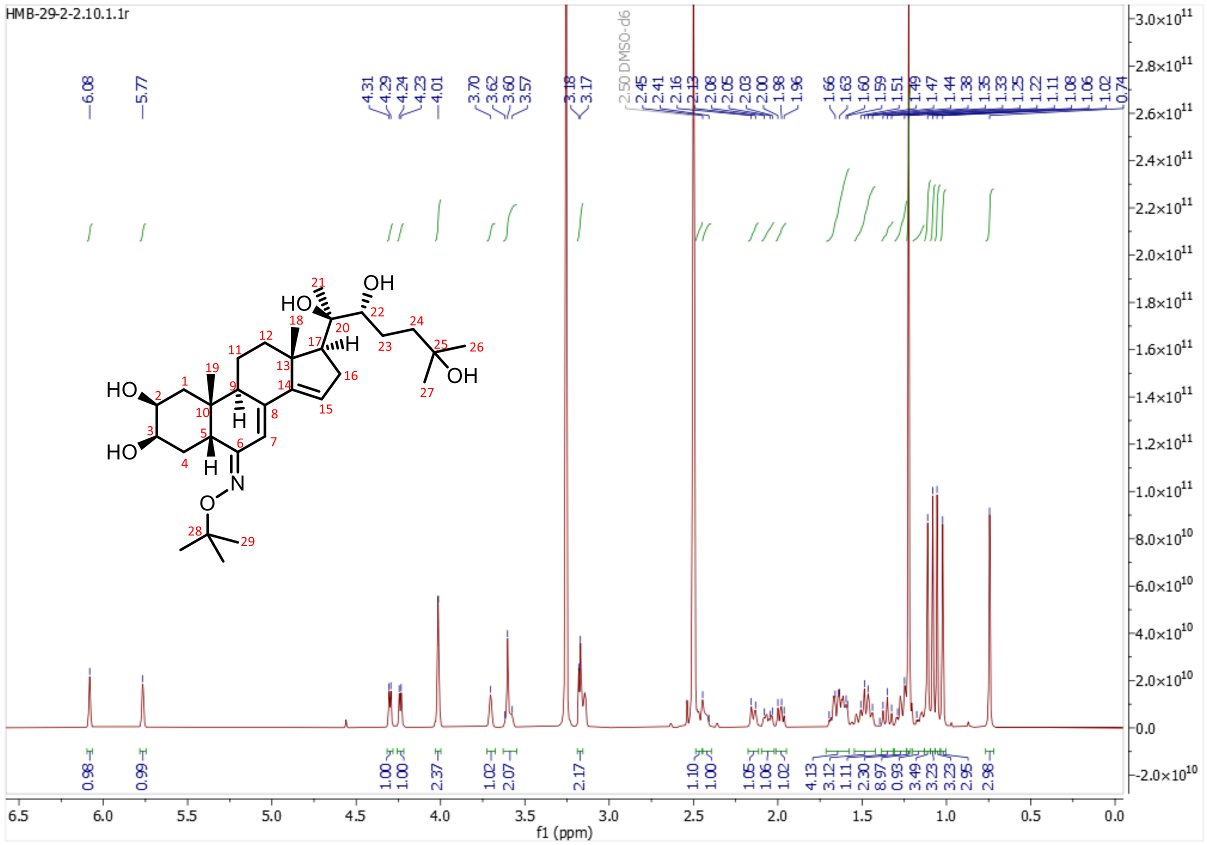

Figure S36. ^{1}H NMR (500 MHz, DMSO-d_6) spectrum of compound **43**
Stachysterone B 6-*O*-*tert*-butyl oxime ether (*Z* isomer)

^{1}H NMR (500 MHz, DMSO-d_6) δ 6.08 (s, 1H, H-7), 5.77 (s, 1H, H-15), 4.30 (d, J = 5.2 Hz, 1H, 22-OH), 4.24 (d, J = 5.8 Hz, 1H, 2-OH), 4.05 – 3.98 (m, 2H, 3-OH and 25-OH), 3.70 (s, 1H, H-3), 3.63 – 3.55 (m, 2H, H-2 and 20-OH), 3.19 – 3.16 (m, 2H, H-5 and H-22), 2.49 – 2.39 (m, 2H, H-9 and H-16), 2.18 – 2.11 (m, 1H, H-12), 2.10 – 2.02 (m, 1H, H-16), 2.01 – 1.95 (m, 1H, H-17), 1.71 – 1.58 (m, 4H, H-1, H-4, H-11 and H-24), 1.55 – 1.42 (m, 3H, H-11, H-12 and H-23), 1.35 (t, J = 12.7 Hz, 1H, H-1), 1.31 – 1.24 (m, 2H, H-4 and H-24), 1.22 (s, 9H, H-29), 1.20 – 1.13 (m, 1H, H-23), 1.11 (s, 3H, H-21), 1.08 (s, 3H, H-26), 1.06 (s, 3H, H-27), 1.02 (s, 3H, H-18), 0.74 (s, 3H, H-19).

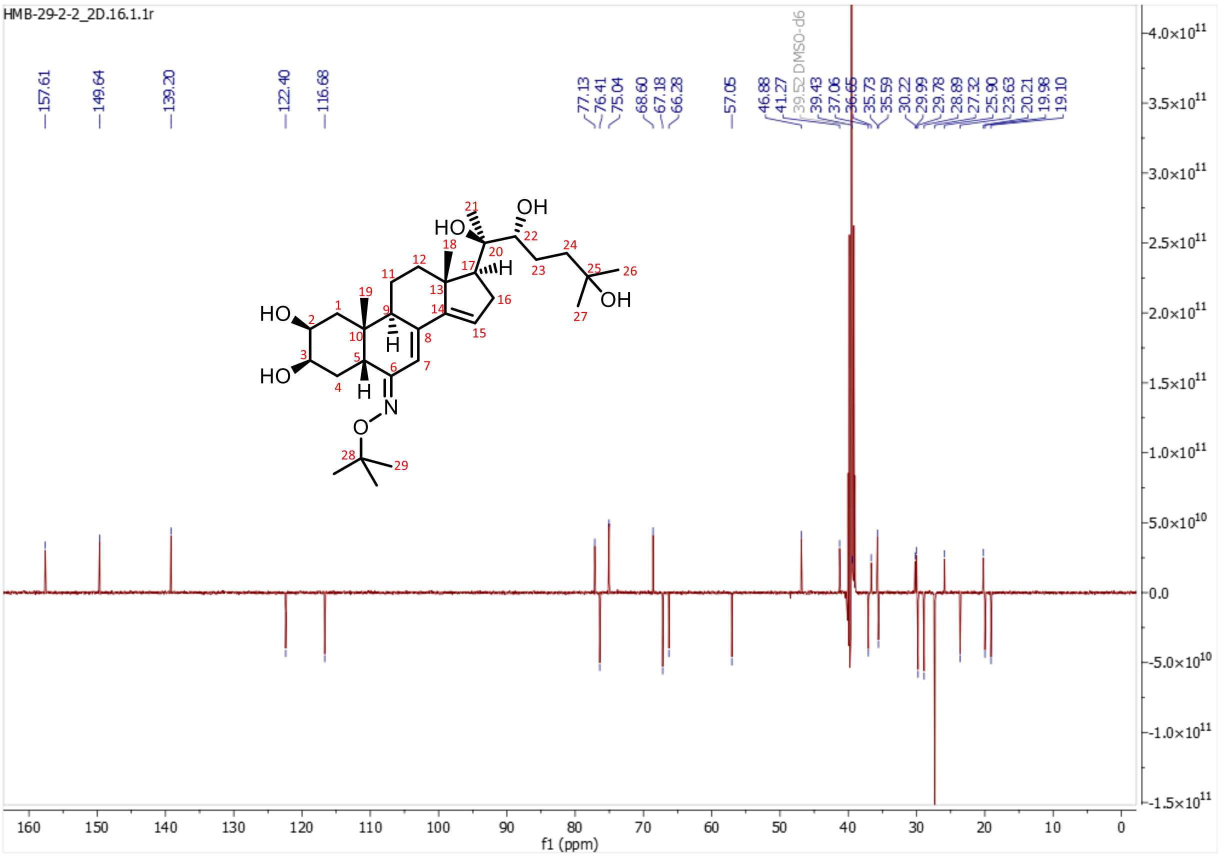

Figure S37. JMOD NMR (126 MHz, DMSO-d_6) spectrum of compound **43**
Stachysterone B 6-*O*-*tert*-butyl oxime ether (*Z* isomer)

^{13}C NMR (126 MHz, DMSO-d_6) δ 157.6 (C-6), 149.6 (C-14), 139.2 (C-8), 122.4 (C-15), 116.7 (C-7), 77.1 (C-28), 76.4 (C-22), 75.0 (C-20), 68.6 (C-25), 67.2 (C-3), 66.3 (C-2), 57.1 (C-17), 46.9 (C-13), 41.3 (C-24), 39.4 (C-12), 37.1 (C-9), 36.6 (C-1), 35.7 (C-10), 35.6 (C-5), 30.2 (C-4), 30.0 (C-16), 29.8 (C-26), 28.9 (C-27), 27.3 (C-29), 25.9 (C-23), 23.6 (C-19), 20.2 (C-11), 20.0 (C-21), 19.1 (C-18).

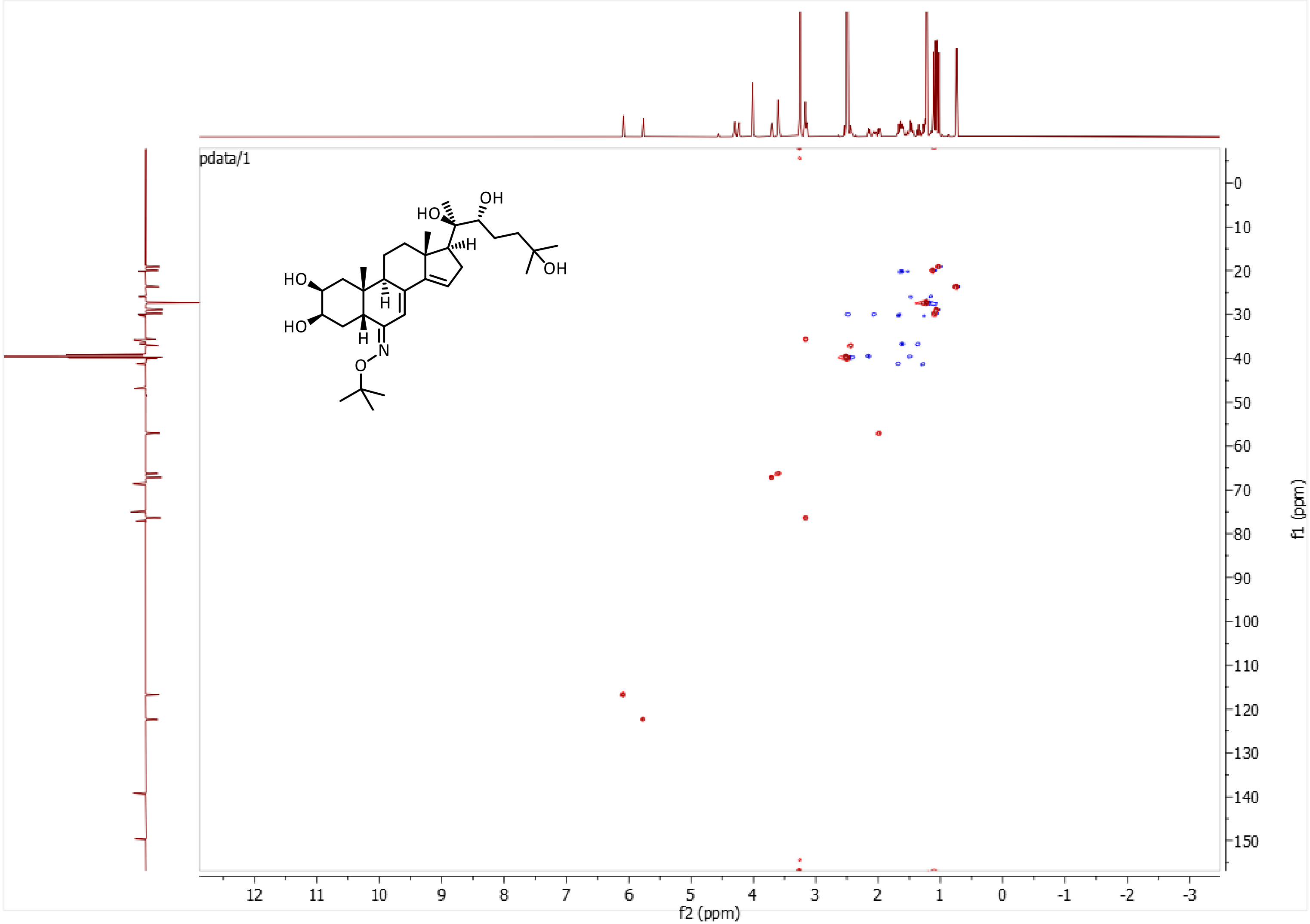

Figure S38. HSQC NMR (DMSO-d_6) spectrum of compound **43**
Stachysterone B 6-*O*-*tert*-butyl oxime ether (*Z* isomer)

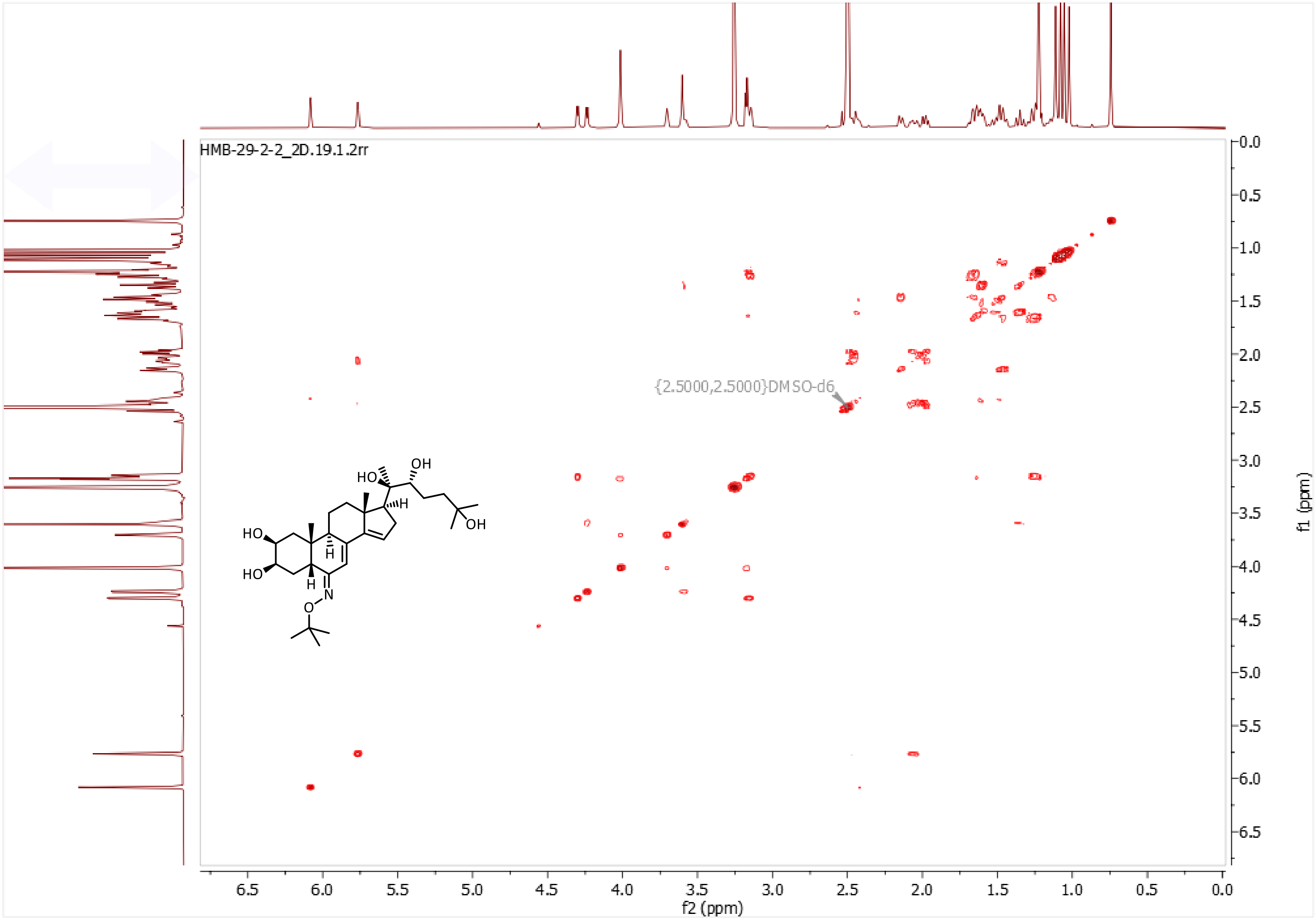

Figure S39. ^{1}H-^{1}H COSY NMR (DMSO-d_6) spectrum of compound **43**
Stachysterone B 6-*O*-*tert*-butyl oxime ether (*Z* isomer)

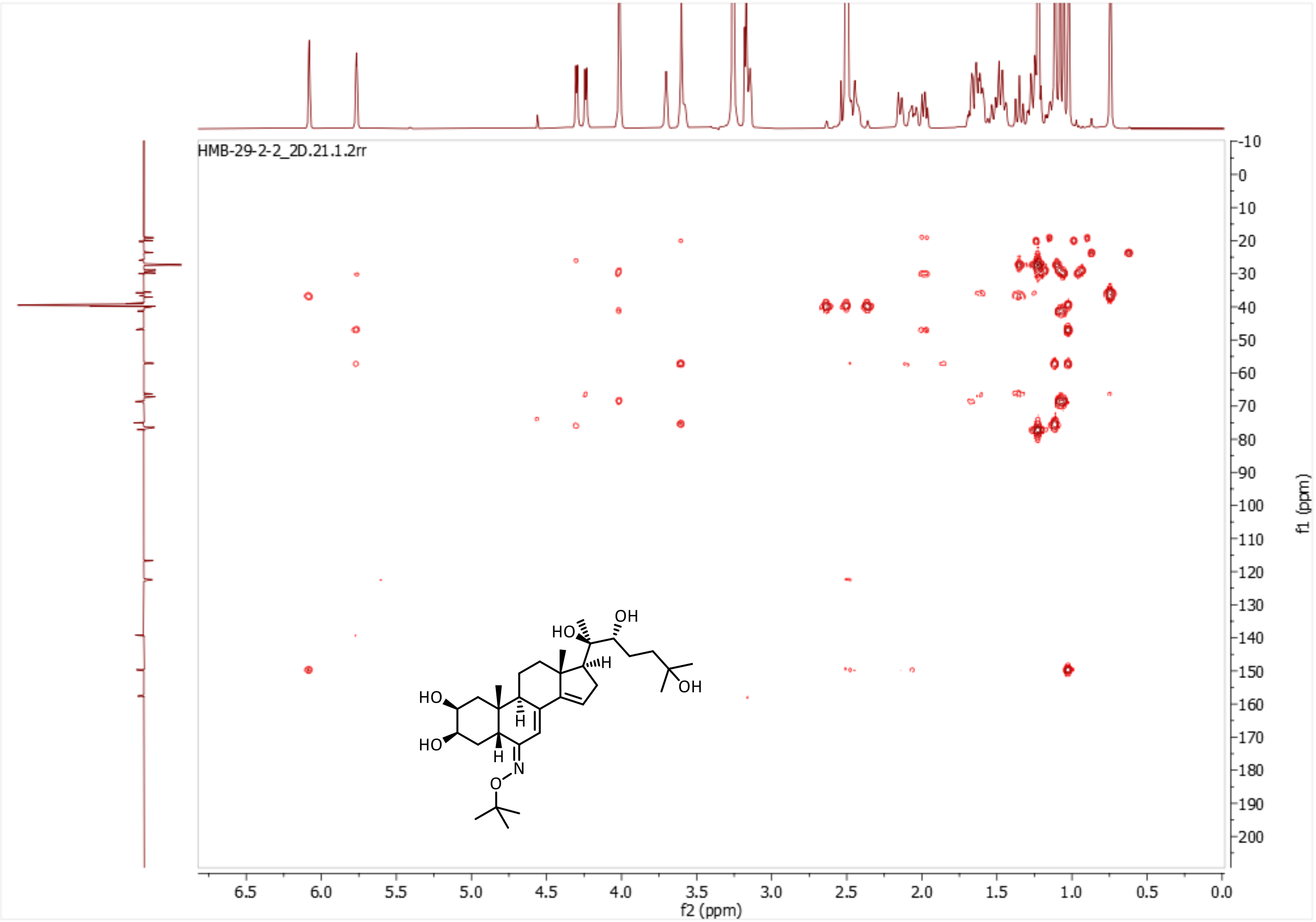

Figure S40. HMBC NMR (DMSO-d_6) spectrum of compound **43**
Stachysterone B 6-*O*-*tert*-butyl oxime ether (*Z* isomer)

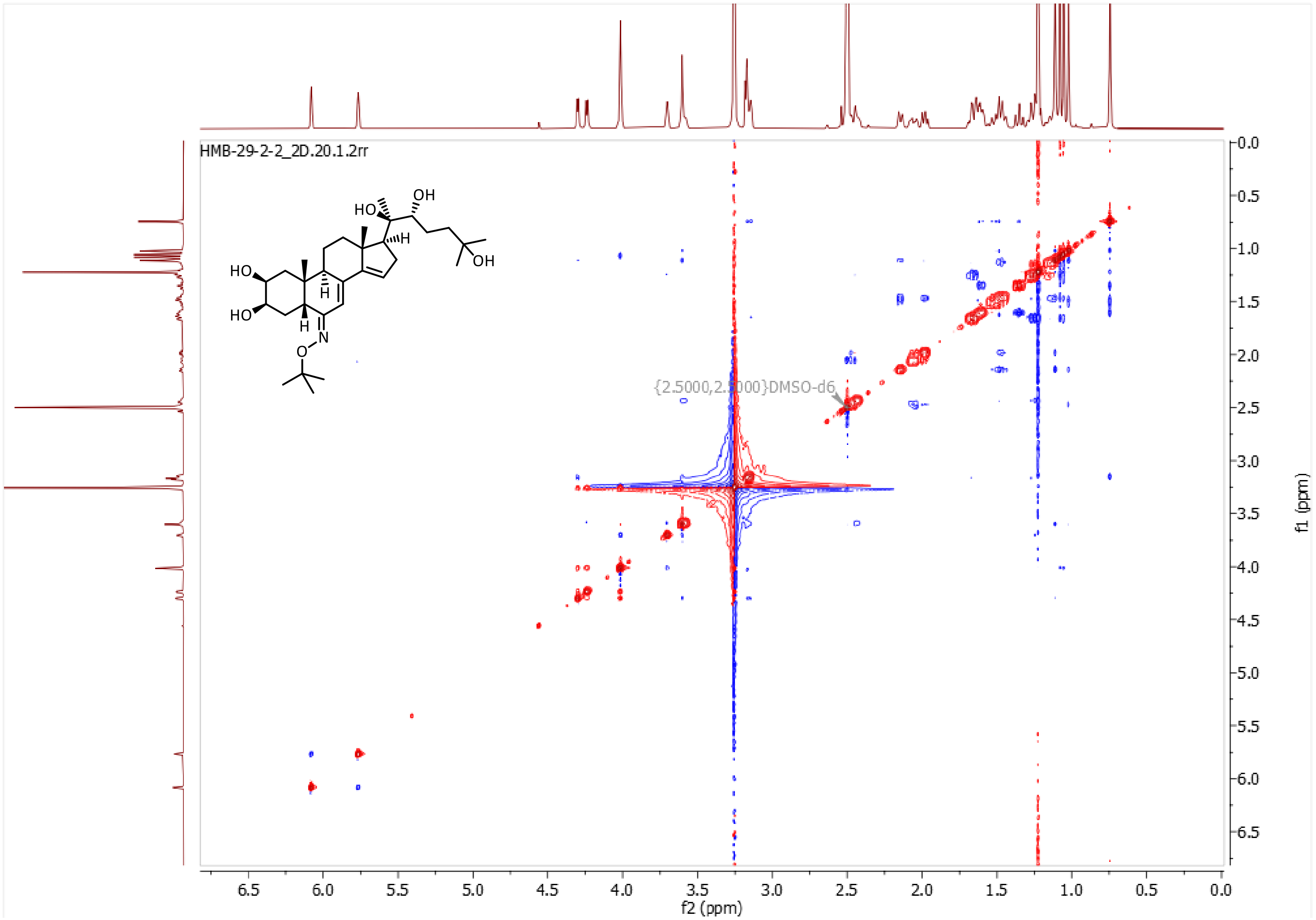

Figure S41. ROESY NMR (DMSO-d_6) spectrum of compound **43**
Stachysterone B-6-*O*-*tert*-butyl oxime ether (*Z* isomer)

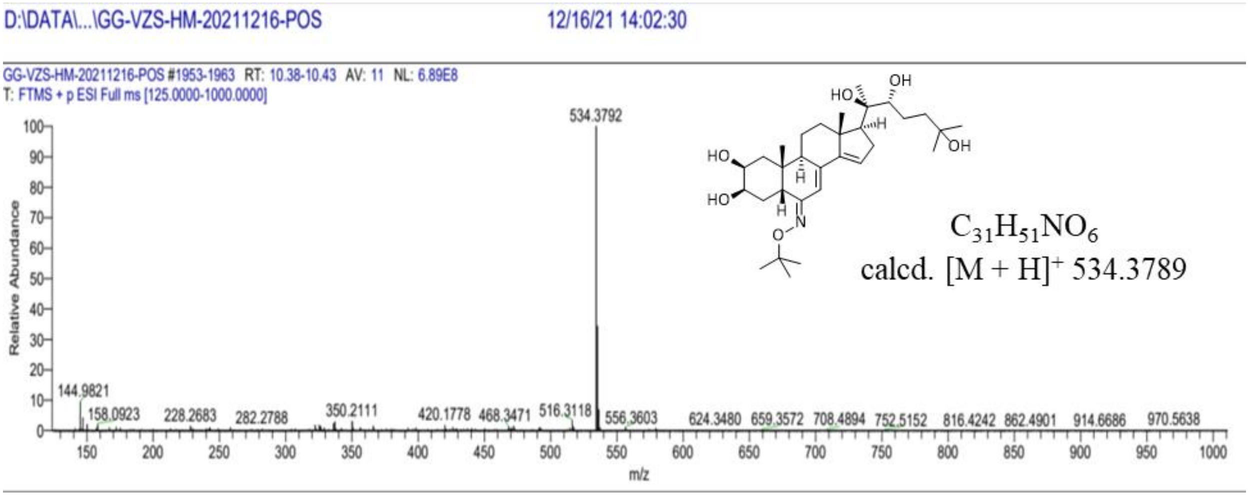

Figure S42. HR-MS spectrum of compound **43**

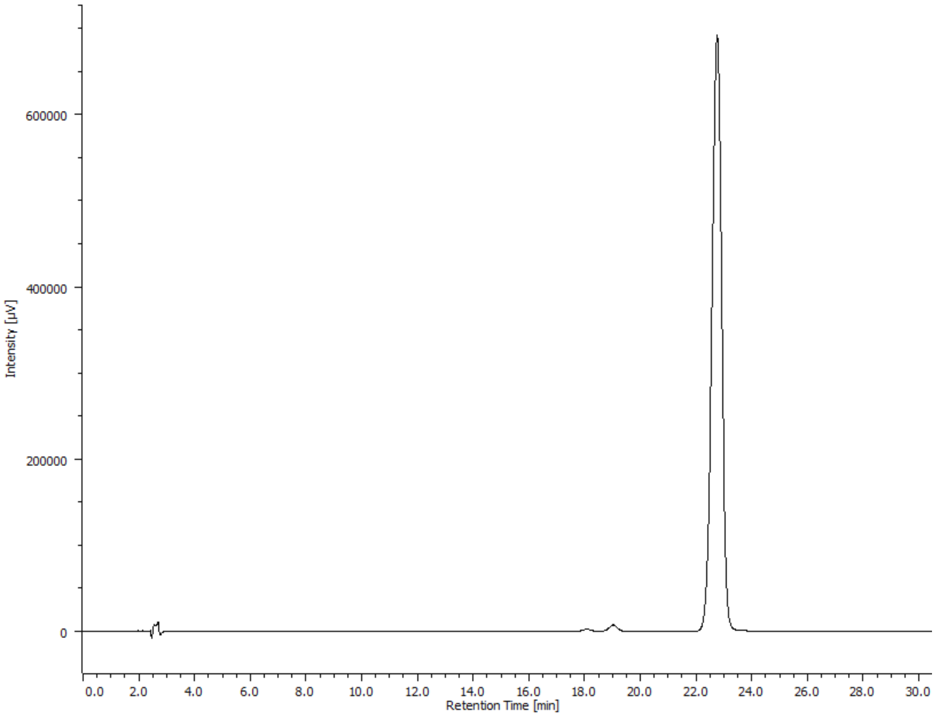

Figure S43. HPLC chromatogram of compound **43** at its UV absorbance maximum (λ=291.1 nm) Purity 98.4 % .
Column: Kinetex®, 5µm, XB-C18, 100 Å, 250 × 4.6 mm (Phenomenex Inc.); Elution: H_2O:CH_3CN (A:B) 35 % B.

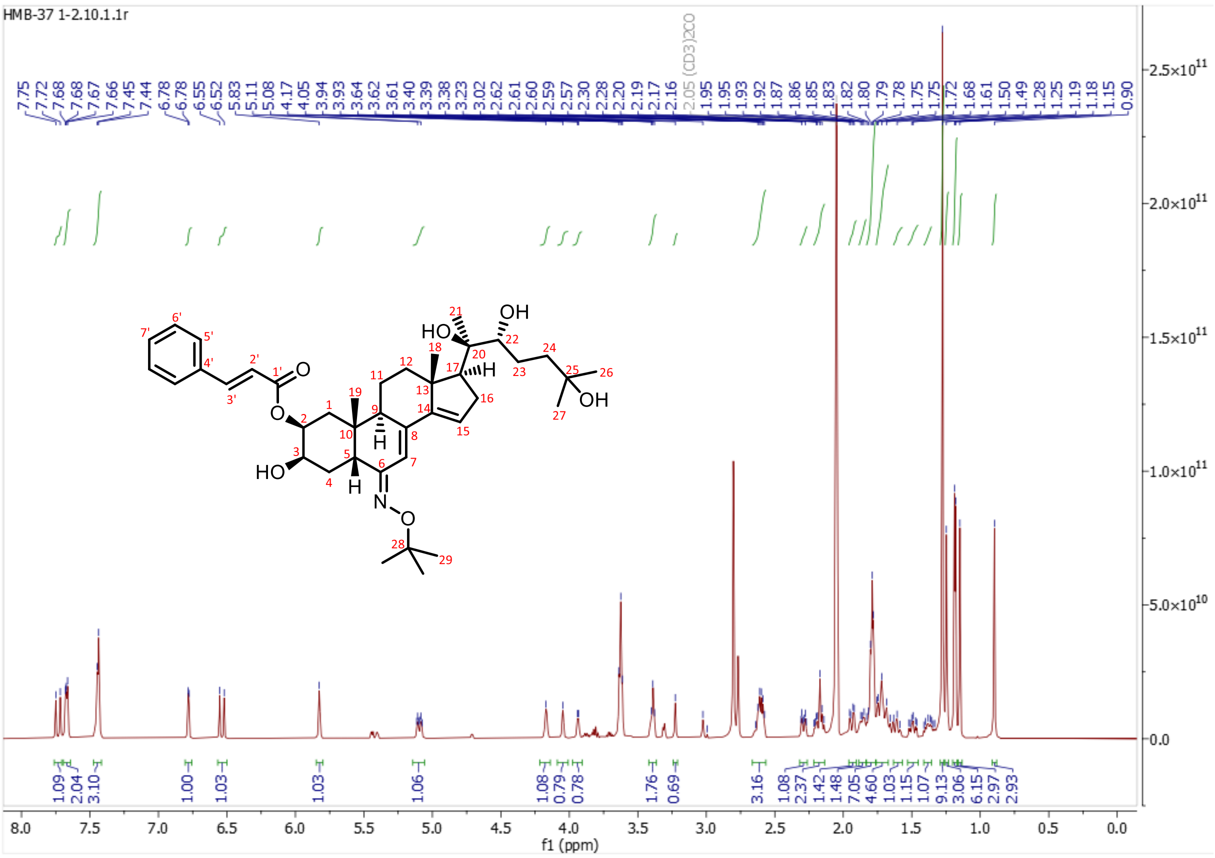

Figure S44. ^{1}H NMR (500 MHz, acetone-d_6) of compound **44**
Stachysterone B 6-*O*-*tert*-butyl oxime ether 2-cinnamate (*E* isomer). The NMR sample contained some tetrahydrofuran as well (overlapping at around 1.79 ppm): ^{1}H NMR δ 3.63 (t, J = 6.1 Hz), 1.80 – 1.77 (m).

^{1}H NMR (500 MHz, acetone-d_6) δ 7.73 (d, J = 16.1 Hz, 1H, H-3′), 7.67 (dd, J = 5.3, 2.7 Hz, 2H, H-5′), 7.44 (d, J = 5.0 Hz, 3H, H-6′ and H-7′), 6.78 (d, J = 3.1 Hz, 1H, H-7), 6.54 (d, J = 16.0 Hz, 1H, H-2′), 5.83 (s, 1H, H-15), 5.09 (dt, J = 12.2, 3.9 Hz, 1H, H-2), 4.17 (s, 1H, H-3), 4.05 (s, 1H, 3-OH), 3.94 (d, J = 4.9 Hz, 1H, 22-OH), 3.42 – 3.36 (m, 2H, H-22 and 25-OH), 3.23 (s, 1H, 20-OH), 2.61 (dq, J = 11.9, 6.6, 5.3 Hz, 3H, H-5, H-9 and H-16), 2.29 (dd, J = 12.4, 2.8 Hz, 1H, H-12), 2.23 – 2.12 (m, 2H, H-16 and H-17), 1.94 (dd, J = 12.7, 4.2 Hz, 1H, H-1), 1.88 – 1.83 (m, 1H, H-11), 1.83 – 1.76 (m, 2H, H-1 and H-24), 1.76 – 1.67 (m, 4H, H-4, H-11 and H-23), 1.65 – 1.57 (m, 1H, H-12), 1.54 – 1.44 (m, 1H, H-24), 1.43 – 1.33 (m, 1H, H-23), 1.28 (s, 9H, H-29), 1.25 (s, 3H, H-21), 1.19 (s, 3H, H-27), 1.18 (s, 3H, H-26), 1.15 (s, 3H, H-18), 0.90 (s, 3H, H-19).

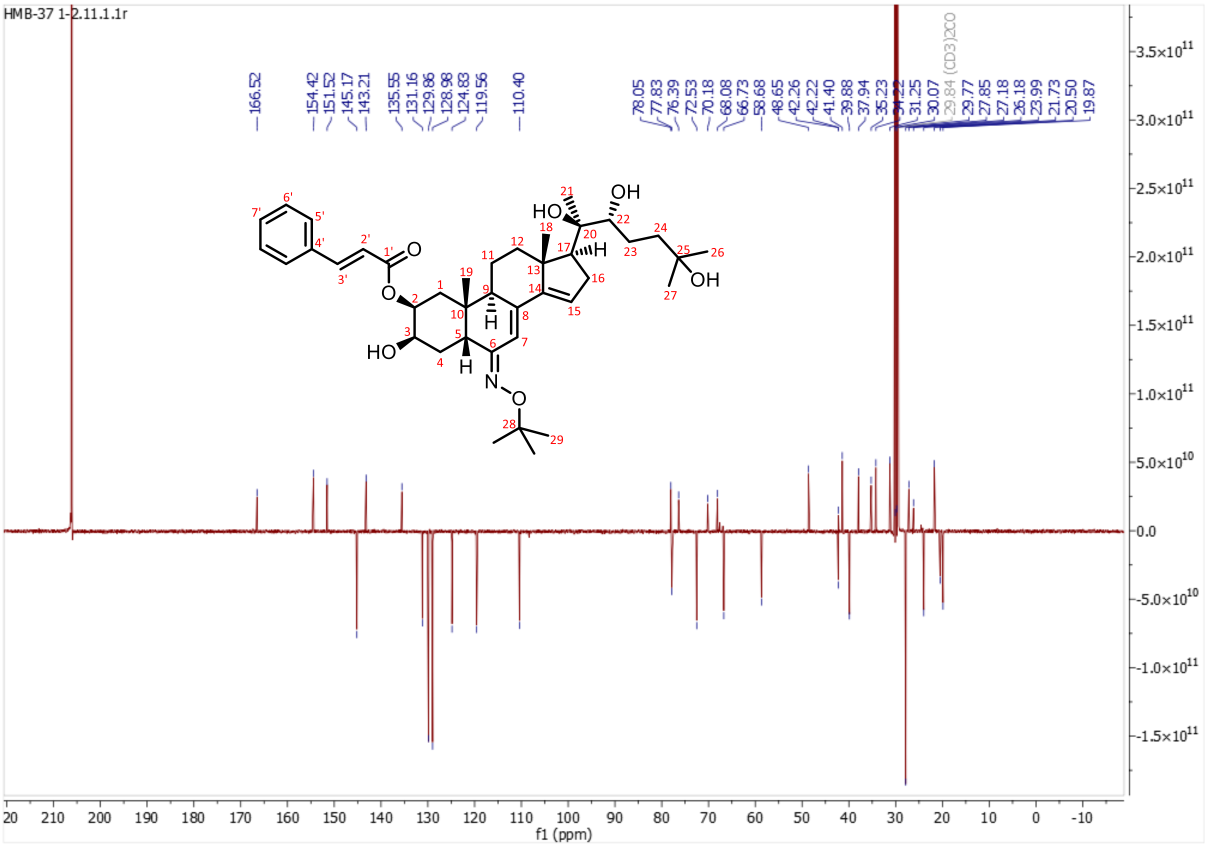

Figure S45. JMOD NMR (126 MHz, acetone-d_6) spectrum of compound **44** Stachysterone B 6-*O*-*tert*-butyl oxime ether 2-cinnamate (*E* isomer). The NMR sample contained some tetrahydrofuran as well: ^{13}C NMR δ 68.1, 26.

^{13}C NMR (126 MHz, acetone-d_6) δ 166.5 (C-1′), 154.4 (C-6), 151.5 (C-14), 145.2 (C-3′), 143.2 (C-8), 135.5 (C-4′), 131.2 (C-7′), 129.9 (C-6′), 129.0 (C-5′), 124.8 (C-15), 119.6 (C-2′), 110.4 (C-7), 78.0 (C-28), 77.8 (C-22), 76.4 (C-20), 72.5 (C-2), 70.2 (C-25), 66.7 (C-3)58.7 (C-17), 48.6 (C-13), 42.3 (C-5), 42.2 (C-24), 41.4 (C-12), 39.9 (C-9), 37.9 (C-10), 35.2 (C-4), 34.2 (C-1), 31.2 (C-16), 30.1 (C-27), 29.8 (C-26), 27.8 (C-29), 27.2 (C-23), 24.0 (C-19), 21.7 (C-11), 20.5 (C-21), 19.9 (C-18).

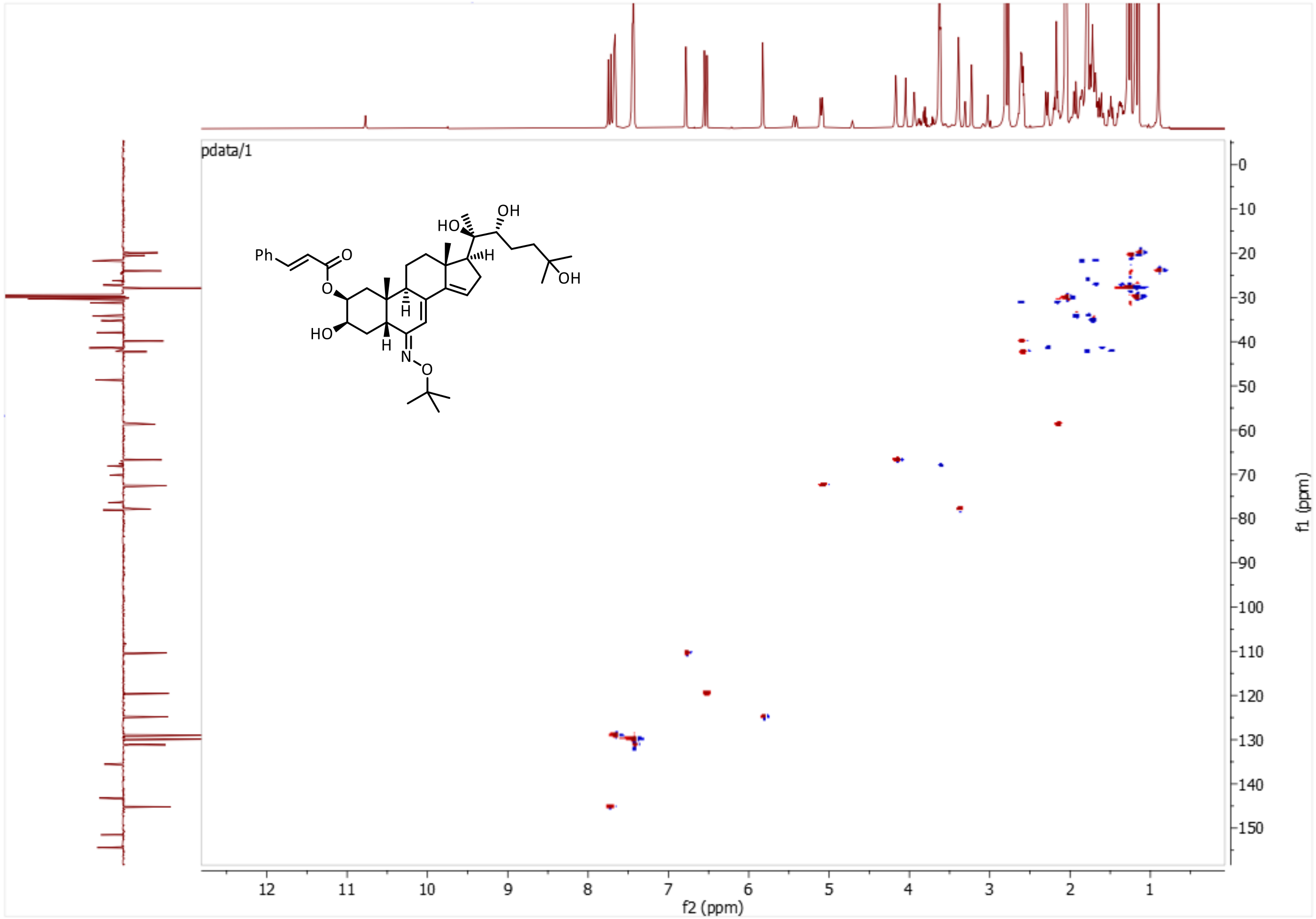

Figure S46. HSQC NMR (acetone-d_6) spectrum of compound **44**
Stachysterone B 6-*O*-*tert*-butyl oxime ether 2-cinnamate (*E* isomer)

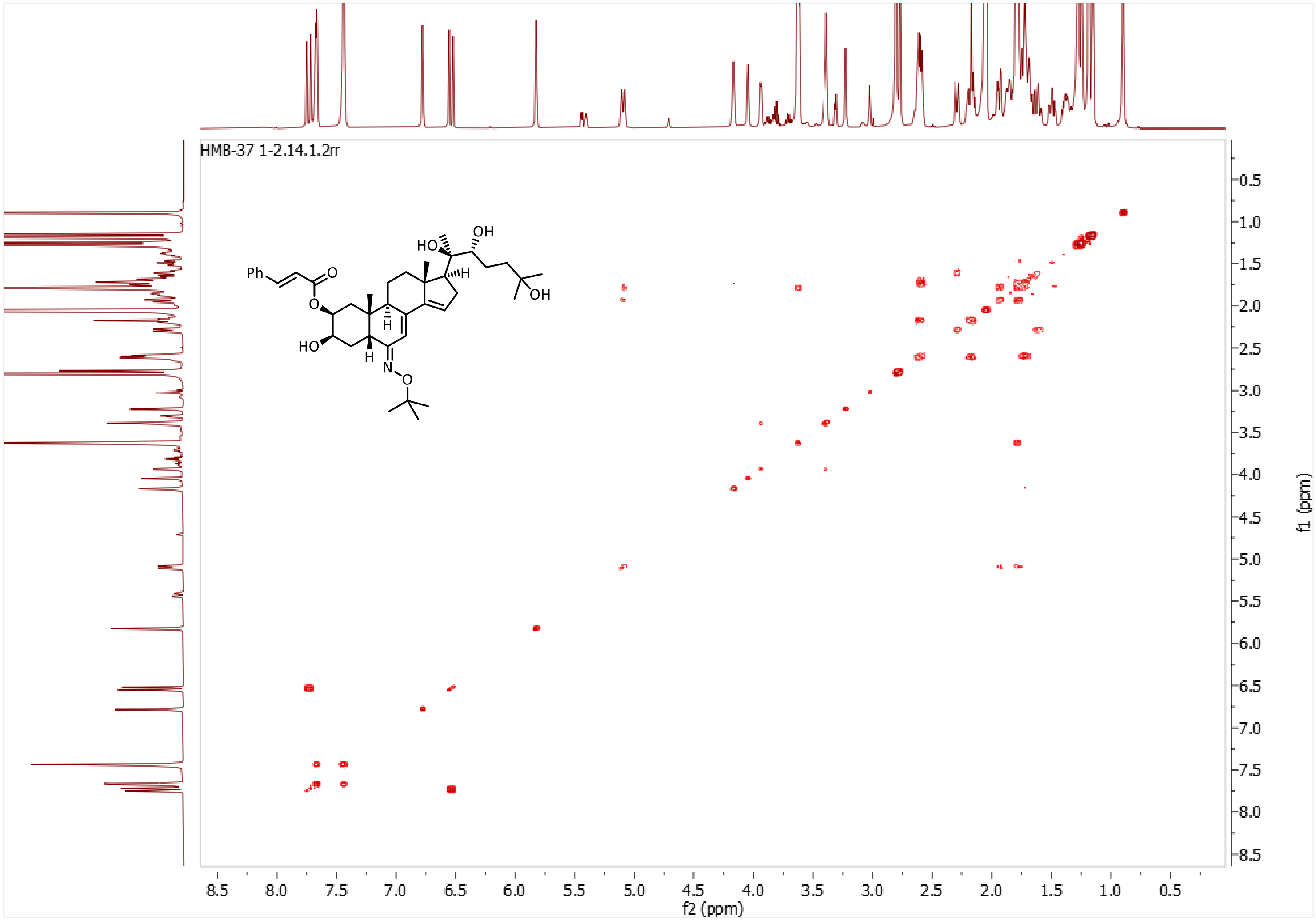

Figure S47. ^1H-^1H COSY NMR (acetone-d_6) spectrum of compound **44** Stachysterone B-6 *O*-*tert*-butyl oxime ether 2-cinnamate (*E* isomer)

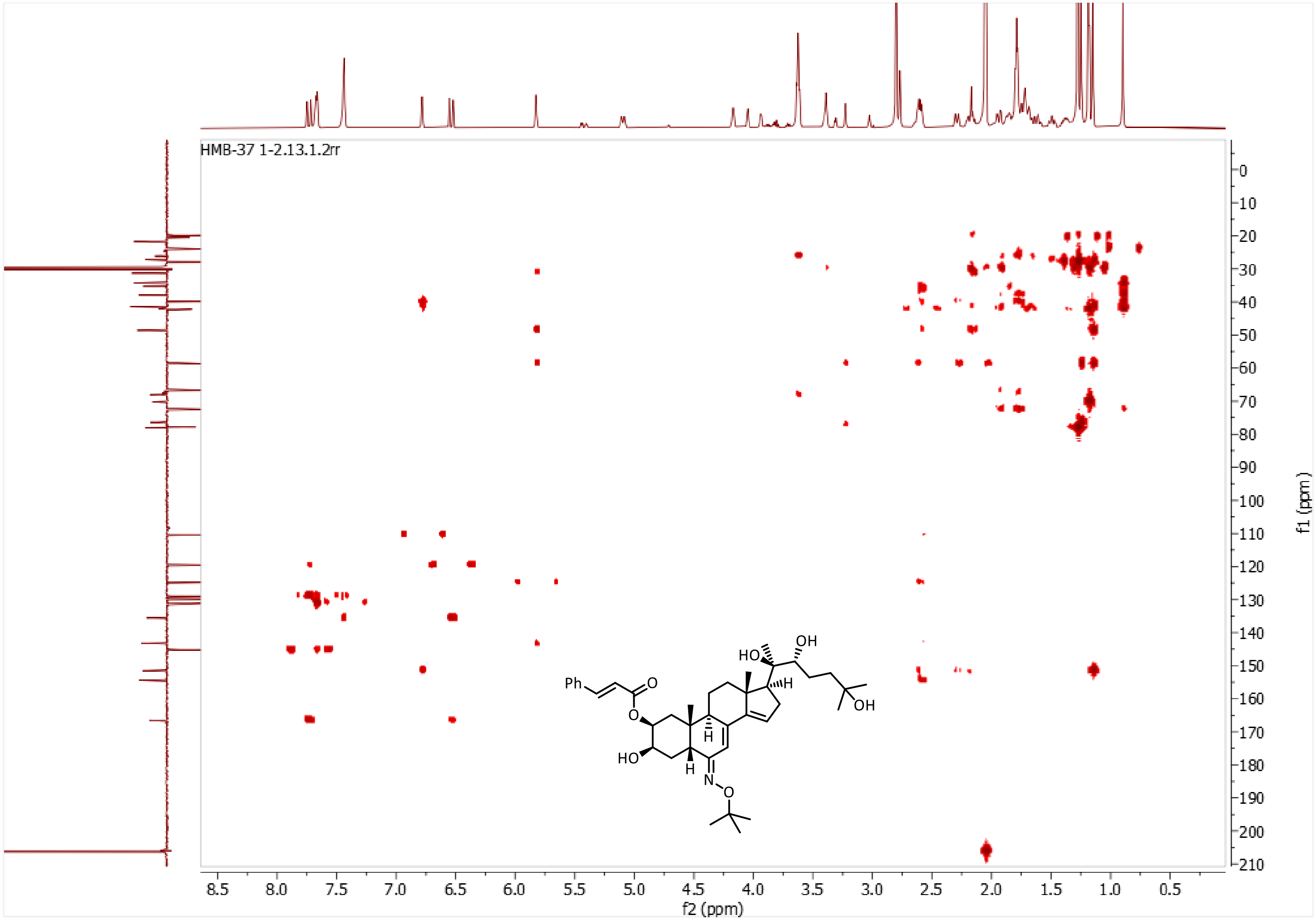

Figure S48. HMBC NMR (acetone-d_6) spectrum of compound **44**
Stachysterone B 6-*O*-*tert*-butyl oxime ether 2-cinnamate (*E* isomer)

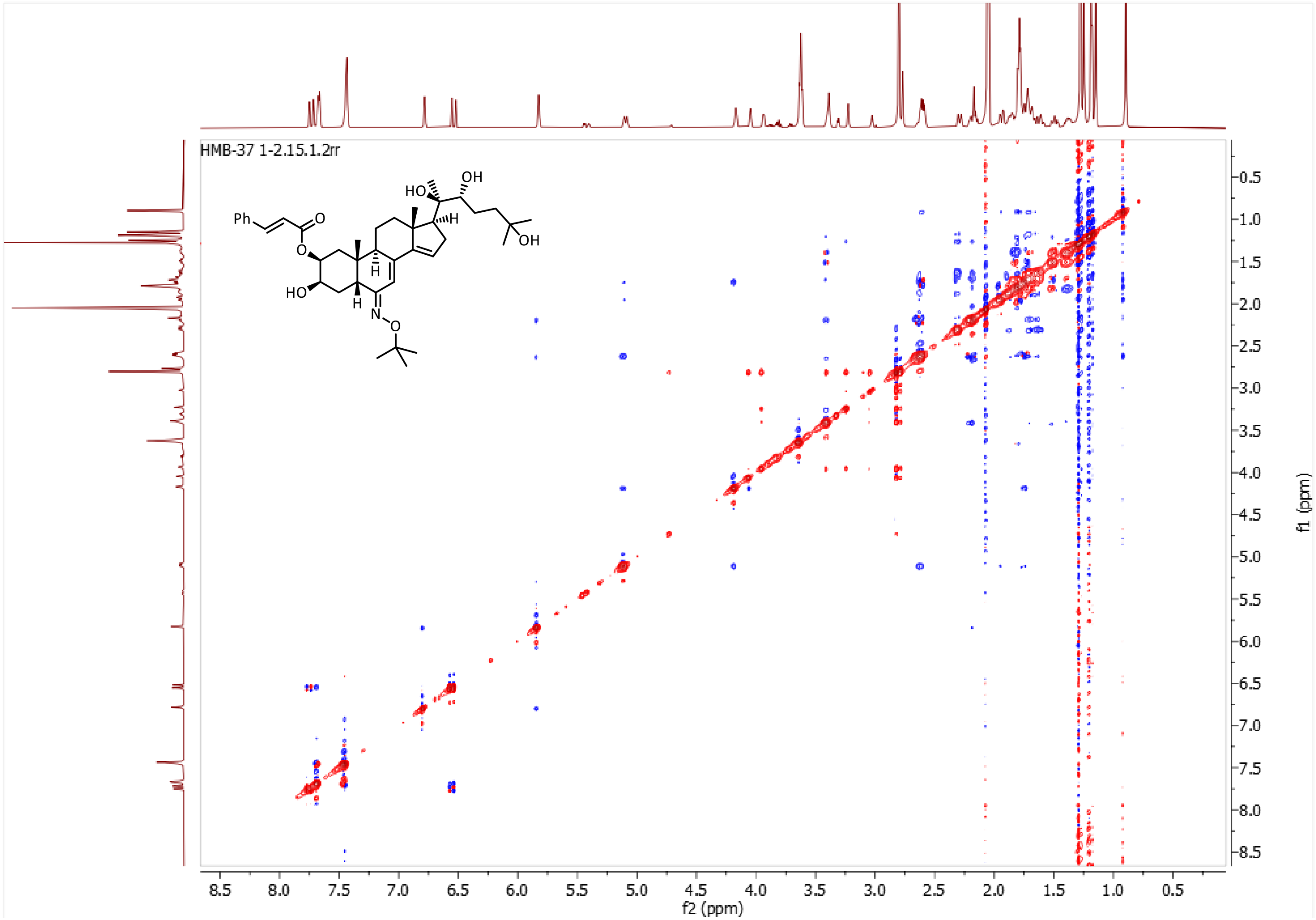

Figure S49. ROESY NMR (acetone-d_6) spectrum of compound **44**
Stachysterone B 6-*O*-*tert*-butyl oxime ether 2-cinnamate (*E* isomer)

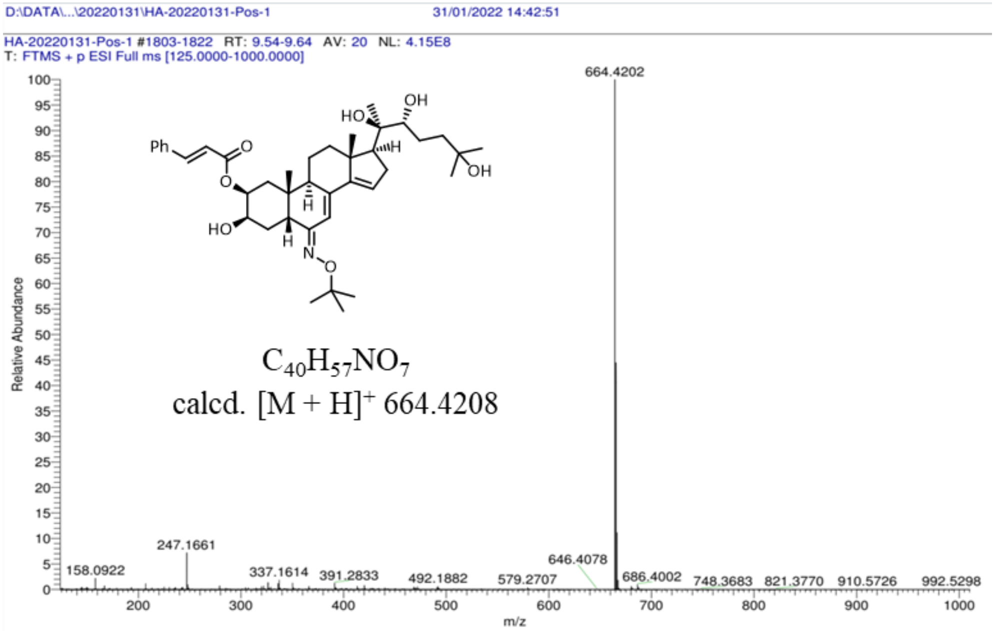

Figure S50. HR-MS spectrum of compound **44**

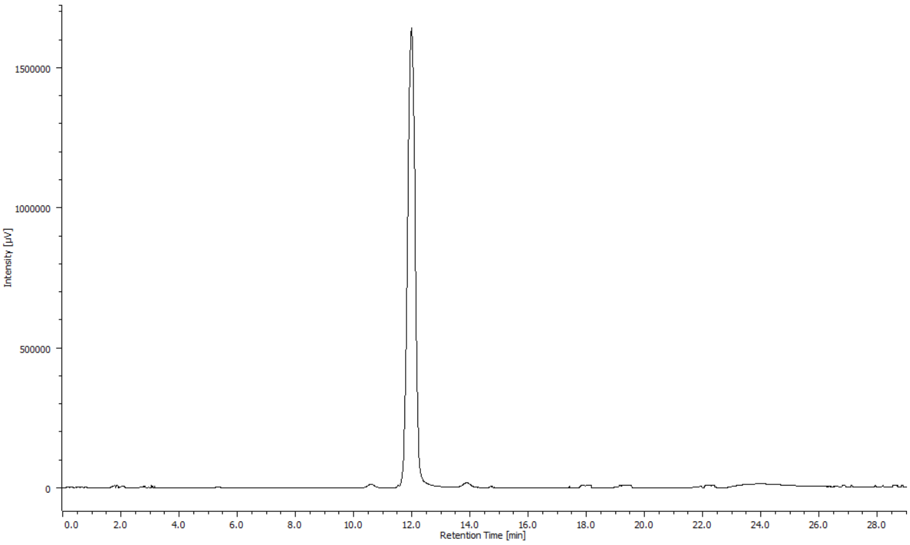

Figure S51. HPLC chromatogram of compound **44**
at its UV absorbance maximum(λ=278.8 nm) Purity 98.0 %.
Column: Kinetex®, 5µm, XB-C18, 100 Å, 250 × 4.6 mm (Phenomenex Inc.); Elution: H_2O:CH_3CN (A:B) 65 % B.

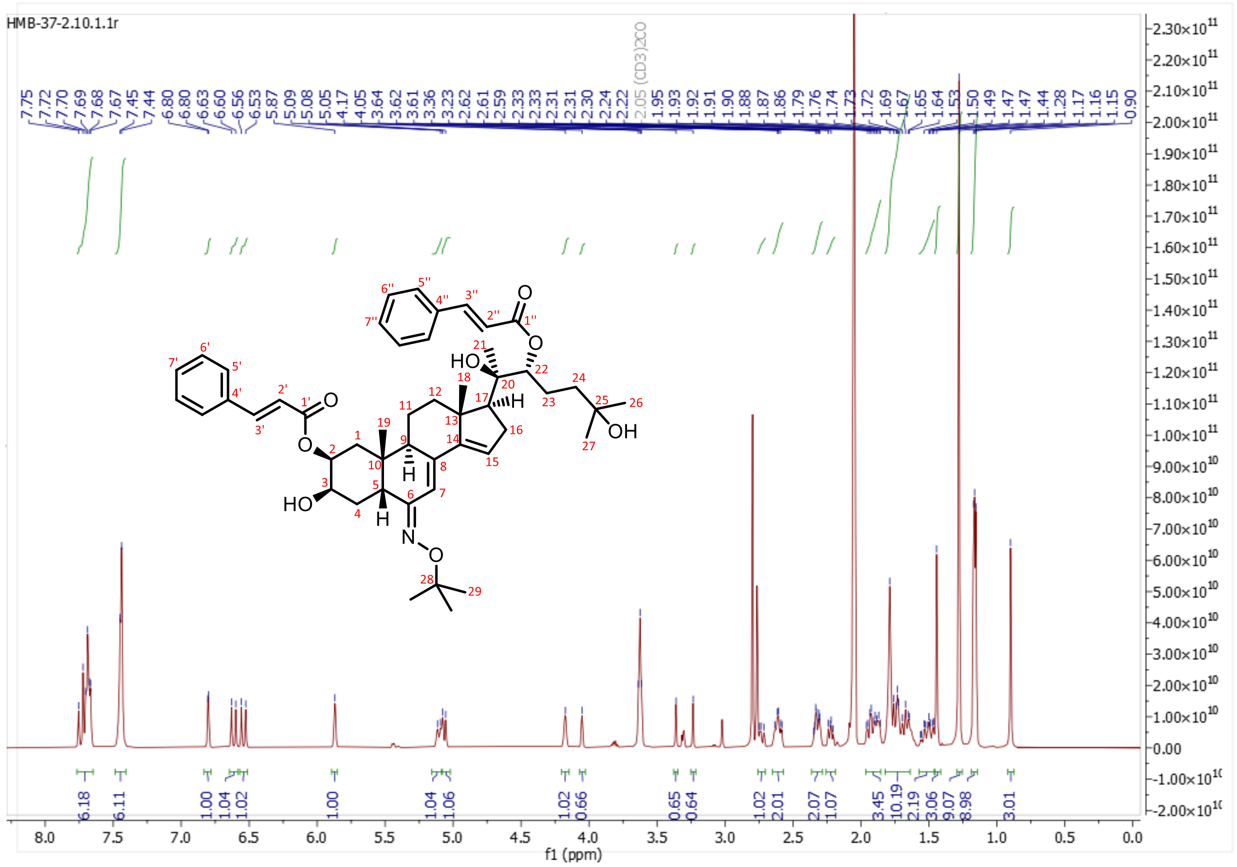

Figure S52. ^{1}H NMR (500 MHz, acetone-d_6) spectrum of compound **45**
Stachysterone B-6 *O*-*tert*-butyl oxime ether 2,22-dicinnamate (*E* isomer). The NMR sample contained some tetrahydrofuran as well (overlapping at around 1.79 ppm): ^{1}H NMR δ 3.63 (t, J = 6.1 Hz), 1.80 – 1.77 (m).

^{1}H NMR (500 MHz, acetone-d_6) δ 7.77 – 7.65 (m, 6H, H-3′, H-5′, H-3″ and H-5″), 7.48 – 7.41 (m, 6H, H-6′, H-7′, H-6″ and H-7″), 6.80 (d, J = 2.7 Hz, 1H, H-7), 6.61 (d, J = 16.0 Hz, 1H, H-2″), 6.54 (d, J = 16.0 Hz, 1H, H-2′), 5.87 (s, 1H, H-15), 5.10 (d, J = 12.0 Hz, 1H, H-2), 5.07 (d, J = 10.8 Hz, 1H, H-22), 4.17 (s, 1H, H-3), 4.05 (s, 1H, 3-OH), 3.36 (s, 1H, 20-OH), 3.23 (s, 1H, 25-OH), 2.76 – 2.70 (m, 1H, H-16), 2.65 – 2.57 (m, 2H, H-5 and H-9), 2.36 – 2.28 (m, 2H, H-12 and H-16), 2.26 – 2.19 (m, 1H, H-17), 1.98 – 1.84 (m, 3H, H-1, H-11 and H-23), 1.82 – 1.62 (m, 6H, H-1, H-4, H-11, H-12 and H-23), 1.57 – 1.46 (m, 2H, H-24), 1.44 (s, 3H, H-21), 1.28 (s, 9H, H-29), 1.17 (s, 3H, H-27), 1.16 (s, 3H, H-18), 1.15 (s, 3H, H-26), 0.90 (s, 3H, H-19).

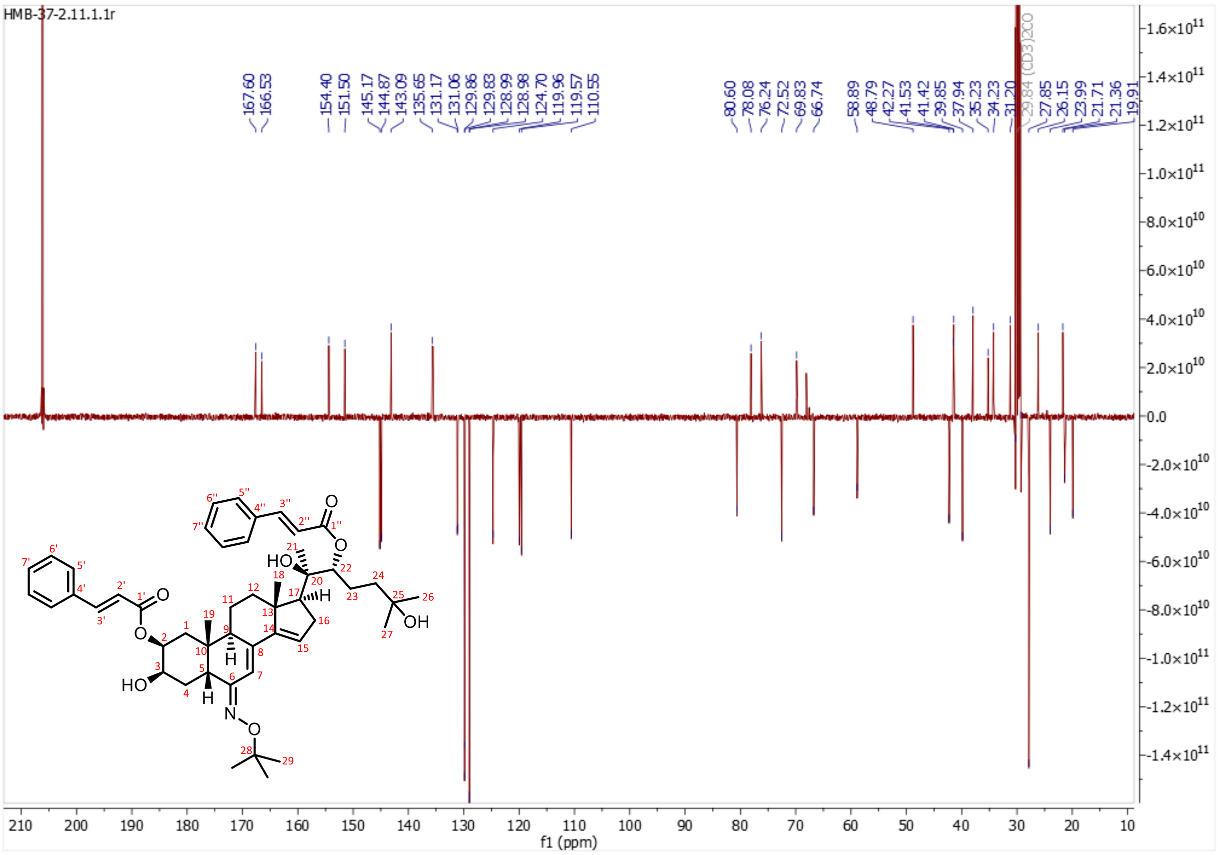

Figure S53. JMOD NMR (126 MHz, acetone-d_6) spectrum of compound **45**
The NMR sample contained some tetrahydrofuran as well: ^{13}C NMR δ 68.1, 26.

^{13}C NMR (126 MHz, acetone-d_6) *δ* 167.6 (C-1″), 166.5 (C-1′), 154.4 (C-6), 151.5 (C-14), 145.2 (C-3′), 144.9 (C-3″), 143.1 (C-8), 135.7 (C-4′ and C-4″), 131.2 (C-7″), 131.1 (C-7′), 129.9 (C-6″), 129.8 (C-6′), 129.0 (C-5″), 129.0 (C-5′), 124.7 (C-15), 120.0 (C-2″), 119.6 (C-2′), 110.6 (C-7), 80.6 (C-22), 78.1 (C-28), 76.2 (C-20), 72.5 (C-2), 69.8 (C-25), 66.7 (C-3), 58.9 (C-17), 48.8 (C-13), 42.3 (C-5), 41.5 (C-24), 41.4 (C-12), 39.8 (C-9), 37.9 (C-10), 35.2 (C-4), 34.2 (C-1), 31.2 (C-16), 30.3 (C-27), 29.3 (C-26), 27.9 (C-29), 26.2 (C-23), 24.0 (C-19), 21.7 (C-11), 21.4 (C-21), 19.9 (C-18).

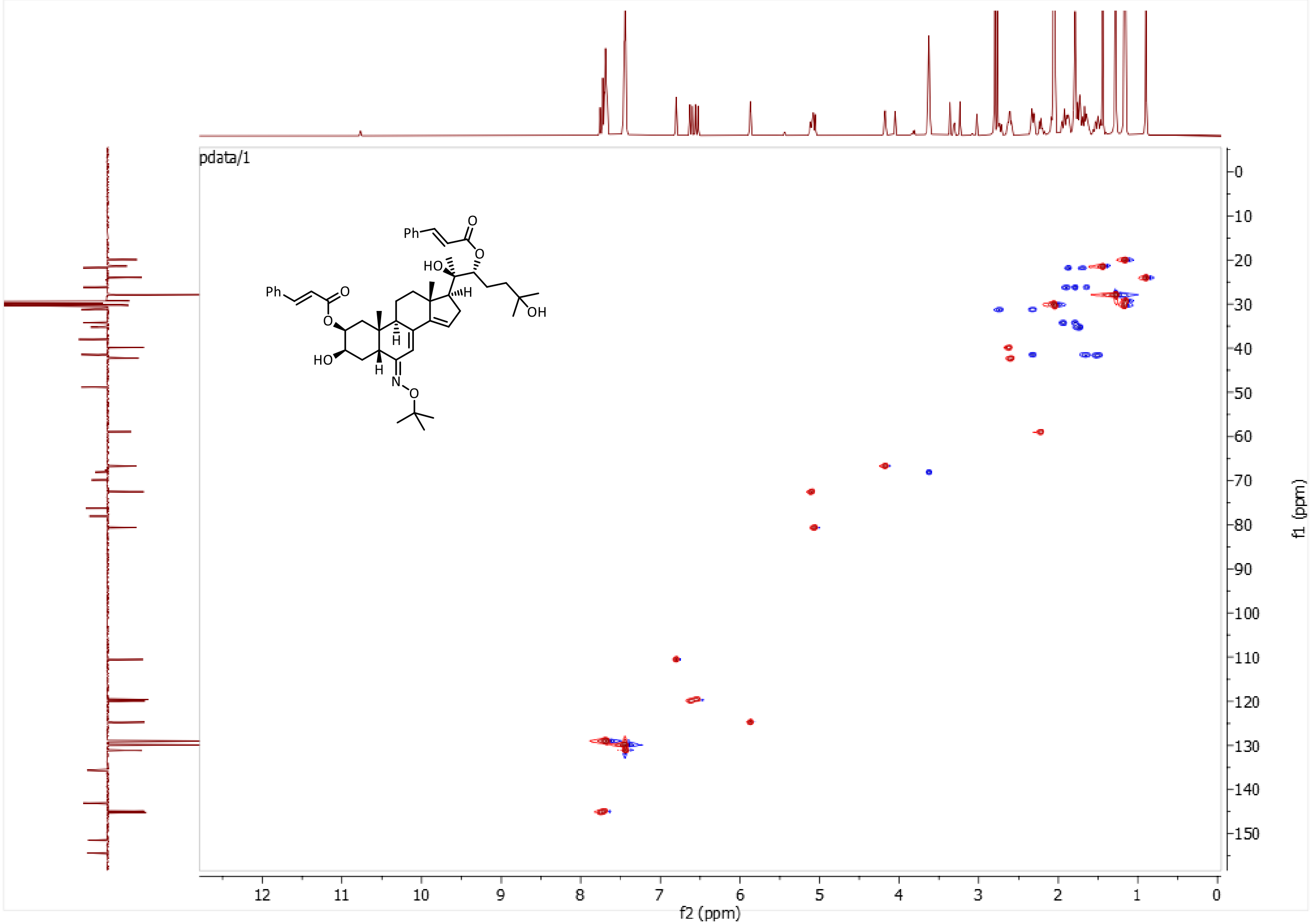

Figure S54. HSQC NMR (acetone-d_6) spectrum of compound **45**
Stachysterone B 6-*O*-*tert*-butyl oxime ether 2,22-dicinnamate (*E* isomer)

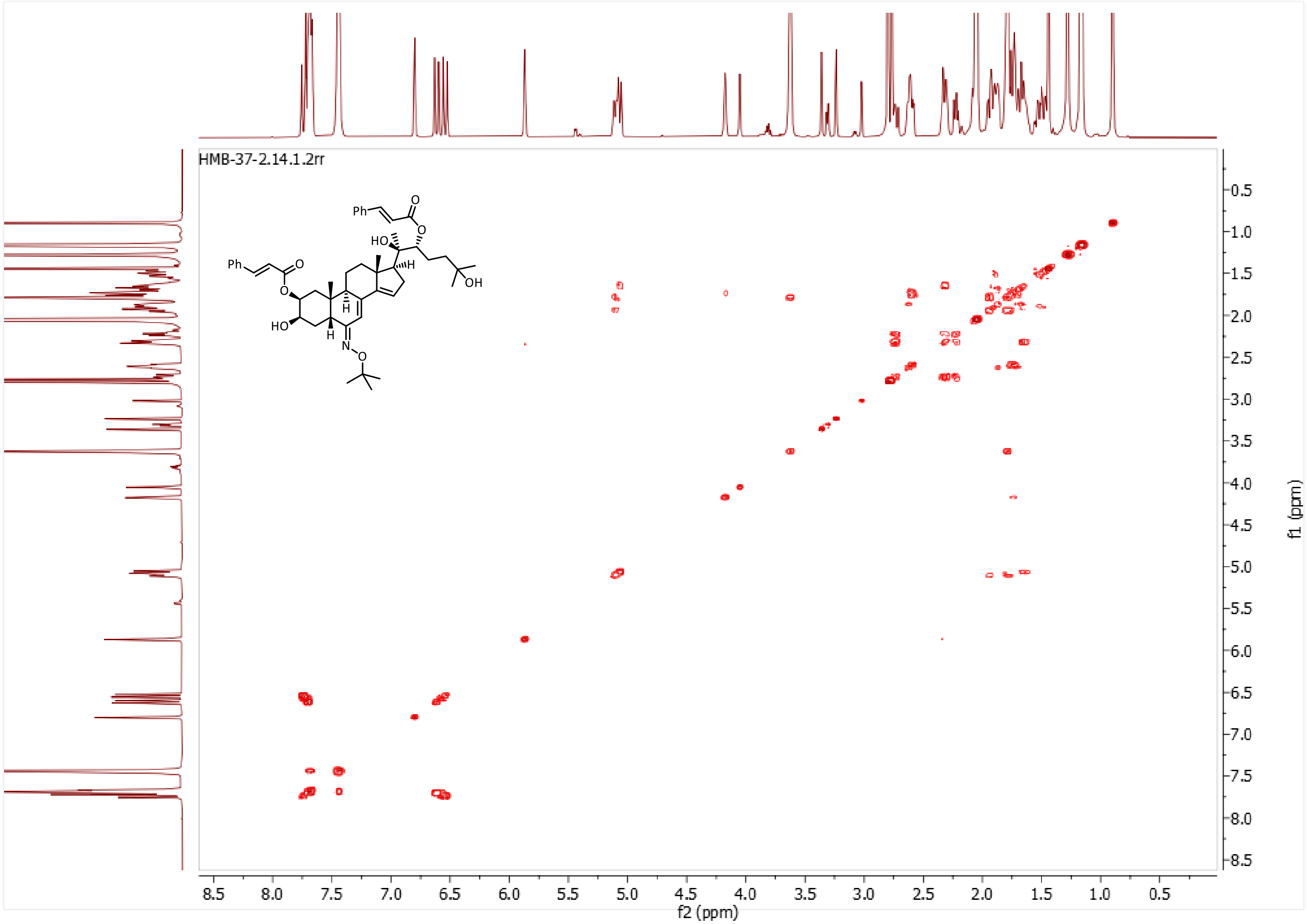

Figure S55. ^{1}H-^{1}H COSY NMR (acetone-d_6) spectrum of compound **45**
Stachysterone B 6-*O*-*tert*-butyl oxime ether 2,22-dicinnamate (*E* isomer)

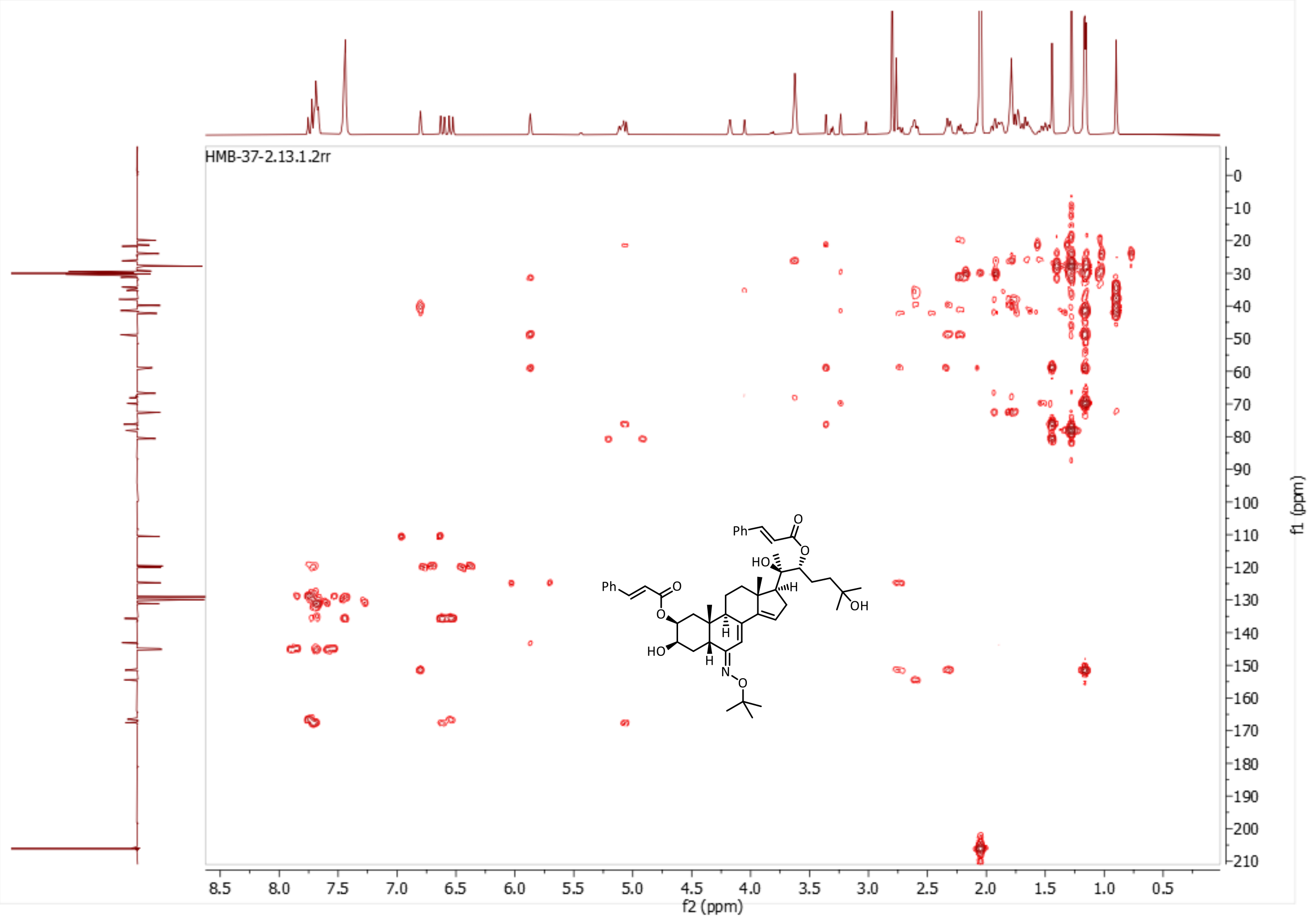

Figure S56. HMBC NMR (acetone-d_6) spectrum of compound **45**
Stachysterone B 6-*O*-*tert*-butyl oxime ether 2,22-dicinnamate (*E* isomer)

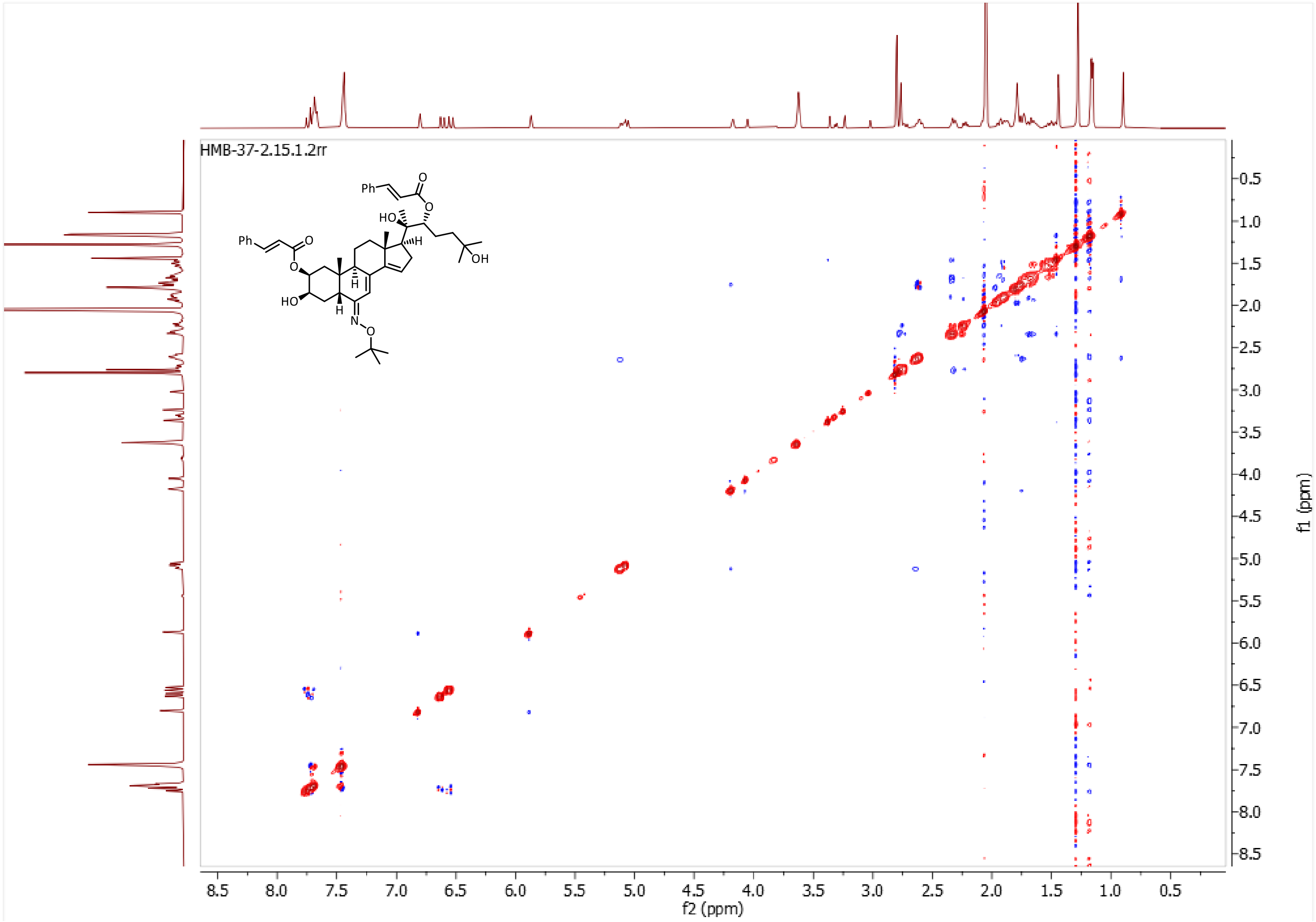

Figure S57. ROESY NMR (acetone-d_6) spectrum of compound **45**
Stachysterone B 6-*O*-*tert*-butyl oxime ether 2,22-dicinnamate (*E* isomer)

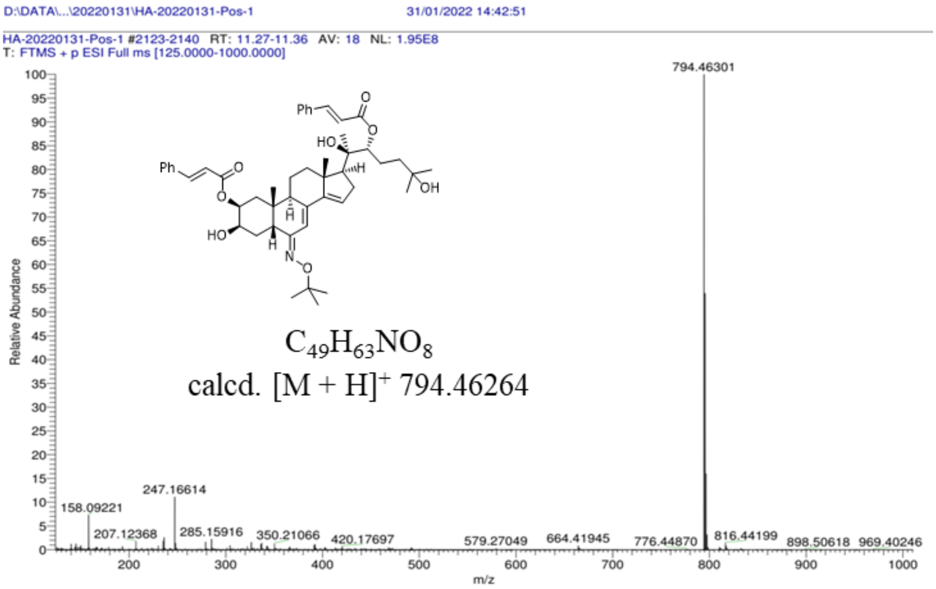

Figure S58. HR-MS spectrum of compound **45**

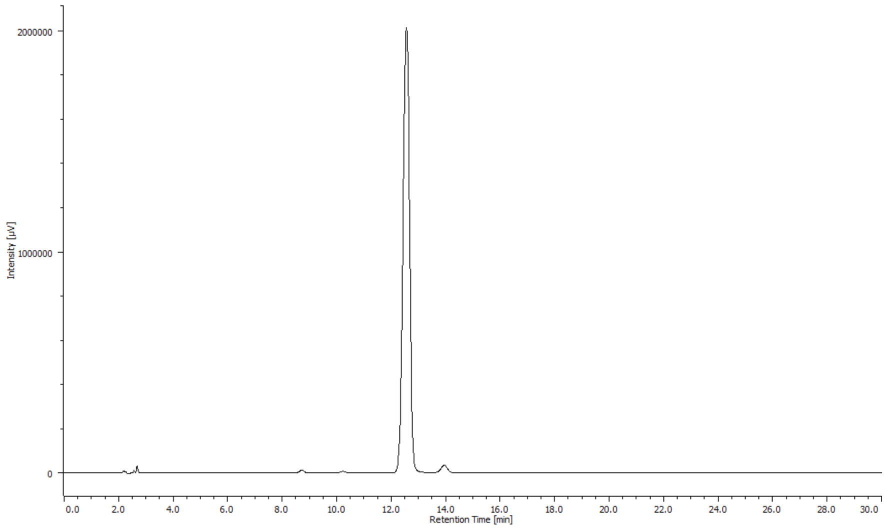

Figure S59. HPLC chromatogram of compound **45** at its UV absorbance maximum (λ=275.0 nm). Purity 96.6 %.
Column: Kinetex®, 5µm, XB-C18, 100 Å, 250 × 4.6 mm (Phenomenex Inc.); Elution H_2O:CH_3CN (A:B) 80 % B.

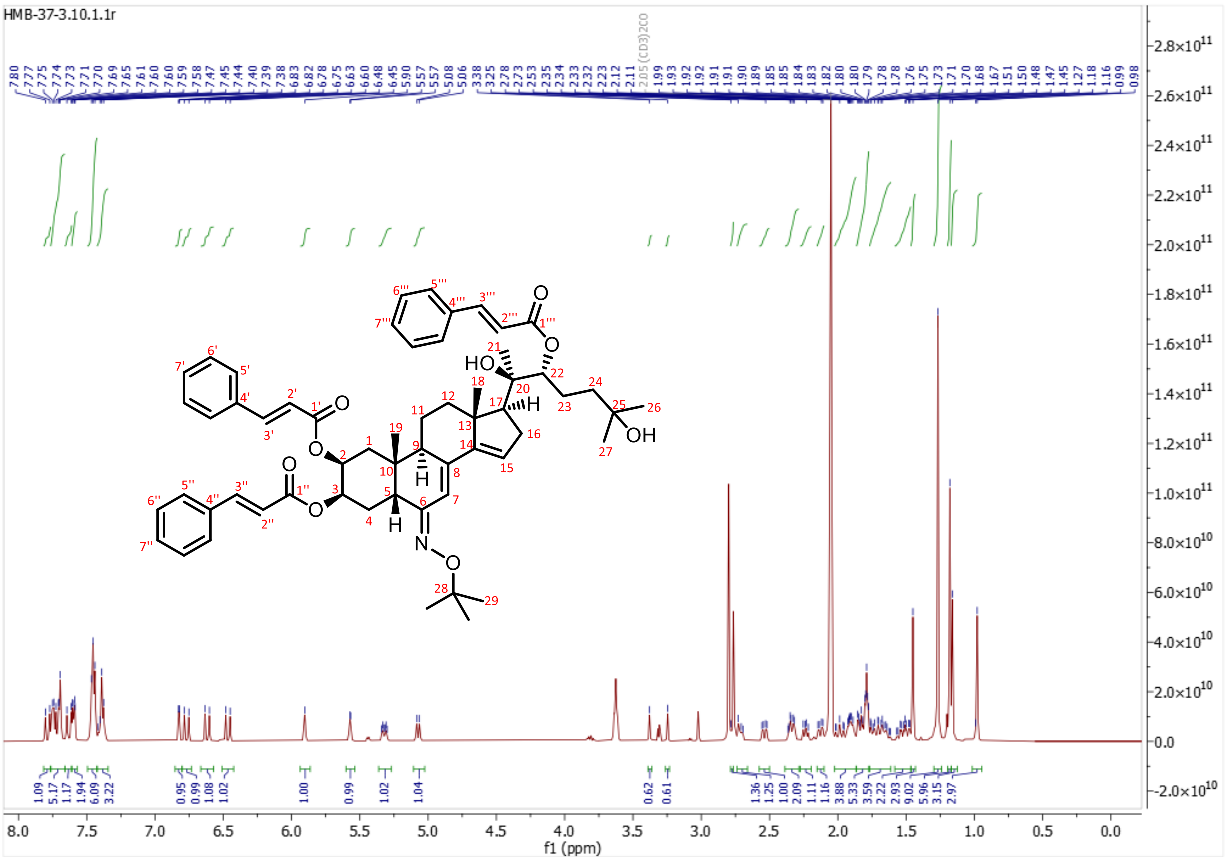

Figure S60. ^{1}H NMR (500 MHz, acetone-d_6) of compound **46**
Stachysterone B 6-*O*-*tert*-butyl oxime ether 2,3,22-tricinnamate (*E* isomer) The NMR sample contained some tetrahydrofuran as well (overlapping at around 1.79 ppm): ^{1}H NMR δ 3.63 (t, J = 6.1 Hz), 1.80 – 1.77 (m).

^{1}H NMR (500 MHz, acetone-d_6) δ 7.79 (d, J = 16.0 Hz, 1H, H-3″), 7.77 – 7.66 (m, 5H, H-5″, H-3‴ and H-5‴), 7.63 (d, J = 16.0 Hz, 1H, H-3′), 7.61 – 7.57 (m, 2H, H-5′), 7.49 – 7.42 (m, 6H, H-6″, H-7″, H-6‴ and H-7‴), 7.42 – 7.34 (m, 3H, H-6′, H-7′), 6.82 (d, J = 2.9 Hz, 1H, H-7), 6.77 (d, J = 16.0 Hz, 1H, H-2″), 6.62 (d, J = 16.0 Hz, 1H, H-2‴), 6.47 (d, J = 16.0 Hz, 1H, H-2′), 5.90 (s, 1H, H-15), 5.57 (d, J = 3.3 Hz, 1H, H-3), 5.35 – 5.29 (m, 1H, H-2), 5.07 (d, J = 10.4 Hz, 1H, H-22), 3.38 (s, 1H, 20-OH), 3.25 (s, 1H, 25-OH), 2.79 – 2.76 (m, 1H, H-16), 2.74 – 2.66 (m, 1H, H-9), 2.54 (dd, J = 13.4, 4.3 Hz, 1H, H-5), 2.39 – 2.29 (m, 2H, H-12 and H-16), 2.28 – 2.19 (m, 1H, H-17), 2.15 – 2.10 (m, 1H, H-1), 2.02 – 1.95 (m, 1H, H-4), 1.95 – 1.87 (m, 2H, H-11 and H-23), 1.86 – 1.77 (m, 2H, H-1 and H-4), 1.77 – 1.61 (m, 3H, H-11, H-12 and H-23), 1.58 – 1.47 (m, 2H, H-24), 1.45 (s, 3H, H-21), 1.27 (s, 9H, H-29), 1.18 (s, 6H, H-18 and H-27), 1.16 (s, 3H, H-26), 0.98 (s, 3H, H-19).

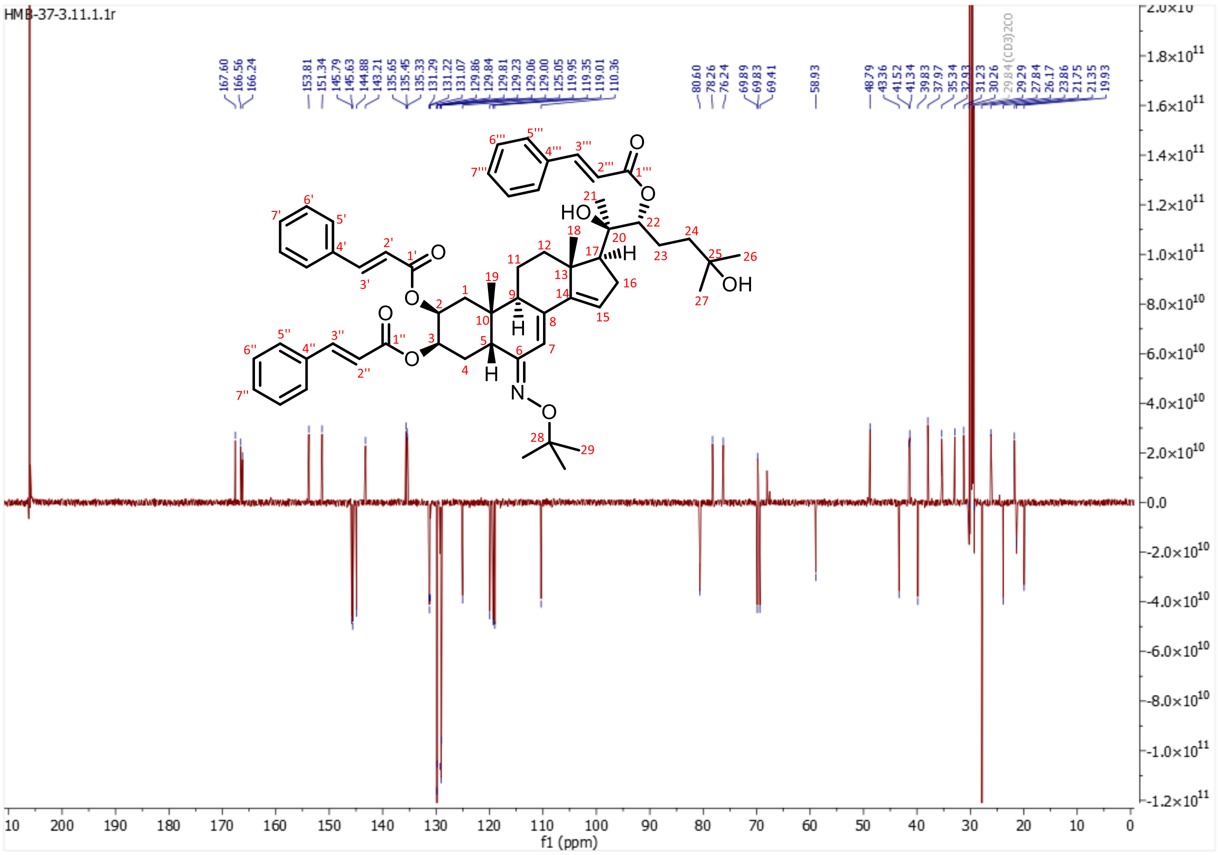

Figure S61. JMOD NMR (126 MHz, acetone-d_6) of compound **46.**
Stachysterone B 6-*O*-*tert*-butyl oxime ether 2,3,22-tricinnamate (*E* isomer)
The NMR sample contained some tetrahydrofuran as well: ^{13}C NMR δ 68.1, 26.

^{13}C NMR (126 MHz, acetone-d_6) δ 167.6 (C-1‴), 166.6 (C-1″), 166.2 (C-1′), 153.8 (C-6), 151.3 (C-14), 145.8 (C-3″), 145.6 (C-3′), 144.9 (C-3‴), 143.2 (C-8), 135.7 (C-4‴), 135.5 (C-4′), 135.3 (C-4″), 131.3 (C-7″), 131.2 (C-7′), 131.1 (C-7‴), 129.9 (C-6‴), 129.8 (C-6″), 129.8 (C-6′), 129.2 (C-5″), 129.1 (C-5′), 129.0 (C-5‴), 125.0 (C-15), 120.0 (C-2‴), 119.4 (C-2″), 119.0 (C-2′), 110.4 (C-7), 80.6 (C-22), 78.3 (C-28), 76.2 (C-20), 69.9 (C-2), 69.8 (C-25), 69.4 (C-3), 58.9 (C-17), 48.8 (C-13), 43.4 (C-5), 41.5 (C-24), 41.3 (C-12), 39.8 (C-9), 38.0 (C-10), 35.3 (C-1), 32.9 (C-4), 31.2 (C-16), 30.3 (C-27), 29.3 (C-26), 27.8 (C-29), 26.2 (C-23), 23.9 (C-19), 21.8 (C-11), 21.4 (C-21), 19.9 (C-18).

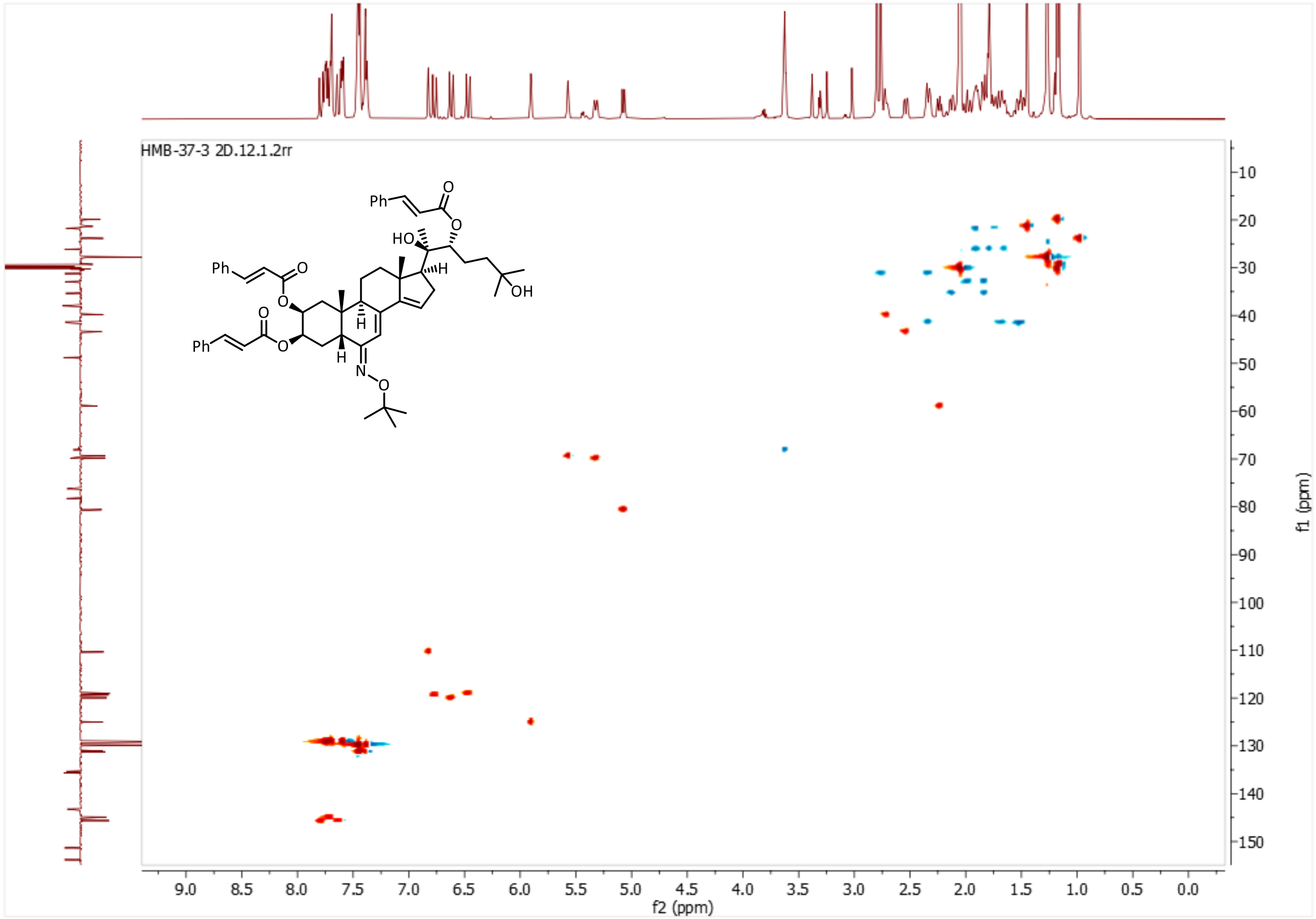

Figure S62. HSQC NMR (acetone-d_6) spectrum of compound **46**
Stachysterone B 6-*O*-*tert*-butyl oxime ether 2,3,22-tricinnamate (*E* isomer)

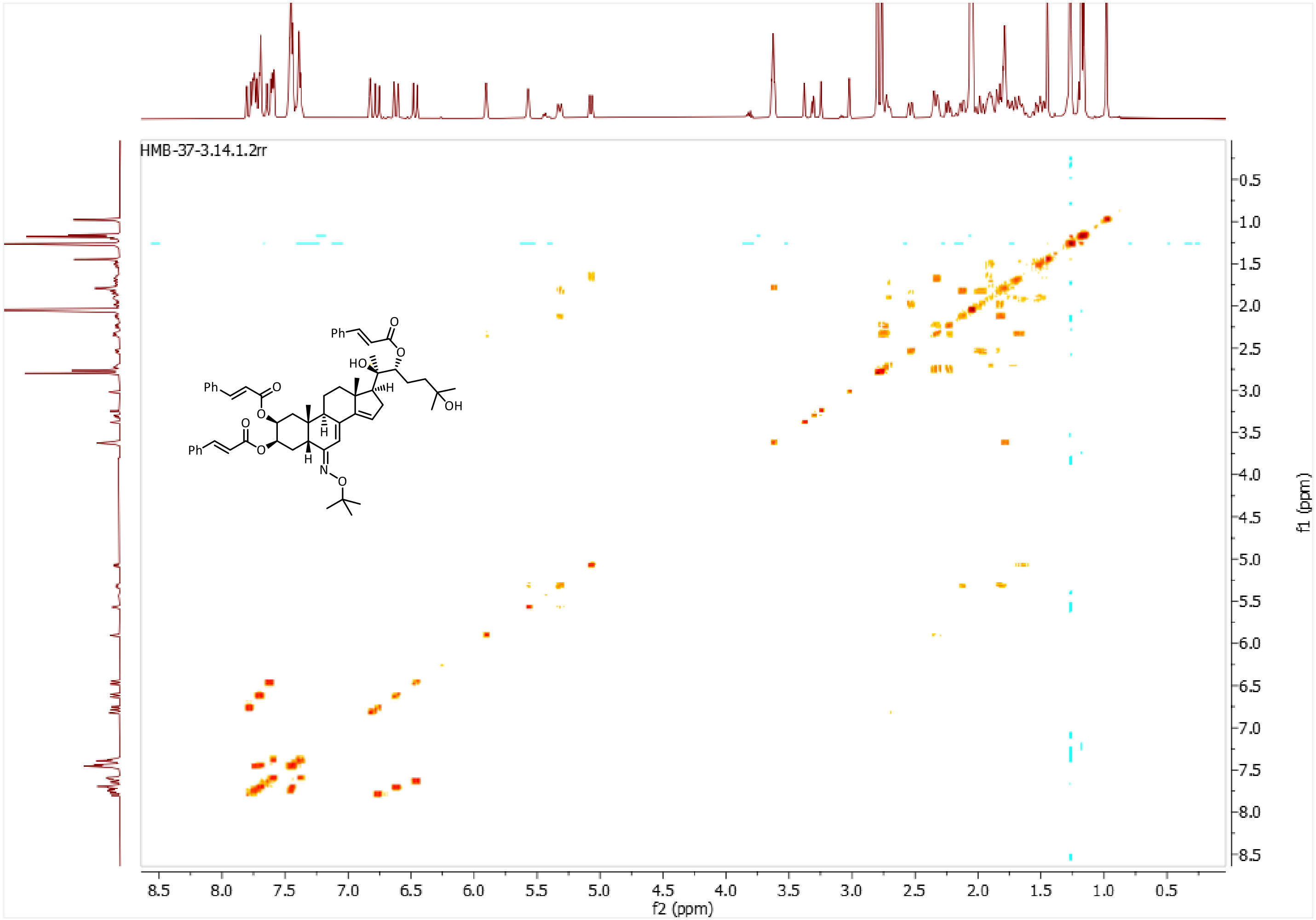

Figure S63. ^{1}H-^{1}H COSY NMR (acetone-d_6) spectrum of compound **46**
Stachysterone B 6-*O*-*tert*-butyl oxime ether 2,3,22-tricinnamate (*E* isomer)

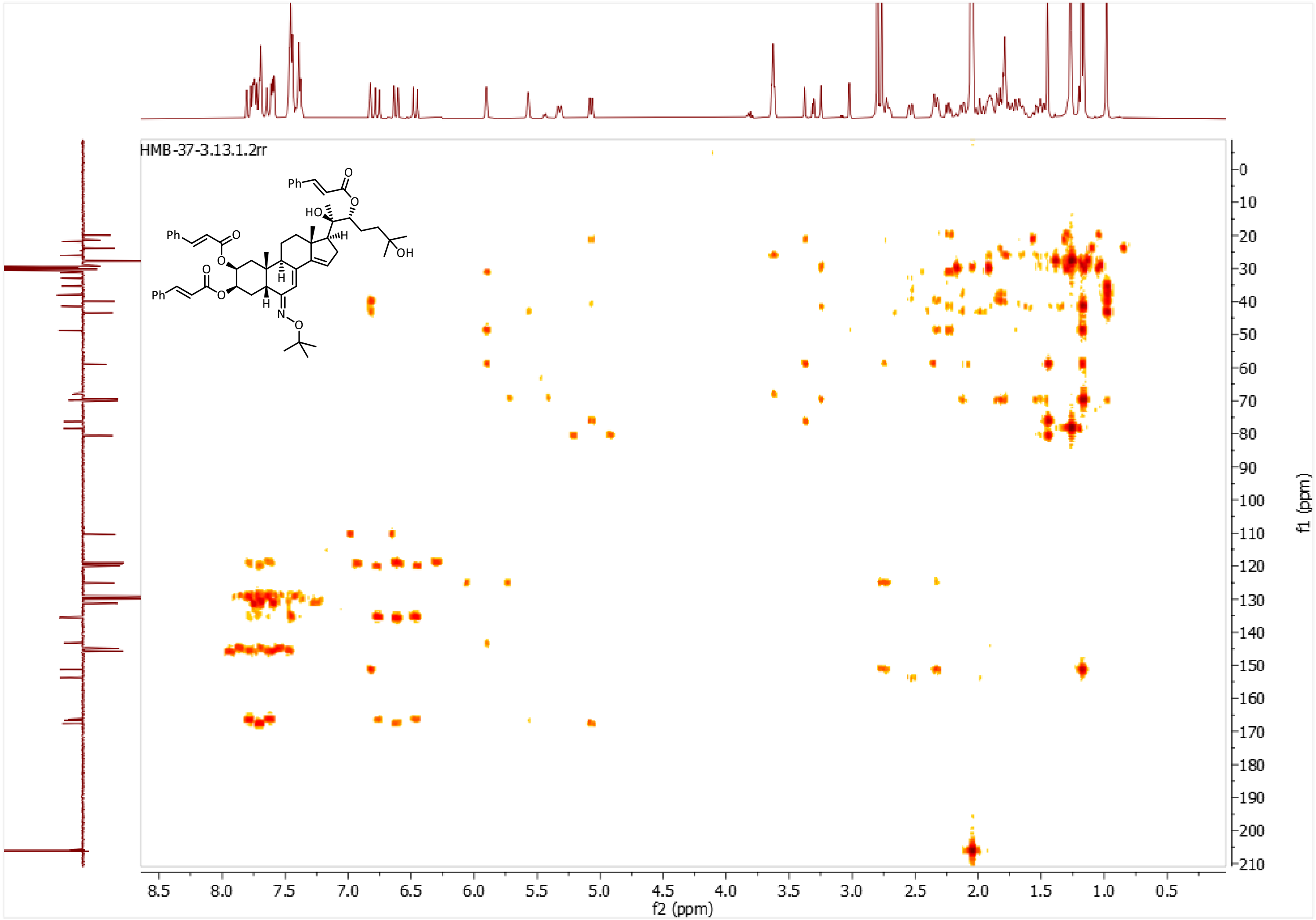

Figure S64. HMBC NMR (acetone-d_6) spectrum of compound **46**
Stachysterone B 6-*O*-*tert*-butyl oxime ether 2,3,22-tricinnamate (*E* isomer)

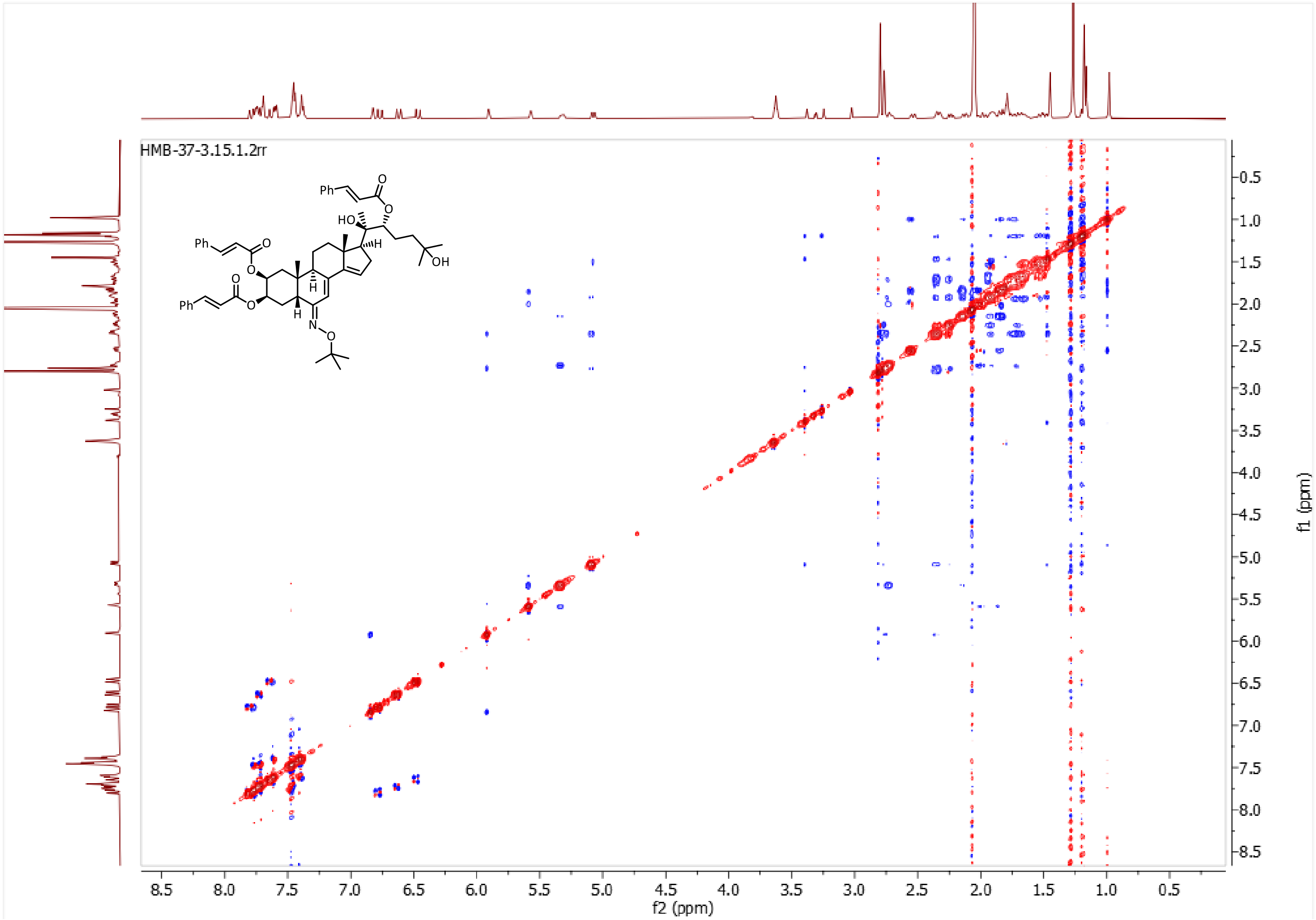

Figure S65. ROESY NMR (acetone-d_6) spectrum of compound **46**
Stachysterone B 6-*O*-*tert*-butyl oxime ether 2,3,22-tricinnamate (*E* isomer)

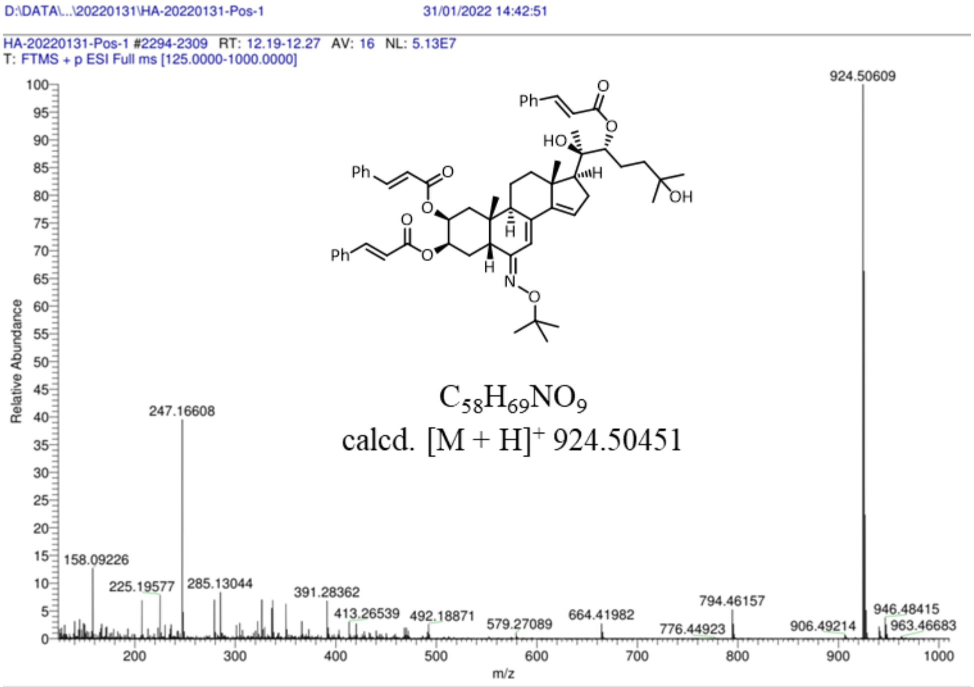

Figure S66. HR-MS spectrum of compound **46**

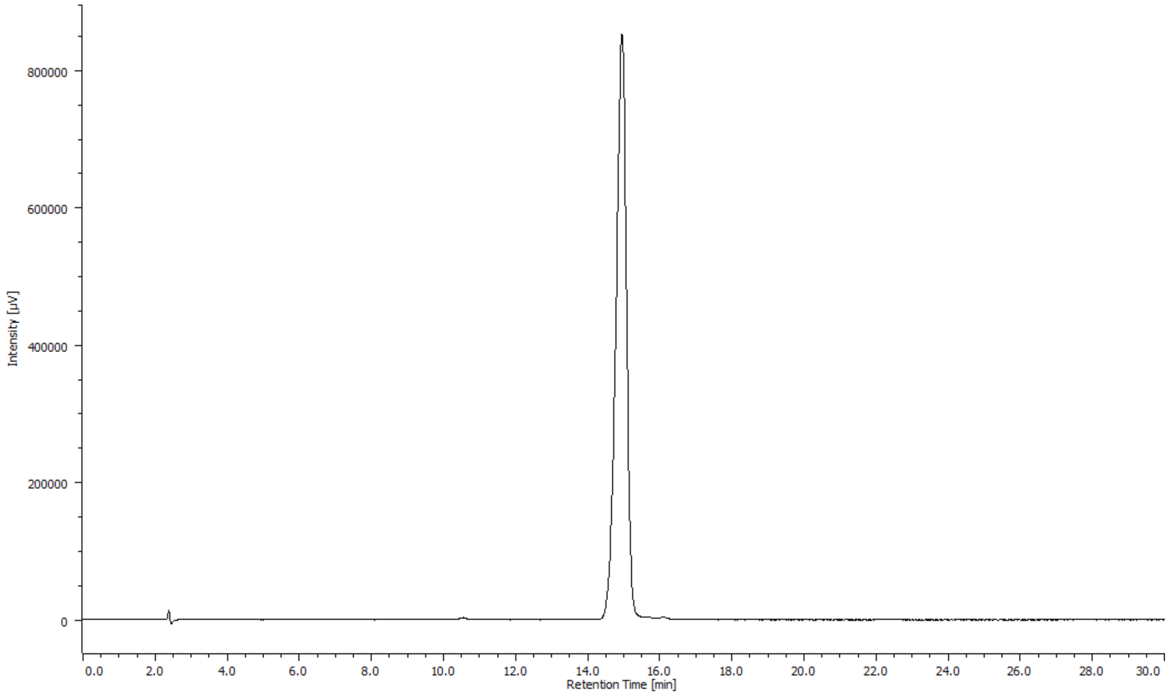

Figure S67. HPLC chromatogram of compound **46** at its UV absorbance maximum (λ=278.7 nm). Purity 98.0 %.
Column: Kinetex®, 5µm, XB-C18, 100 Å, 250 × 4.6 mm (Phenomenex Inc.); Elution: H_2O:CH_3CN (A:B) 90 % B.

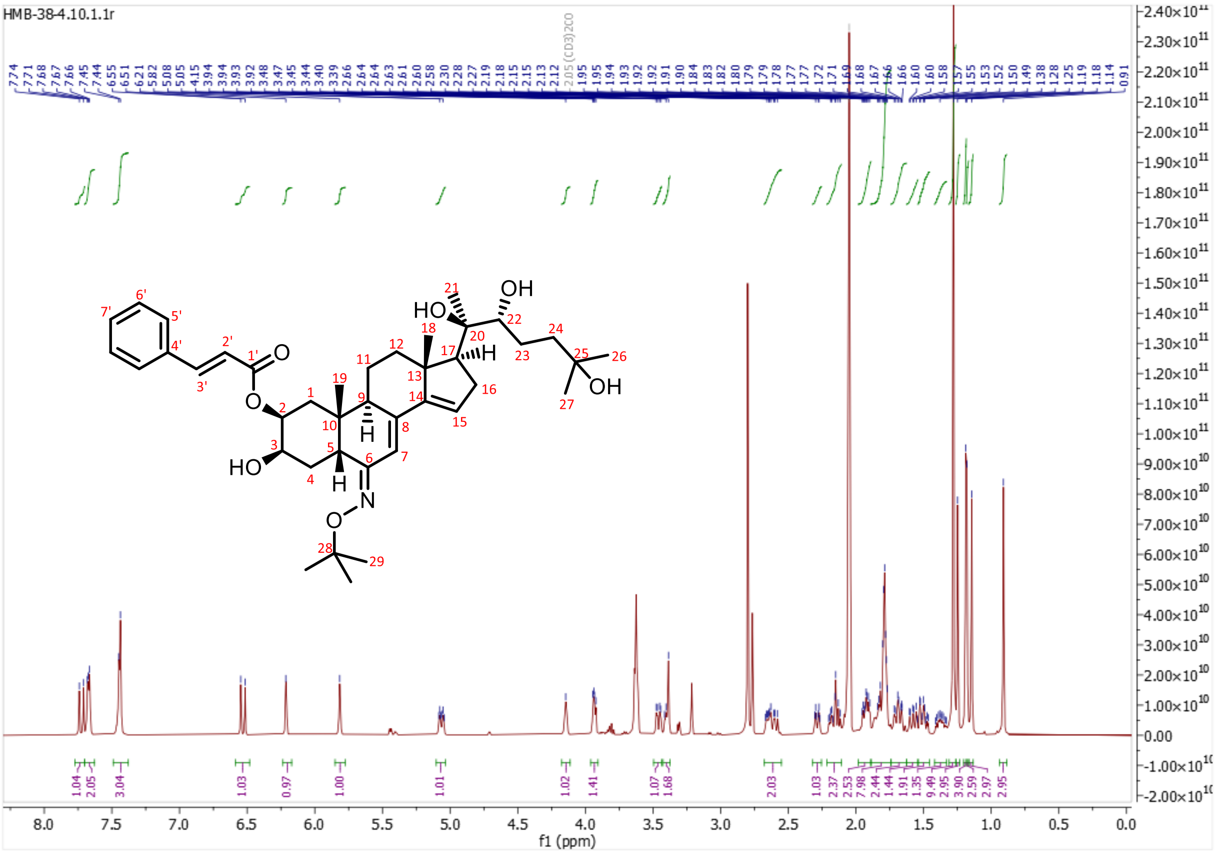

Figure S68. ^{1}H NMR (500 MHz, acetone-d_6) spectrum of compound **47** Stachysterone B 6-*O*-*tert*-butyl oxime ether 2-cinnamate (*Z* isomer). The NMR sample contained some tetrahydrofuran as well (overlapping at around 1.79 ppm): ^{1}H NMR δ 3.63 (t, J = 6.1 Hz), 1.80 – 1.77 (m).

^{1}H NMR (500 MHz, acetone-d_6) δ 7.72 (d, *J* = 16.0 Hz, 1H, H-3′), 7.70 – 7.63 (m, 2H, H-5′), 7.49 – 7.38 (m, 3H, H-6′ and H-7′), 6.53 (d, *J* = 16.0 Hz, 1H, H-2′), 6.21 (s, 1H, H-7), 5.82 (s, 1H, H-15), 5.06 (dt, *J* = 12.4, 3.6 Hz, 1H, H-2), 4.15 (s, 1H, H-3), 3.96 – 3.91 (m, 2H, 3-OH and 22-OH), 3.46 (dd, *J* = 12.9, 4.0 Hz, 1H, H-5), 3.43 – 3.37 (m, 2H, 20-OH and H-22), 2.68 – 2.55 (m, 2H, H-9 and H-16), 2.32 – 2.26 (m, 1H, H-12), 2.21 – 2.11 (m, 2H, H-16 and H-17), 1.98 – 1.89 (m, 2H, H-1 and H-4), 1.89 – 1.74 (m, 3H, H-1, H-11 and H-24), 1.74 – 1.62 (m, 2H, H-11 and H-23), 1.62 – 1.54 (m, 1H, H-12), 1.54 – 1.46 (m, 2H, H-4 and H-24), 1.42 – 1.33 (m, 1H, H-23), 1.28 (s, 9H, H-29), 1.25 (s, 3H, H-21), 1.19 (s, 3H, H-27), 1.18 (s, 3H, H-26), 1.14 (s, 3H, H-18), 0.91 (s, 3H, H-19).

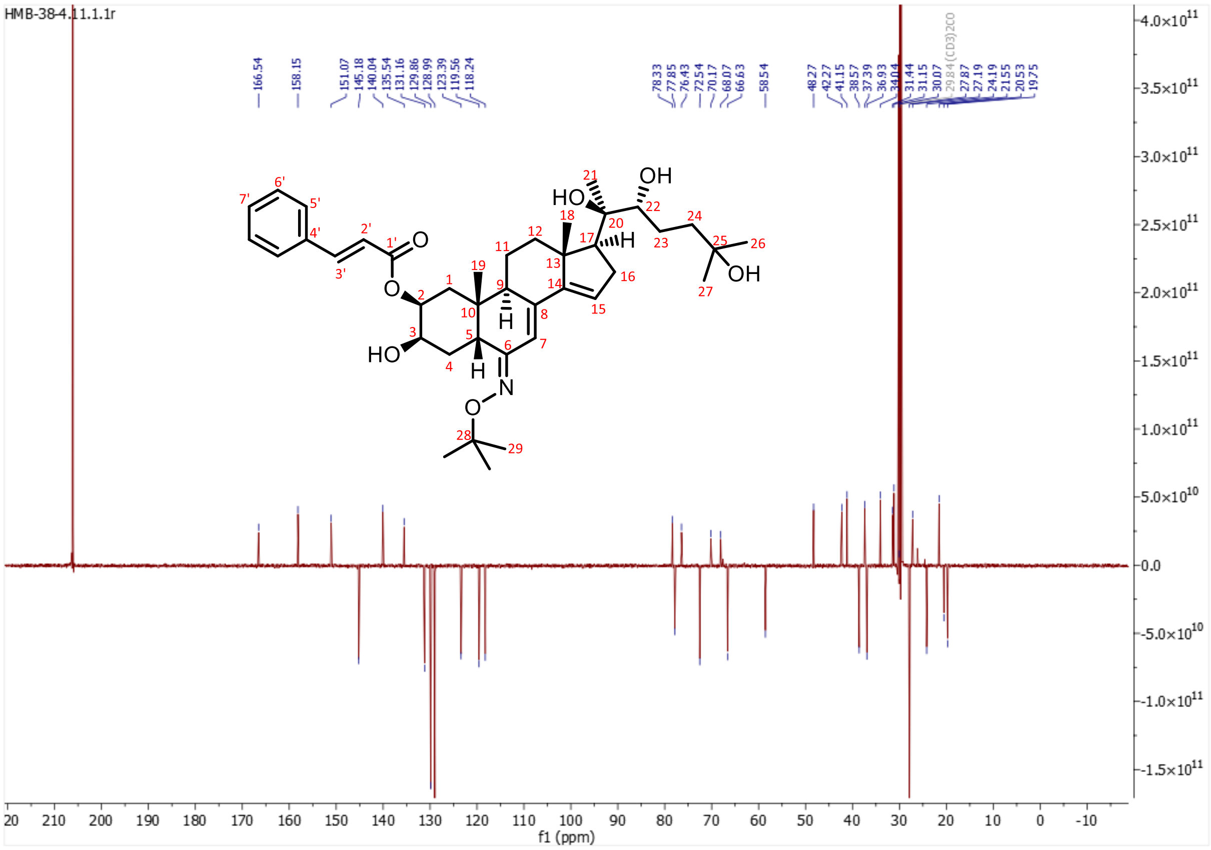

Figure S69. JMOD NMR (126 MHz, acetone-*d*$_6$) spectrum of compound **47**
The NMR sample contained some tetrahydrofuran as well: ^{13}C NMR δ 68.1, 26.

^{13}C NMR (126 MHz, acetone-*d*$_6$) δ 166.5 (C-1′), 158.1 (C-6), 151.1 (C-14), 145.2 (C-3′), 140.0 (C-8), 135.5 (C-4′), 131.2 (C-7′), 129.9 (C-6′), 129.0 (C-5′)123.4 (C-15), 119.6 (C-7), 118.2 (C-2′), 78.3 (C-28), 77.8 (C-22), 76.4 (C-20), 72.5 (C-2), 70.2 (C-25), 66.6 (C-3), 58.5 (C-17), 48.3 (C-13), 42.3 (C-24), 41.2 (C-12), 38.6 (C-9), 37.4 (C-10), 36.9 (C-5), 34.0 (C-1), 31.4 (C-4), 31.2 (C-16), 30.1 (C-26 and C-27), 27.9 (C-29), 27.2 (C-23), 24.2 (C-19), 21.5 (C-11), 20.5 (C-21), 19.7 (C-18).

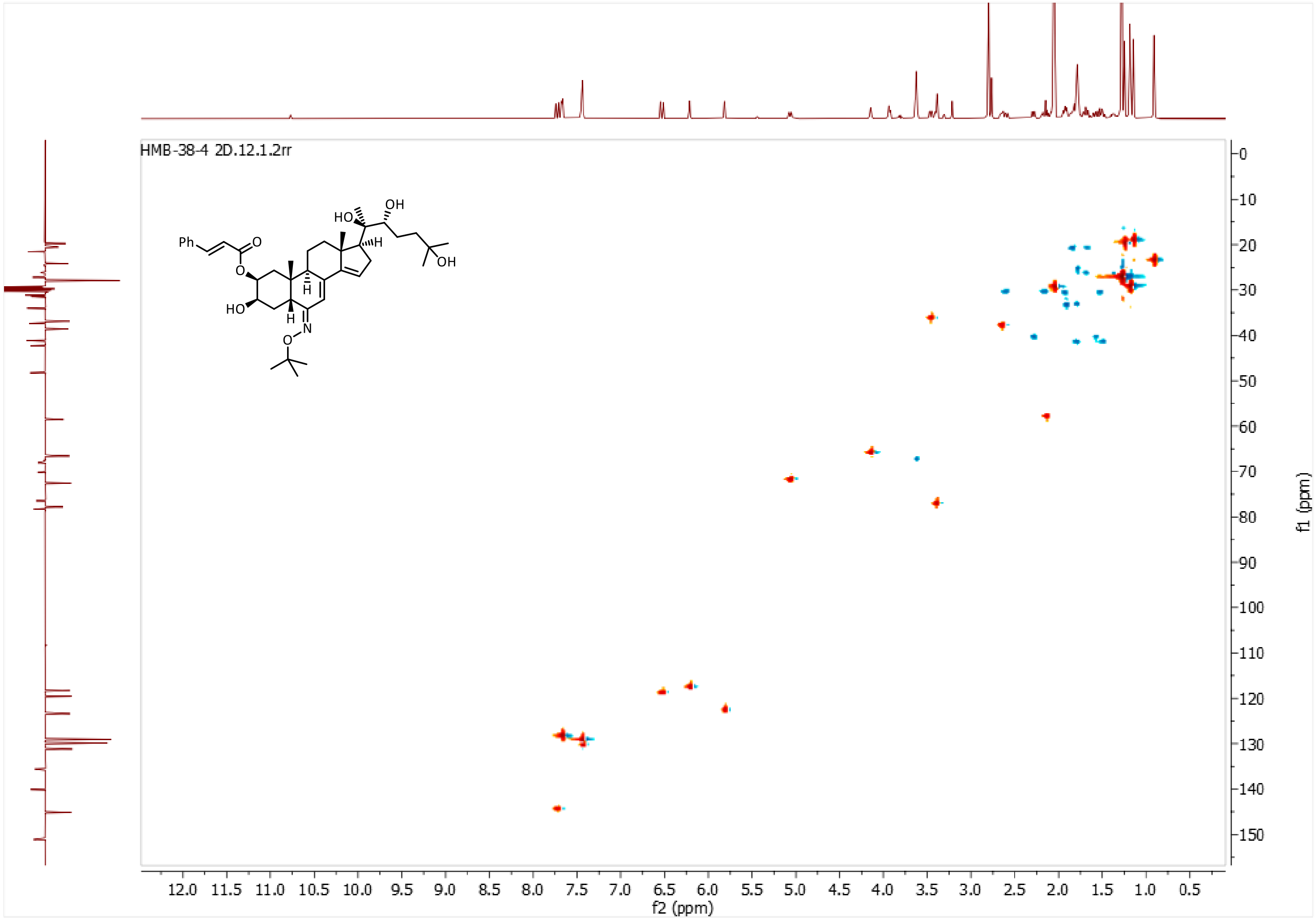

Figure S70. HSQC NMR (acetone-d_6) spectrum of compound **47**
Stachysterone B 6-*O*-*tert*-butyl oxime ether 2-cinnamate (*Z* isomer)

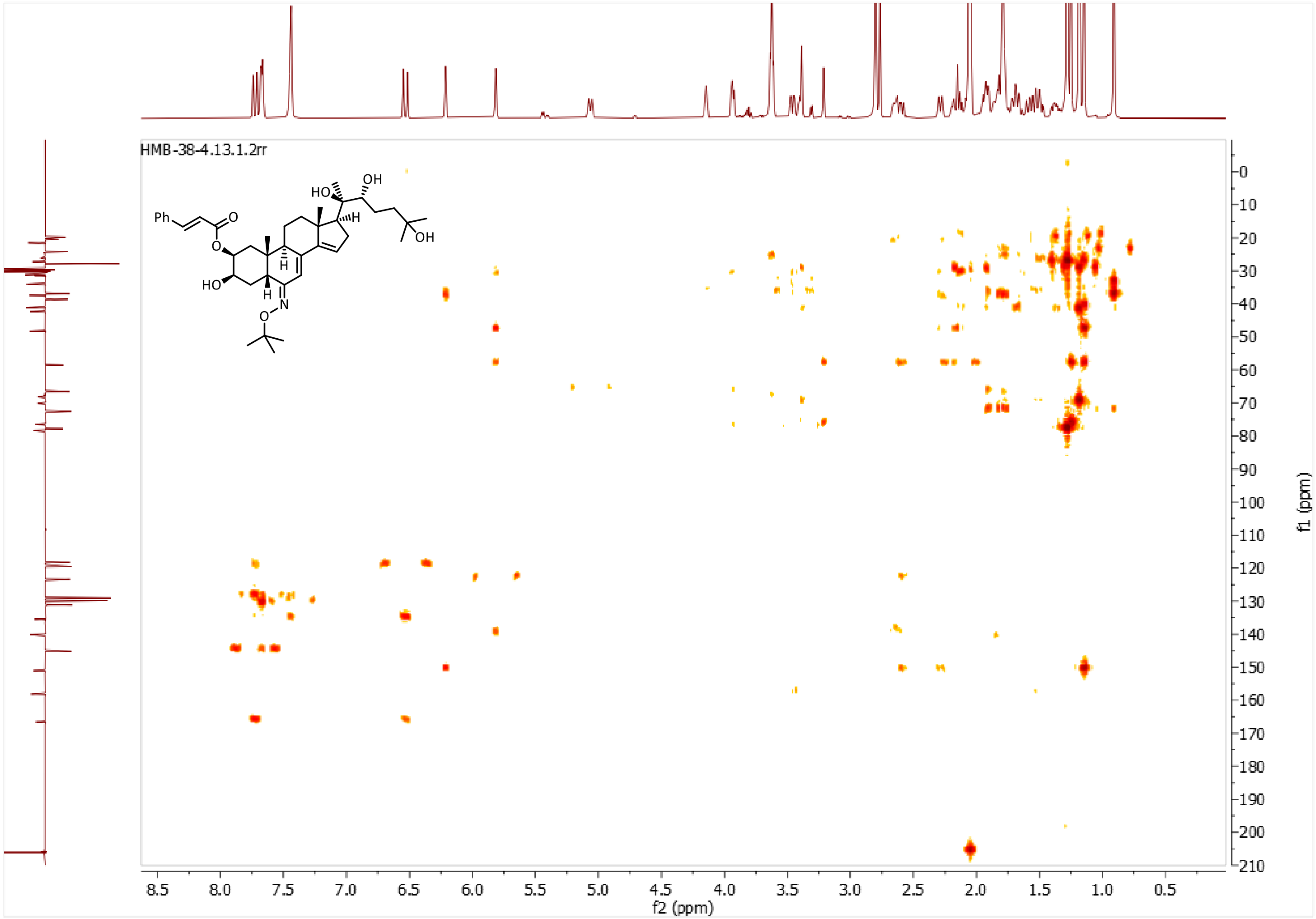

Figure S71. HMBC NMR (acetone-d_6) spectrum of compound **47**
Stachysterone B 6-*O*-*tert*-butyl oxime ether 2-cinnamate (*Z* isomer)

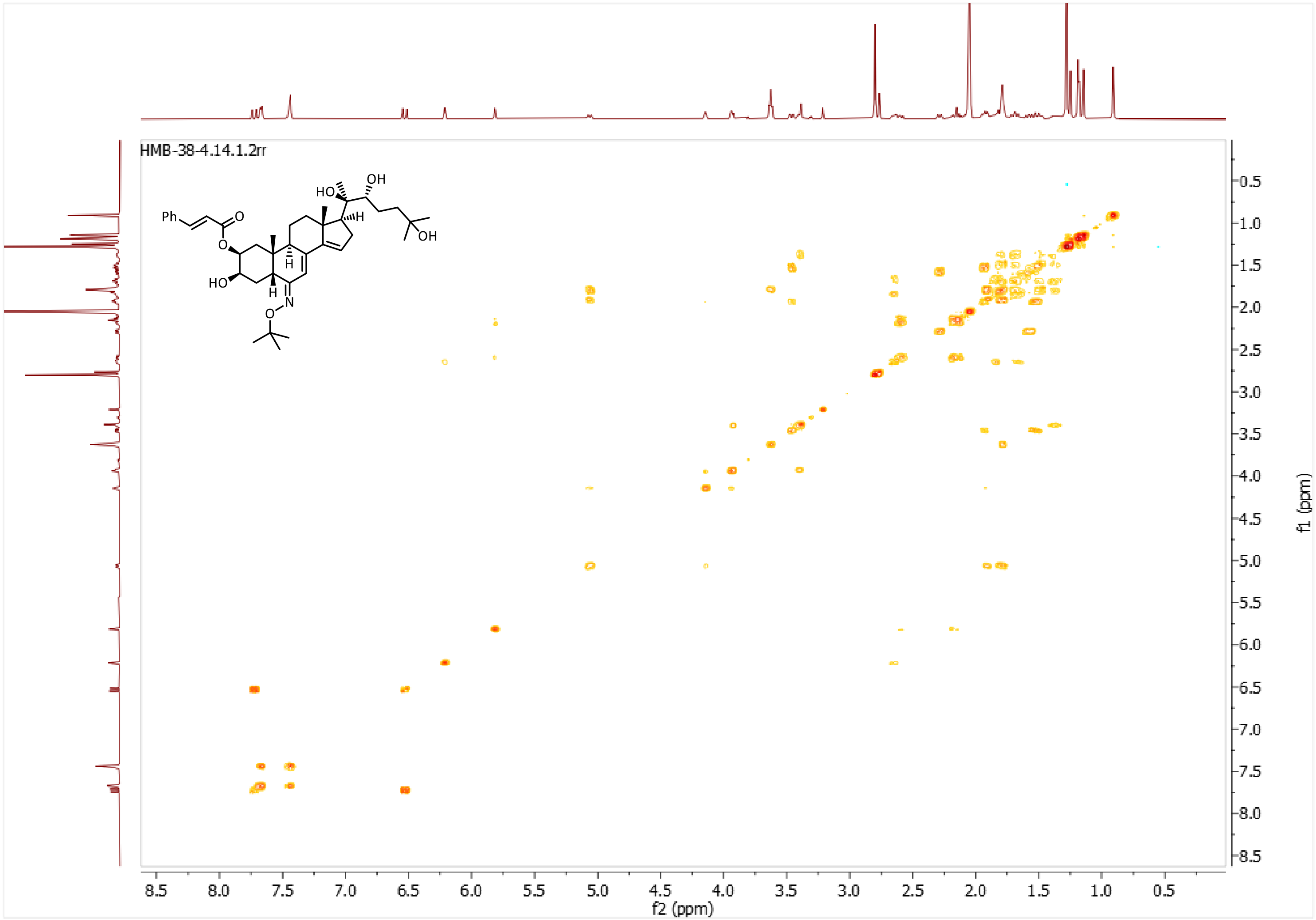

Figure S72. ^{1}H-^{1}H COSY NMR (acetone-d_6) spectrum of compound **47**
Stachysterone B 6-*O*-*tert*-butyl oxime ether 2-cinnamate (*Z* isomer)

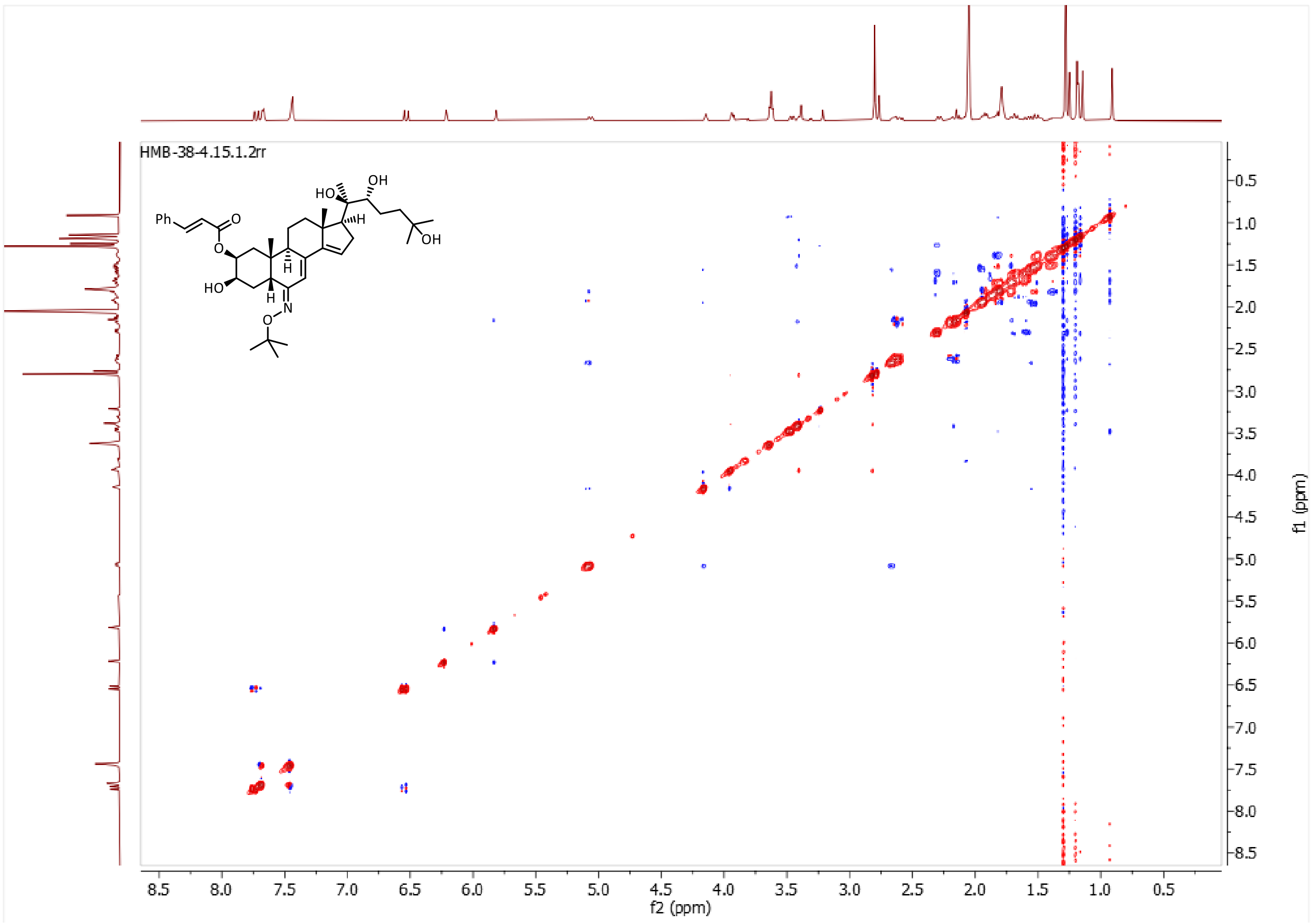

Figure S73. ROESY NMR (acetone-d_6) spectrum of compound **47**
Stachysterone B 6-*O*-*tert*-butyl oxime ether 2-cinnamate (*Z* isomer)

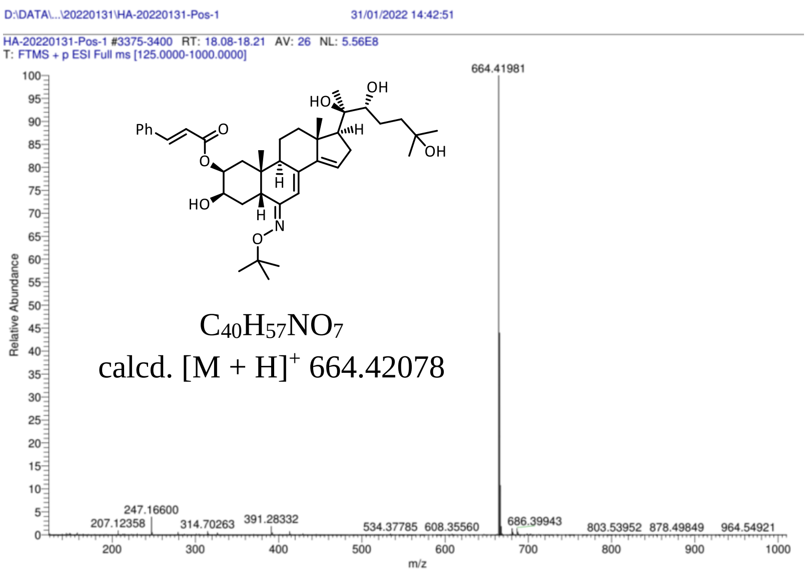

Figure S74. HR-MS spectrum of compound **47**

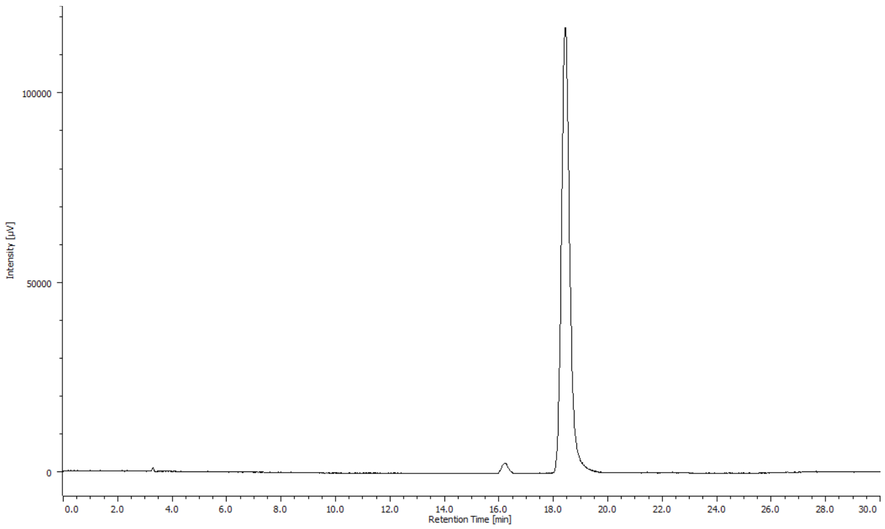

Figure S75. HPLC chromatogram of compound **47** at its UV absorbance maximum (λ=282.6 nm). Purity 98.0 %.

Column: Luna®, 5 µm, Phenyl-Hexyl 100 Å, 250*10 mm (Phenomenex Inc.) Elution: H_2O:CH_3CN (A:B) 62 % B.

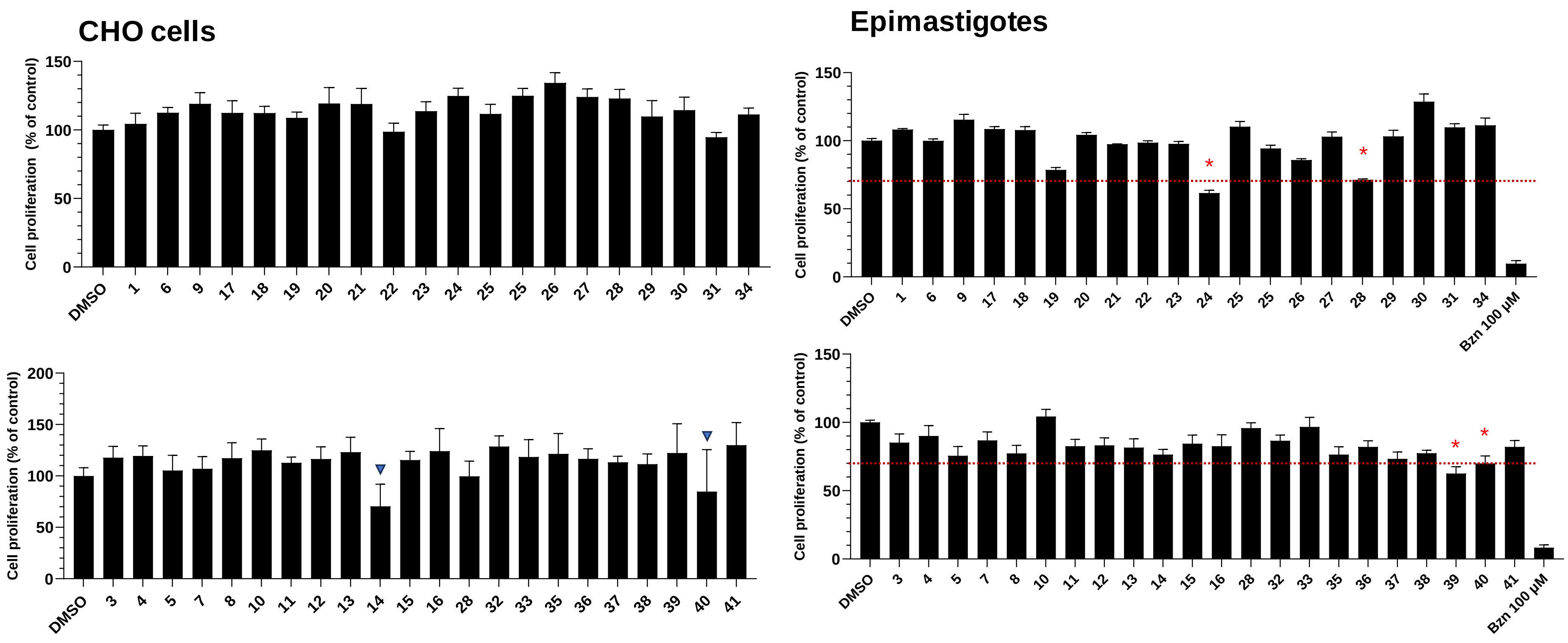

Figure S76. Screening of ecdysteroids for selective trypanocidal effects in CHO cells versus *T. cruzi* epimastigotes at 5 µM.
Blue triangles indicate general cytotoxic effects. Maximal *T. cruzi* epimastigote proliferation inhibitory effect of Benznidazole (Bzn) (100 µM) shown as positive control. DMSO was the vehicle control. * ≥30% inhibition of epimastigote proliferation were considered as positive trypanocidal effect. Data show mean values ± standard deviation of 3 independent experiments each performed in triplicates.
